# Exploiting spatial isomerism to modulate the assembled phase and rheological response of compositionally identical sugar-based surfactants†

**DOI:** 10.1039/d4sc08242g

**Published:** 2025-01-22

**Authors:** Jia-Fei Poon, Alfonso Cabezón, Alessandro Gulotta, Najet Mahmoudi, Stefan Ulvenlund, Rebeca Garcia-Fandiño, Adrian Sanchez-Fernandez

**Affiliations:** a European Spallation Source Box 176 SE-221 00 Lund Sweden; b Center for Research in Biological Chemistry and Molecular Materials (CIQUS), Department of Organic Chemistry, Universidade de Santiago de Compostela Rúa de Jenaro de la Fuente, s/n 15705 Santiago de Compostela Spain; c Division of Physical Chemistry, Lund University Box 124 SE-221 00 Lund Sweden; d ISIS Neutron and Muon Source, Science & Technology Facilities Council, Rutherford Appleton Laboratory Chilton OX11 0QX UK; e Department of Process and Life Science Engineering, Lund University Box 117 SE-221 00 Lund Sweden; f Center for Research in Biological Chemistry and Molecular Materials (CIQUS), Department of Chemical Engineering, Universidade de Santiago de Compostela Rúa de Jenaro de la Fuente, s/n 15705 Santiago de Compostela Spain adriansanchez.fernandez@usc.es

## Abstract

For decades, extensive surfactant libraries have been developed to meet the requirements of downstream applications. However, achieving functional diversity has traditionally demanded a vast array of chemical motifs and synthetic pathways. Herein, a new approach for surfactant design based on structural isomerism is utilised to access a wide spectrum of functionalities. A library of C18-aliphatic maltosides was prepared through Koenigs–Knorr glycosylation, with their properties tuned through anomerism, stereoisomerism, regioisomerism, and the degree of tail unsaturation. Self-assembly of the amphiphiles gave rise to various morphologies, ranging from small micelles to large one-dimensional semiflexible assemblies, which were ultimately defined by the directionality of the supramolecular interactions imposed by the angular restraints of the isomeric centres. Remarkably, the microscopic phase determines the rheological behaviour of the system, which accesses Newtonian solutions, viscoelastic fluids, and gels with customised mechanical properties. The approach outlined in this study serves as a blueprint for the design of novel bioderived surfactants with diverse behaviours without altering the chemical composition of the surfactants, where the understanding of molecular interactions can potentially be used to predict and design the assembly and function of isomerically varied amphiphiles.

## Abstract 2

The modulation of spatial isomerism of sugar-based surfactants enables tailoring their assembly behaviour and the macroscopic response, allowing the development of a variety of functions without altering the chemical composition of the system.

## Graphical abstract

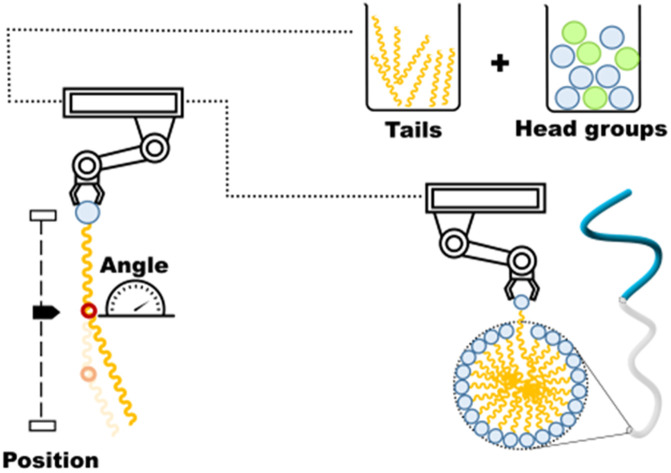

## Introduction

Molecular self-assembly of surfactants into well-defined equilibrium structures is essential for a broad range of applications,^1^ such as daily-use detergents, sophisticated assembled nanoreactors,^2,3^ rheological modifiers,^4^ and controlled drug delivery systems.^5^ This large number of applications requires a vast chemical diversity to meet their expected function, often linked to the structure of the surfactant assemblies.^1^ A canonical approach to guide the structural outcome of the autonomous organisation traditionally relies on introducing significant modifications to the chemistry of the surfactant, which in turn modulates the spontaneous curvature of the assembled structure.^6,7^ For instance, it is well established that ionic amphiphiles with short aliphatic tails form globular micelles in aqueous, non-saline buffers,^8,9^ whereas longer tails attached to neutral head-groups assemble into long, semiflexible worm-like micelles (WLMs).^10^ Other strategies include modification of the exogenous conditions of the system, for example varying the ionic strength or adding amphipathic modifiers.^8^ Given that changes in the chemistry of the system could have deleterious effects on their respective technologies, such as compromising the functionality of drug delivery carriers or leading to protein denaturation,^11–13^ maintaining the delicate balance between achieving the desired function and preserving technological integrity remains a critical challenge in the field.

Molecular isomerism represents an emerging alternative approach to control surfactant self-assembly.^14^ For example, it is well-known that nature utilises isomers of mono- and poly-unsaturated lipids to locally modulate the properties of cell membranes.^15^ Moreover, the stereoisomers of butane-1,2,3,4-tetraol maltosides display different stabilisation capacities of membrane proteins, ultimately attributed to the influence of chirality on surfactant packing at the protein–nanodisc interface.^16^ Biogenic amphiphiles are also known to yield strain- and substrate-specific stereoisomers, which assemble into micelles or supramolecular fibres depending on the isomeric configuration.^17,18^ In the context of artificial surfactants, modifications in the epimerism and anomerism of glycosides have also been shown to tailor the micelle structure and function.^19–21^ In all of these systems, the modification of the unimer topology potentially defines the directionality of the supramolecular interactions, which results in variations in the molecular packing and, concomitantly, the supramolecular structure.^14^ While these reports underscore the promising potential of isomerism to modulate surfactant behaviour, a comprehensive molecular-level understanding of how isomerically varied surfactants assemble and function remains elusive.^22^

**Fig. 1:**
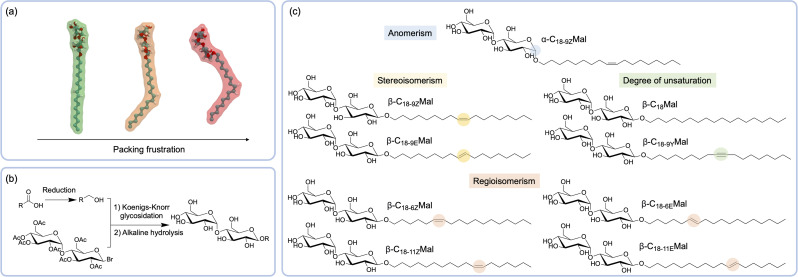
(a) Schematic representation of packing frustration: (*E*)-octadec-9-en-1-yl-β-d-maltoside (green), (*Z*)-octadec-9-en-1-yl-β-d-maltoside (orange), and (*Z*)-octadec-9-en-1-yl-α-d-maltoside (red), where the kinks in the molecular structure are hypothesised to disrupt efficient packing. (b) Synthesis of the surfactants by the Koenigs–Knorr glycosylation reaction. (c) Sugar-based surfactants with varied anomerism, stereoisomerism, regioisomerism, and the degree of tail-unsaturation. Details on the synthesis and characterisation of surfactants are presented in the ESI.†

With the purpose of dissecting the underlying rules that define surfactant behaviour as a function of the unimer isomeric configuration, we designed, prepared, and evaluated a series of maltoside-based surfactants with varied geometry. Our strategy builds upon the concept of packing frustration (Fig. 1a), whereby isomeric variations in the building block modulate the self-assembly through restrictions in the packing. In principle, higher packing frustration is expected to lead to shorter assemblies and *vice versa*. Our preliminary results showed that the glucoside analogue was too insoluble to yield micelle formation (see Fig. S35†). In addition, previous investigations demonstrated that certain disaccharide-based headgroups (*e.g.* cellobiose) also displayed low solubility,^20^ while longer headgroups (*e.g.*, maltotriose) resulted in the formation of globular assemblies that did not provide significant rheological modifications.^23^ As such, we decided to use maltoside headgroups to investigate the effect of isomerism on the assembly and function of compositionally identical surfactants. A library of C18-tailed maltosides, prepared through Koenigs–Knorr glycosylation (Fig. 1b), was used as a model system to explore the influence of the anomeric configuration of the *O*-glycosidic headgroup-tail linker, as well as the regioisomerism, stereoisomerism, and the degree of tail unsaturation (Fig. 1c).

Our findings demonstrate that molecular isomerism profoundly influences the assembly and rheological behavior of surfactants, enabling precise control over the formation of diverse micellar structures with varied elongation and flexibility. These variations arise from intermolecular interactions within the assembled phase, where the angular restraints of different isomers dictate unimer packing and, consequently, the overall behavior. Importantly, the assembled phases govern the macroscopic properties of the system, ranging from Newtonian fluids to viscoelastic fluids and (pseudo)permanent gels with tuneable mechanical characteristics. Given the technological relevance of sugar-based surfactants—owing to their independence from fossil-derived raw materials, low environmental impact, and efficient biodegradability—^24^ our results highlight the potential of these green surfactants to achieve functional versatility through minimal chemical modification.

## Results and discussion

### Krafft points and critical micelle concentrations

To corroborate the possibility of using structural isomerism to regulate surfactant assembly, we prepared a range of tail-unsaturated maltoside surfactants, along with their saturated analogue. In brief, fatty alcohols and acetobromomaltose, prepared respectively from fatty acids and maltose, underwent Koenigs–Knorr glycosylation followed by hydrolysis to yield the corresponding sugar-based surfactants with tailored isomerism. With the compounds in hand, we explored the boundaries for the self-assembly of the surfactants, *i.e.*, the Krafft point, cloud point, and critical micelle concentration (CMC). Consistent with previous findings, we observed that β-C_18_Mal exhibits extremely low solubility in aqueous solution, with a Krafft temperature above 90 °C.^25^ In contrast, the unsaturated C18-maltoside analogues were readily soluble in water, resulting in the formation of homogeneous, transparent solutions. Differential scanning calorimetry (DSC) measurements indicate that the Krafft points are below 5 °C and the cloud points above 90 °C (Fig. S36†). The depression in the Krafft point of unsaturated surfactants compared to that of saturated analogs is well-established in the literature.^20,26^ Notably, the absence of high-temperature clouding presents an advantage over many technologically relevant nonionic surfactants (*e.g.*, polysorbates),^27^ thus holding significant implications for their potential applications that require stabilised assembled phases at elevated temperatures.

CMCs were determined spectroscopically at 25 °C using the 1-pyrenecarboxaldehyde assay (Fig. S37†).^28^ The CMCs of the unsaturated surfactants were found to be in the μM range (Table 1), which are comparable to those from other commercial nonionic surfactants (*e.g.* 50 μM for polysorbate 20 and 60 μM for Brij 35).^29^ However, the CMC of β-C_18_Mal could not be determined experimentally and was estimated to be sub-μM by extrapolation methods.^30,31^ The installation of tail unsaturation was required to increase surfactant solubility sufficiently to allow micelle formation. Importantly, significant variations in the CMC were observed due to changes in the surfactant *stereo* configuration. For instance, the CMCs of the *trans*-configured alkenyl maltosides are 2-fold higher than those of the corresponding *cis*-isomers. These observations agree with the higher CMC observed for elaidic acid (*trans*-unsaturated) compared to oleic acid (*cis*-unsaturated),^32^ which is attributed to the configurational entropy of the tail. Conversely, the 2.5-fold higher CMC observed for α-C_18-9Z_Mal compared to its β-counterpart follows an opposite trend to many other anomerically varied sugar-based surfactants, *e.g.*, glucosides,^33^ galactosides,^34^ and maltosides.^30,35^ This could be attributed to a synergistic effect of the *cis*-configured tail with the α-anomerism that increases the solubility of the monomer. Besides, the introduction of an alkyne moiety (*i.e.*, β-C_18-9Y_Mal) yields a comparable CMC to those of the *trans* analogs. This is possibly related to the structural similarity between the *trans* and tri-unsaturated tails, as they both adopt a linear configuration. These observations agree with Engberts' report on the increased CMC of surfactants with linear unsaturated tails.^36^ In contrast, regioisomers (*i.e.*, 6-, 9- and 11-isomers) showed no discernible changes on the CMC within the margin of error. Collectively, the CMC results provide preliminary evidence that structural isomerism can trigger changes in surfactant behaviour.

**Table 1:** CMC of sugar-based surfactants determined using the 1-pyrenecarboxaldehyde assay

Surfactant	CMC/μM	Surfactant	CMC/μM
α-C_18-9Z_Mal	50 ± 3	β-C_18-6Z_Mal	20 ± 3
β-C_18-9Z_Mal	19 ± 3	β-C_18-11Z_Mal	23 ± 1
β-C_18-9E_Mal	38 ± 1	β-C_18-6E_Mal	37 ± 2
β-C_18-9Y_Mal	38 ± 2	β-C_18-11E_Mal	42 ± 2

Overall, these surfactants exhibit highly desirable characteristics for potential technological applications, including low CMC values and assembled phases that remain stable across the entire temperature range of liquid water.

### Packing frustration dictates micelle morphology

**Fig. 2:**
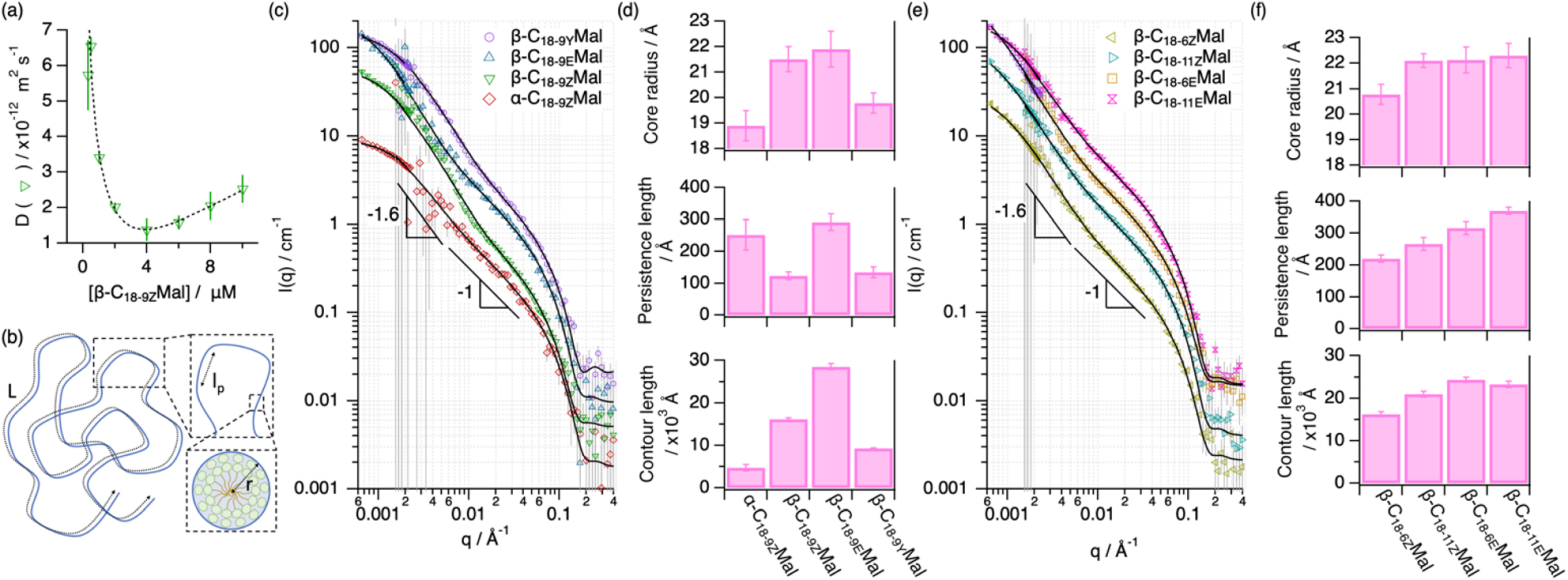
(a) Concentration dependence of the diffusion coefficient of β-C_18-9Z_Mal micelles. The dotted lines represent the best fit using a log-normal function used to determine the minimum of the function. (b) Schematic representation of the hierarchical structure of WLMs: *L* – contour length, *l*_p_ – persistence length, and *r* – core radius. SLS (*q* < 0.0023 Å^−1^) and SANS (*q* > 0.0016 Å^−1^) data (markers as indicated in the legend of the graphs) and models (solid lines) of: (c) α-C_18-9Z_Mal, β-C_18-9Z_Mal, β-C_18-9E_Mal, and β-C_18-9Y_Mal, and (e) β-C_18-6Z_Mal, β-C_18-11Z_Mal, β-C_18-6E_Mal, β-C_18-11E_Mal, at 1 mM in D_2_O. Data and models have been offset for clarity and, where not seen, error bars are within the markers. (d) and (f) Structural parameters of the micelle derived from the simultaneous analysis of the SANS and SLS data presented in (c) and (e) respectively. The error bars represent the standard deviation of the average values. Details on the analysis of the scattering data are presented in the ESI† and a full record of the parameters is presented in Table S1.†

With the boundaries of the self-assembly established, the morphology of the micellar phase in water was investigated by small-angle neutron scattering (SANS) and static light scattering (SLS) at 25 °C. In order to directly extract information regarding the structure of the micelles, the characterisation was conducted in the dilute regime where intermicellar interactions are negligible.^21,37^ The apparent diffusion coefficient of β-C_18-9Z_Mal at various concentrations was measured using dynamic light scattering (DLS), which allowed us to determine the overlap concentration at which micelles begin to collectively interact (Fig. 2a). Plotting the micellar diffusion coefficient against concentration revealed a distinctive parabolic shape. According to the stepwise self-association model,^38^ one would anticipate a continuous decrease in diffusion due to micelle growth as surfactant concentration increases. However, the observed reversal in behaviour confirms the emergence of intermicellar correlations,^39^ leading to seemingly accelerated dynamics due to the network breathing upon entanglement.^40^ The transition vertex delineates the overlap concentration for β-C_18-9Z_Mal, occurring at 3.6 ± 0.2 mM. In addition, the emergence of a slow relaxation mode (lag time >0.01 s) is observed above 4 mM, associated with intermicellar interactions (Fig. S38†). Due to the molecular similarities between the surfactant unimers, we expected the transition to the semi-dilute regime to occur at similar concentrations for all surfactants in the current study. For this reason, SANS and SLS investigations were performed at 1 mM surfactant concentration. Based on the features of the scattering curve – a slope of −1 for 0.008 Å^−1^ < *q* < 0.03 Å^−1^ that evolves to −1.6 approximately at *q* = 0.004 Å^−1^ – the flexible cylinder was selected as the most appropriate model for data analysis.^41^ This model enables the resolution of the WLM hierarchical structure: the radius of the cross-section, contour length, and persistence length (Fig. 2b). The contour length corresponds to the end-to-end distance of the micelle and the persistence length accounts for the statistical length over which micellar segments appear straight in the presence of thermal fluctuations, thus associated with micellar flexibility.^42^

In line with the expected influence of structural “kinks” on the self-assembly,^10^ a 1.8-fold increase of the contour length was observed upon replacing the *stereo*-configuration of the tail unsaturation from *cis* to *trans* (*i.e.*, β-C_18-9Z_Mal*vs.*β-C_18-9E_Mal), whereas introduction of an alkyne moiety to the aliphatic tail, *i.e.*, β-C_18-9Y_Mal, resulted in significantly shorter micelles. Overall, our results agree well with the concept of packing frustration, whereby an increasing number of non-linear connectors causes a shortening in the micelle contour length.^10^ Presumably, variations in micelle elongation arise from differences in unimer packing.^38,43^ In fact, these differences in unimer packing are confirmed by the changes in the radius of the micelle cross-section (Fig. 2d), which follows the same trend.

### β-C_18-9E_Mal > β-C_18-9Z_Mal > β-C_18-9Y_Mal > α-C_18-9Z_Mal

The results also showed that the geometric configuration of the sugar-based surfactants affects the micelle flexibility, which is reflected by the difference in persistence length (Fig. 2d). Shorter micelles formed by α-C_18-9Z_Mal were more rigid than those of β-_C18-9Z_Mal due to the entropic effects.^44^ Unexpectedly, WLMs of β-C_18-9E_Mal were more rigid than those of β-C_18-9Z_Mal, despite the *trans*-analogue forming much longer assemblies. This trend reversal is likely attributed to a more efficient packing of the *trans*-analogue;^45^ the better alignment of the linear tail of the *trans*-isomer effectively causes a more conformationally arrested assembly. Interestingly, β-C_18-9Y_Mal exhibits a high flexibility despite its relatively short contour length.

Next, we sought to further leverage the behaviour of the β-maltosides through regioisomerism of tail-unsaturation (Fig. 2e). In general, the differences in the micelle contour length between the regioisomers were less pronounced than those for the different anomers and stereoisomers. In particular, the assemblies of β-C_18-6E_Mal, β-C_18-9E_Mal and β-C_18-11E_Mal display similar contour lengths and cross-section radii (Fig. 2f), possibly associated with the linear configuration of the tail.^45^ Among the *cis*-isomers, shorter micelles were formed when unsaturation was located at the 9-position, followed by the 6-position and 11-position. This is likely associated with the changes in the configurational entropy of the tail, where packing frustration is expected to be largest when positioning the unsaturation at the centre of the tail.^46^ Interestingly, the position of the unsaturation was also shown to significantly affect the flexibility of the assemblies. The *trans*-configured surfactants consistently formed stiffer micelles than the *cis*-analogues, and the differences in the position of the *cis*-unsaturations yielded larger changes in the micelle persistence length than those for the *trans*-configured surfactants (Fig. 2f).

### Supramolecular interactions defined by surfactant topology

**Fig. 3:**
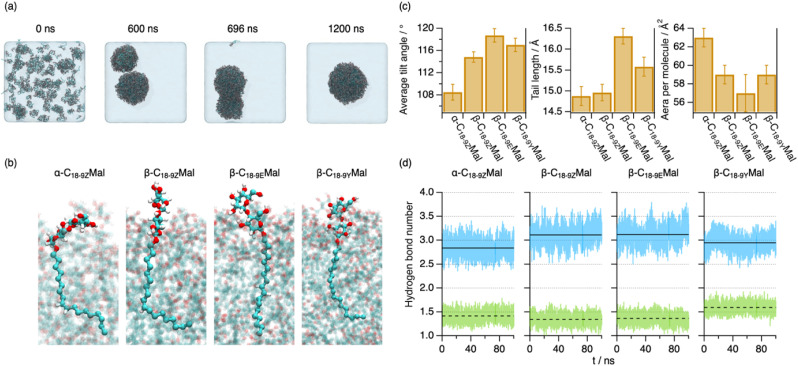
(a) Representative snapshots of the MD simulation trajectory of α-C_18-9Z_Mal conducted at 1 bar and 298.15 K at different simulation times. The snapshot at 696 ns represents the time point at which discrete pre-assemblies fuse to form a larger micelle. (b) Details of each surfactant type in the micelle extracted from snapshots at *t* = 1300 ns. The other surfactants within the micelle are rendered transparent to enhance visibility. (c) Structural parameters of the unimer averaged over the last 100 ns of the simulations: average tilt angle, tail length, and area per molecule at the micelle interface. The error bars represent the standard deviationfrom the average values during the last 100 ns of the simulation. (d) Instant (blue and green lines) and time-averaged (black lines) number of hydrogen bonds per headgroup in the micellar phase for each surfactant. The dashed lines represent hydrogen bonds between headgroups, while the solid lines represent hydrogen bonds between headgroups and water molecules. These data are shown for the last 100 ns of the simulation and parameters have been averaged for all the unimers within the micelle.

To elucidate the molecular origin of the differences in the micelle structure, we performed all-atom molecular dynamics (MD) simulations on the surfactants with different anomerism, stereoisomerism, and degrees of unsaturation. Initially, the molecules were randomly distributed within the box; the spontaneous assembly and equilibration into micelles were observed at *ca.* 1300 ns (Fig. 3a and S39†). Subsequently, data analysis was performed over the last 100 ns of the simulation (see the ESI† for details). It should be noted that these simulations were carried out using a reduced number of surfactant molecules (400 unimers per micelle) compared to the experimentally observed large assemblies (>10^4^ unimers per micelle), which could not be feasibly resolved using all-atom simulations. However, this approach was deemed suitable since it is well-established that the assembly of WLMs is preceded by the formation of globular micelles at surfactant concentrations slightly above the CMC,^47^ which subsequently grow by accretion of surfactant molecules with increasing concentration. In fact, our simulations show the formation of small assemblies at early simulation times that subsequently fuse into a larger, asymmetric micelle at longer times (Fig. 3a and S39†). The intermolecular interactions observed at this scale are expected to be reflective of surfactant behaviour at higher surfactant concentrations, *i.e.*, upon the assembly into WLMs. In terms of surfactant behaviour at early stages of micellisation, it should be noted that the concentrations used in this study do not allow extraction of information about characteristic parameters, such as the CMC, and thus we rely on our experimental characterisation for this information.

Snapshots of individual surfactants within the micelle show significant differences between their structures (Fig. 3b). The averaged tilt angles of the surfactants (*i.e.*, torsion with respect to the normal projection) are smaller for unimers involving angular restraints of axial configurations, *i.e.*, α- and *cis*-configurations, compared to those with equatorial configurations, *i.e.*, β- and *trans*-configurations (Fig. 3c), in line with that observed for unsaturated fatty acids.^45^ Thus, the calculations showed that the less pronounced angular restraints observed for β-C_18-9E_Mal allow for more efficient packing, while the highly curved conformation of α-C_18-9Z_Mal decreased the efficiency of the packing. This also aligns with the average length (in a linear projection) of the hydrophobic tail in the assembled state, wherein greater values were obtained for linear tails of β-C_18-9E_Mal compared to its *cis*-configured counterpart (Fig. 3c). As expected, the average area-per-molecule at the micelle interface is mainly defined by the configuration of the headgroup-tail *O*-glycosidic linkage,^30^ where the axial (α) configuration results in a larger area per molecule than the equatorial (β) configurations. According to the rationale of the packing parameter,^6^ we expect from our MD results that micelle elongation would vary following the trend α-C_18-9Z_Mal < β-C_18-9Z_Mal < β-C_18-9E_Mal, which agrees well with our experimental findings. Seemingly, β-C_18-9Y_Mal does not follow the same principles compared to the alkenyl analogs since its area per molecule and tail length would predict a similar morphology to that of β-C_18-9Z_Mal. However, the implicit change in the configuration of the tail could unbalance the framework to describe surfactant packing,^6^ resulting in an unexpected association.

The interactions between headgroups are key to defining the area per molecule at the micelle interface. To resolve the origin of this effect, we determined the population of headgroup–headgroup and headgroup-water H-bonding.^48^ Our results reveal that changes in the anomeric configuration have the largest impact compared to those in tail stereoisomerism (Fig. 3d). α-C_18-9Z_Mal shows a lower degree of hydration and more H-bonding between neighbouring headgroups compared to the β-configuration. The lower hydration coupled with a larger area per unimer in α-C_18-9Z_Mal hinted that the steric effects greatly influence the packing of the monomer. Thus, the angular restraint of the *O*-glycosidic linkage is presumably the major cause for the formation of the shorter micelles derived from α-C_18-9Z_Mal. This contrasts with previous hypotheses where differences in the micellar shape were linked to a lower degree of hydration for β-C_16_Mal compared to α-C_16_Mal.^30^ Interestingly, a lower degree of hydration was also observed in β-C_18-9Y_Mal compared to the other β-configured surfactants, which suggests that changes in the degree of unsaturation can strongly impact intermolecular interactions, and thus packing, despite bearing the same headgroup.

Undoubtedly, the extent of packing frustration provides a valuable proxy to predict the assembly of the isomerically varied surfactants from a molecular perspective: (1) the packing of *trans*-configured aliphatic tails is more efficient due to the lower configurational entropy than that of *cis*-analogs;^45^ (2) the headgroups linked to the tail through a β-configuration are expected to form tightly packed domains, while the tilt of the α-anomer sterically frustrates the packing of the sugar units.^21,49^ Consequently, the more efficient the packing, the longer and stiffer the assembly grows. Besides, changes in the degree of unsaturation will impact the overall packing due to specific intermolecular interactions in the aliphatic domain of the micelle.

### Rheological modulation through surfactant isomerism

With these design possibilities in hand, we further explored the macroscopic response of the system by employing rheology as a functional proxy. The structural characterisation conducted thus far has primarily focused on micelles in the dilute regime (Fig. 2b), where intermicellar correlations are anticipated to be negligible. To investigate the macroscopic response of the micelles, rheological measurements were conducted at concentrations well above the overlap concentration, *i.e.*, 10, 25, 37.5, and 50 mM, where topological interactions between micelles could impart a non-Newtonian response to the system.^37,50^

**Fig. 4:**
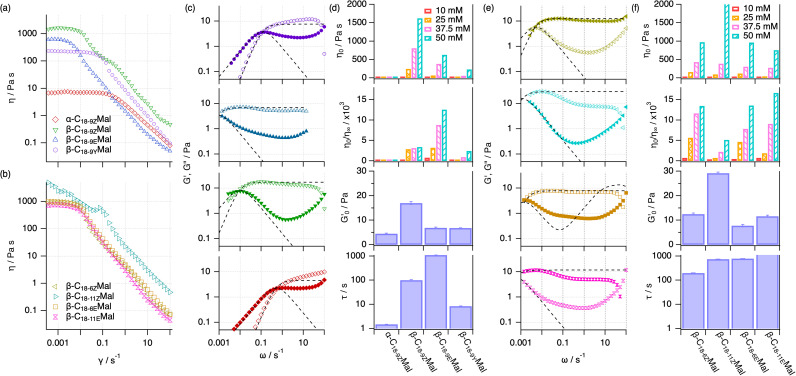
Steady shear viscosity measurements of the surfactants at 50 mM concentration: (a) α-C_18-9Z_Mal, β-C_18-9Z_Mal, β-C_18-9E_Mal, and β-C_18-9Y_Mal, as well as (b) β-C_18-6Z_Mal, β-C_18-11Z_Mal, β-C_18-6E_Mal and β-C_18-11E_Mal, as indicated in the legend of the graphs. (c) and (e) Elastic (*G*′, open symbols) and viscous (*G*′′, filled symbols) moduli obtained from frequency sweep measurements at 50 mM surfactant concentration for the systems shown in (a) and (b) respectively. (d) and (f) Summary of the rheological properties of the surfactants in (a and c) and (b and e) respectively: zero-shear viscosity (*η*_0_) and relative drop in viscosity (*η*_0_/*η*_∞_) of various sugar-based surfactants at 10, 25, 37.5 and 50 mM, (as shown in the legend of the graph) plateau of the elastic modulus at high frequencies 
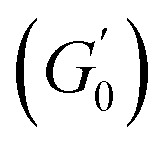
 and relaxation time (*τ*) at 50 mM surfactant concentration. All experiments were conducted at 25 °C. Details on the analysis of the rheology data are presented in the ESI.†

Linear rheology with controlled shear stress assessed the viscosity of the samples under shear. Although a shear thinning character is observed for all these surfactants at 50 mM (Fig. 4a and b), *i.e.*, the viscosity gradually decreases due to the network rearrangement under shear,^37^ the rheological profiles strikingly change between the different surfactants. For example, the zero-shear viscosity (*η*_0_) of β-C_18-9Z_Mal increased by *ca.* 270-fold compared to that of α-C_18-9Z_Mal at 50 mM upon alternation of the anomeric configuration (Fig. 4d). Furthermore, a significantly larger drop in the relative viscosity (*η*_0_/*η*_∞_) was observed for β-C_18-9Z_Mal (*ca.* 3000) compared to its α-counterpart (*ca.* 80). The difference could be further amplified by decreasing the concentration of the surfactant to 25 mM (Fig. S40†), where α-C_18-9Z_Mal affords a low viscosity Newtonian fluid (*η*_0_/*η*_∞_ ≈ 1) while β-C_18-9Z_Mal retains its shear-thinning character (*η*_0_/*η*_∞_ ≈ 3000). Variations in the *stereo*-configuration of the tail unsaturation were found to affect the rheology of the system, *e.g.*, β-C_18-9E_Mal profiled a 0.38-fold lower zero-shear viscosity and a 4-fold higher relative drop in viscosity compared to β-C_18-9Z_Mal. Moreover, a drastic decrease in zero-shear viscosity was observed when the double bond was replaced with a triple bond (*i.e.*, β-C_18-9Y_Mal). In terms of the impact of regioisomerism on the rheological response, *cis*-configured surfactants consistently showed higher viscosity values and lower relative viscosity drops than the *trans*-analogs (Fig. 4f), following the trend β-C_18-6Z_Mal < β-C_18-9Z_Mal < β-C_18-11Z_Mal. In contrast, the three *trans*-configured regioisomers displayed less pronounced differences. This could be again attributed to the configuration of the tail, as expected from the structural similarity between the micelles of the *trans*-configured surfactants compared to that of their *cis*-counterparts (Fig. 2d and f).

The rheology of the surfactants is intricately connected to the changes in elongation, flexibility, and collective behaviour of the micelles. The shorter and stiffer α-C_18-9Z_Mal micelles result in fewer entanglement points and, concomitantly, low viscosity fluids. In contrast, β-C_18-9Z_Mal forms 1D assemblies with lengths on the micron scale, leading to intricate micellar networks. To the best of our knowledge, the *cis*-configured surfactants were found to be the most viscous sugar-based surfactant systems reported to date.^20,26^ Another valuable example of the interplay between contour length and flexibility in defining rheology is observed for β-C_18-9Y_Mal, which yields a relatively high zero-shear viscosity despite forming shorter assemblies compared to, *e.g.*, β-C_18-9E_Mal.

To our surprise, the more elongated β-_C18-9E_Mal micelles were found to be less viscous than those of β-C_18-9Z_Mal. This could be attributed to: (1) the stiffer β-C_18-9E_Mal micelles provide fewer entanglement points, causing a lower mesh density,^39^ or (2) micelle branching of the *trans*-analogue adds another relaxation mechanism compared to that of the unbranched *cis*-surfactant.^51^ Further proof of the different relaxation mechanisms between these systems is observed in the viscosity curves. For instance, β-C_18-11Z_Mal shows deviations from the linearity during the viscosity drop (Fig. 4b and S7†), which are not observed for any of the *trans*-unsaturated surfactants. This is likely attributed to the presence of shear-induced structures: domains that arise during micro-scale phase-separation upon flow, which transiently alter the relaxation mode of the system.^20^

To elucidate the mechanism behind the rheological modification between the different surfactants, frequency-sweep oscillatory rheology was performed at a concentration of 50 mM (Fig. 4c). In this analysis, the storage modulus (*G*′) and loss modulus (*G*′′) respectively describe the elastic (solid-like) and frictional (liquid-like) losses of the system. All compounds examined in this study exhibited some degree of viscoelasticity at the 50 mM surfactant concentration, albeit with different properties. The deviation observed at higher frequencies of the loss modulus is attributed to the Rouse diffusion of the WLMs.^40^β-C_18-9Z_Mal displayed the characteristic Maxwellian viscoelastic behaviour, with a dominant storage modulus plateauing at high frequencies (*ω* > 0.05 s^−1^) and an intersection between moduli at low frequencies (*ω* ≈ 0.01 s^−1^). A major weakening in the gel-properties of the system occurred for the variants α-C_18-9Z_Mal and β-C_18-9Y_Mal, which exhibited a decrease in the elastic component at all frequencies, along with a shift of the cross-over to higher frequencies (*ω* ≈ 0.5 s^−1^). In contrast, β-C_18-9E_Mal displayed typical gel behaviour, which is characterised by a strong contribution from the elastic component at all frequencies and an intersection occurring beyond the range of experimentally accessible frequencies (*ω* < 0.001 s^−1^). Notably, the surfactant concentration had to be reduced to as low as 10 mM to find an intersection that is experimentally accessible for these assembled systems (Fig. S41†). The viscoelastic properties of these systems were quantitatively compared using the single stress relaxation time (*τ*) and the plateau of the elastic modulus at high frequencies 
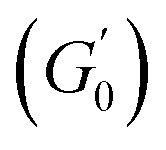
 derived from the Maxwell model (Fig. 4d). The relaxation time is indicative of the average length of entangled micelles, while the plateau elastic modulus reflects the entanglement density of the mesh.^50^ As such, these characteristic parameters provide further insights into the mechanisms underlying the differences in the rheological properties of the surfactants. The higher 
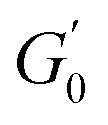
 value for β-C_18-9Z_Mal confirms the formation of a highly entangled network compared to β-C_18-9E_Mal. Conversely, the latter exhibits longer relaxation times (>1000 s) due to the presence of longer WLMs. The pseudo-permanent gel of β-C_18-9Z_Mal,^52^ represents the most mechanically resilient micellar fluid reported to date based on sugar-based surfactants.^20,21,26^ In contrast, the less entangled micelles derived from α-C_18-9Z_Mal and β-C_18-9Y_Mal demonstrate significantly faster relaxations and weaker mechanical components.

In terms of regioisomerism, oscillatory rheology revealed that the *trans*-configured surfactants display a similar rheological profile, with only subtle differences in the relaxation times following the trend β-C_18-6E_Mal < β-C_18-9E_Mal < β-C_18-11E_Mal (Fig. 4f). In contrast, the *cis*-configured regioisomers exhibit non-monotonic variations in the rheological parameters, possibly due to the much higher flexibility when locating the unsaturation at the centre of the tail. Interestingly, we found that the deviation from the Maxwell model at high frequencies becomes more prominent for β-C_18-11Z_Mal and β-C_18-11E_Mal compared to the other regioisomers (Fig. 4d), as evidenced in the normalised Cole–Cole plot (Fig. S42†), which could potentially be attributed to differences in the scission and reptation components of the relaxation compared to the other regioisomers.^50^

Our observations underscore the importance of the geometry of the surfactant to define the hierarchical structure of the micelle and, ultimately, the rheological response of the system. Surfactants with an expected high packing frustration, *e.g.*, α-C_18-9Z_Mal (Fig. 1a), lead to the formation of short assemblies due to a relatively low packing efficiency. Concomitantly, α-C_18-9Z_Mal displays low viscosities, even forming Newtonian fluids at comparatively high surfactant concentrations (*e.g.*, 25 mM). Moreover, changing the anomeric configuration to β-C_18-9Z_Mal decreases the frustration as the headgroups can arrange more regularly at the micelle interface. This results in longer and more flexible micelles, which entangle in the semi-dilute regime and yield a mechanically strong viscoelastic fluid. Introduction of the more linear *trans*-unsaturated aliphatic domain (*i.e.*, β-C_18-9E_Mal) promotes a more efficient unimer packing, which ultimately leads to the formation of gels. Finally, the exceptionally long relaxation time followed by strategically relocating the unsaturation to the 11-position of the tail (*i.e.*, β-C_18-11E_Mal) demonstrated the importance of regioisomerism in fine-tuning the rheology of the system. The possibility to utilise anomerism, stereoisomerism, and regioisomerism of the surfactants to define the assembly behaviour and rheology of the system is undoubtedly advantageous for tuning the macroscopic function of the system without altering its chemical composition.

## Conclusion

Molecular isomerism is commonly exploited in biogenic and artificial molecular building blocks to tailor supramolecular behaviour and macroscopic function. Although variations in the behaviour of isomerically varied amphiphiles have been reported, the design principles governing the assembly and function of surfactants with different isomerism have remained elusive until now. Here, we have reported a platform for the modulation of the behaviour of maltoside-based surfactants through molecular isomerism (anomerism, stereoisomerism, and regioisomerism) and degree of unsaturation. The synthesis through a common route yields a library of unsaturated surfactants with high solubility and temperature-resilient assembly compared to their saturated analogue and other surfactants. The variation in isomerism leads to a wide range of assembled structures, from small nanoscale assemblies to micron-length one-dimensional micelles. Critically, we show that changes in the molecular structure dictate packing frustration, where non-linear structures hinder efficient packing through steric hindrance and conformational entropy, leading to the formation of shorter assemblies. The assembled structures translate into different macroscopic responses, ranging from low viscosity Newtonian systems for the shorter micelles, to the formation of highly viscous gels promoted by an entangled network of worm-like micelles.

Our study delves into the interaction–structure–function relationship of these systems, which can be rationalised in terms of molecular topology and extended to the macroscopic response. This breakthrough not only paves the way for the advancement of surfactant development but also enhances our comprehension of the behaviour of biologically derived surfactants.^14,17,22^ We envisioned that the same strategy can be utilised for amphiphiles beyond sugar-based surfactants, as the introduction and modulation of isomeric centres can be conveniently implemented in a broad range of molecules. Ultimately, our platform demonstrates great potential in terms of technological development, where the macroscopic response of the system can be tailored without altering the chemical composition of the system. In fact, this could be exploited in health-related areas,^53^ where the non-toxic, biocompatible character of sugar-based surfactants combined with their “design” prospects can benefit pharmaceutical formulation, drug delivery, and scaffolding.

## Experimental

The alkylmaltosides were synthesised according to procedures based on Koenigs–Knorr glycosylation, purified by flash column chromatography using silica gel, and characterised by NMR and high-resolution mass spectrometry. A Probe Drum spectrophotometer and a MicroCal VP-DSC were employed for CMC and Krafft/cloud point determination, respectively. SLS and DLS experiments were conducted on a 3D-DLS spectrometer. SANS experiments were conducted on Sans2d (ISIS Pulsed Neutron and Muon Source, UK) accessed through Xpress Access.^54^ The raw data are available at DOI: https://doi.org/10.5286/ISIS.E/ISIS.E.RB2190137-2. Molecule parametrisation was performed using GROMOS 54A7 and MD simulations were conducted using the GROMACS 2022.1 software package.^55^ All calculations involved in the MD simulations were performed at the Galicia Supercomputing Center (CESGA). The rheological characterisation was carried out on an Anton Paar MCR 301. A detailed description of the experimental protocols and characterisation results are presented in the ESI.†

## Data availability

Data for this article, including small-angle neutron scattering, light scattering, and rheology data, are available *via* Zenodo at https://doi.org/10.5281/zenodo.13337328.

## Author contributions

A. S.-F. and S. U. conceived the idea. J.-F. P., A. C., A. G., N. M., R. G.-F. and A. S.-F. designed the experiments. J.-F. P. synthesised the surfactants and carried out the CMC, DSC and rheology measurements. A. G. conducted the DLS and SLS measurements. A. C. and R. G.-F. carried out the MD simulations. N. M. performed the SANS experiments. J.-F. P. drafted the manuscript with contributions from A. S.-F. All authors discussed and edited the final version of the manuscript.

## Conflicts of interest

There are no conflicts to declare.

## Supplementary Material

SC-016-D4SC08242G-s001
