# Supplementary material for: Exploiting spatial isomerism to modulate the assembled phase and rheological response of compositionally identical sugar-based surfactants
Source: Chem Sci. 2025 Jan 22;16(9):4048–56. doi: 10.1039/d4sc08242g (PMC11775819; doi:10.1039/d4sc08242g)
Supplement: SC-016-D4SC08242G-s001 [file SC-016-D4SC08242G-s001.pdf]

Supplementary Information (SI) for Chemical Science.
This journal is © The Royal Society of Chemistry 2025

Supporting Information for:

Exploiting spatial isomerism to modulate the assembled phases of compositionally identical sugar-based surfactants

Jia-Fei Poon,[a] Alfonso Cabezón,[b] Alessandro Gulotta,[c] Najet Mahmoudi,[d] Stefan Ulvenlund,[e] Rebeca Garcia-Fandiño,[b] Adrian Sanchez-Fernandez.[f]*

[a]European Spallation Source, Box 176, SE-221 00 Lund, Sweden.
[b]Center for Research in Biological Chemistry and Molecular Materials (CIQUS), Department of Organic Chemistry, Universidade de Santiago de Compostela, Rúa de Jenaro de la Fuente, s/n, 15705 Santiago de Compostela, Spain.
[c]Divison of Physical Chemistry, Department of Chemistry, Lund University, Box 124, SE-221 00 Lund, Sweden.
[d]ISIS Neutron and Muon Source, Science & Technology Facilities Council, Rutherford Appleton Laboratory, Chilton OX11 0QX, U.K.
[e]Department of Process and Life Science Engineering, Lund University, Box 117, SE-221 00 Lund, Sweden.
[f]Center for Research in Biological Chemistry and Molecular Materials (CIQUS), Department of Chemical Engineering, University of Santiago de Compostela, Rúa de Jenaro de la Fuente, s/n, 15705 Santiago de Compostela, Spain.
*adriansanchez.fernandez@usc.es

Table of contents

1 Materials 2
2 Synthesis sugar-based surfactants 2
2.1 General 2
2.2 Synthetic procedure 2
2.3 NMR spectra 12
3 Methods 29
4 Data analysis 30
5 Supplementary results 31
5.1 Determination of the solubilisation temperature 32
5.2 Critical micelle concentration determination 32
5.3 DLS correlation curves 33
5.4 Results from SANS/SLS data analysis 33
5.5 MD simulation snapshots 34
5.6 Linear rheology results 34
5.7 Oscillatory rheology results 35
5.8 Cole-Cole plots 35
6 Supporting references 35

1 Materials

Reagents for surfactant synthesis were purchased from commercial suppliers and used without further purification. Aqueous solutions were prepared using deionised water of Mili-Q grade water (resistivity of 18.2 MΩ cm) or D_2O (Sigma-Aldrich, 99.9 atom %D).

2 Synthesis sugar-based surfactants

2.1 General

The precursors *cis*-vaccenic acid, *trans*-vaccenic acid,[1] (Z)-octadec-6-enoic acid,[2] (E)-octadec-6-enoic acid,[3] and octadec-9-ynol[4] were synthesised according to literature procedures. Flash column chromatography was carried out using silica gel (40–63 µm, 230–400 mesh). ^{1}H and ^{13}C NMR were recorded on a Bruker AVANCE spectrometer at 500 and 126 MHz, respectively, unless specified otherwise. High-resolution mass spectra were obtained on a Waters XEVO-G2 QTOF mass spectrometer. No melting point were determined as the sugar-based surfactants formed a glass upon heating.

2.2 Synthetic procedure

To $LiAlH_4$ (1.9 eq.) in anhydrous THF (30 mL) was dropwise added octadec-9-ynoic acid (1.0 eq.) in anhydrous THF (15 mL) under N_2 at 0 °C. After reflux for 3 hours, the reaction mixture was poured to ice-water followed by addition of sat. NH_4Cl solution (50 mL) and extraction with EtOAc (150 mL × 3). The organic phase was washed with brine (150 mL), dried over $MgSO_4$, filtered and evaporated under reduced pressure to afford the title compound.

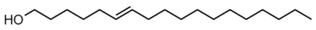

(E)-Octadec-6-enol. To $LiAlH_4$ (760 mg, 20 mmol) in anhydrous THF (25 mL) was dropwise added (E)-octadec-6-enoic acid (3.0 g, 10.5 mmol) in anhydrous THF (13 mL) under N_2 at 0 °C. After reflux for 3 hours, the reaction mixture was poured to ice-water followed by addition of sat. NH_4Cl solution (50 mL) and extraction with EtOAc (150 mL × 3). The organic phase was washed with brine (150 mL), dried over $MgSO_4$, filtered and evaporated under reduced pressure to afford the title compound as pale yellow solid (2.8 g, 98%). ^{1}H NMR was in good agreement with reported data.[5]

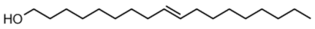

(E)-Octadec-9-enol. To $LiAlH_4$ (3.2 g, 84 mmol) in anhydrous THF (100 mL) was dropwise added (E)-octadec-9-enoic acid (12.6 g, 44.2 mmol) in anhydrous THF (50 mL) under N_2 at 0 °C. After reflux for 3 hours, the reaction mixture was poured to ice-water followed by addition of sat. NH_4Cl solution (50 mL) and extraction with EtOAc (150 mL × 3). The organic phase was washed with brine (150 mL), dried over $MgSO_4$, filtered and evaporated under reduced pressure to afford the title compound as white solid (12 g, quantitative). ^{1}H NMR was in good agreement with reported data.[5]

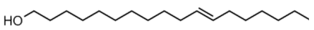

(E)-Octadec-11-enol. To $LiAlH_4$ (1.1 g, 30 mmol) in anhydrous THF (34 mL) was dropwise added (E)-octadec-11-enoic acid (4.3 g, 15.2 mmol) in anhydrous THF (15 mL) under N_2 at 0 °C. After reflux for 3 hours, the reaction mixture was poured to ice-water followed by addition

of sat. NH_4Cl solution (50 mL) and extraction with EtOAc (150 mL × 3). The organic phase was washed with brine (150 mL), dried over $MgSO_4$, filtered and evaporated under reduced pressure to afford the title compound as yellow oil (4.1 g, quantitative). ^{1}H NMR was in good agreement with reported data.[6]

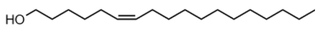

(Z)-Octadec-6-enol. To $LiAlH_4$ (1.4 g, 37 mmol) in anhydrous THF (44 mL) was dropwise added (Z)-octadec-6-enoic acid (5.6 g, 19.8 mmol) in anhydrous THF (22 mL) under N_2 at 0 °C. After reflux for 3 hours, the reaction mixture was poured to ice-water followed by addition of sat. NH_4Cl solution (50 mL) and extraction with EtOAc (150 mL × 3). The organic phase was washed with brine (150 mL), dried over $MgSO_4$, filtered and evaporated under reduced pressure to afford the title compound as pale yellow solid (5.3 g, quantitative). ^{1}H NMR was in good agreement with reported data.[7]

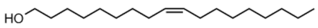

(Z)-Octadec-9-enol. To $LiAlH_4$ (1.6 g, 42 mmol) in anhydrous THF (50 mL) was dropwise added (Z)-octadec-9-enoic acid (6.3 g, 22.1 mmol) in anhydrous THF (25 mL) under N_2 at 0 °C. After reflux for 3 hours, the reaction mixture was poured to ice-water followed by addition of sat. NH_4Cl solution (50 mL) and extraction with EtOAc (150 mL × 3). The organic phase was washed with brine (150 mL), dried over $MgSO_4$, filtered and evaporated under reduced pressure to afford the title compound as pale yellow oil (5.9 g, quantitative). ^{1}H NMR was in good agreement with reported data.[8]

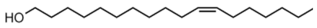

(Z)-Octadec-11-enol. To $LiAlH_4$ (1.4 g, 37 mmol) in anhydrous THF (44 mL) was dropwise added (Z)-octadec-9-enoic acid (5.5 g, 19.5 mmol) in anhydrous THF (22 mL) under N_2 at 0 °C. After reflux for 3 hours, the reaction mixture was poured to ice-water followed by addition of sat. NH_4Cl solution (50 mL) and extraction with EtOAc (150 mL × 3). The organic phase was washed with brine (150 mL), dried over $MgSO_4$, filtered and evaporated under reduced pressure to afford the title compound as pale yellow solid (4.8 g, 92%). ^{1}H NMR was in good agreement with reported data.[9]

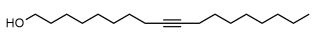

Octadec-9-yn-1-ol. To $LiAlH_4$ (905mg, 24 mmol) in anhydrous THF (30 mL) was dropwise added octadec-9-ynoic acid (3.5 g, 12.5 mmol) in anhydrous THF (15 mL) under N_2 at 0 °C. After reflux for 3 hours, the reaction mixture was poured to ice-water followed by addition of sat. NH_4Cl solution (50 mL) and extraction with EtOAc (150 mL × 3). The organic phase was washed with brine (150 mL), dried over $MgSO_4$, filtered and evaporated under reduced pressure to afford the title compound as pale yellow solid (3.3 g, 99%). ^{1}H NMR was in good agreement with reported data.[9]

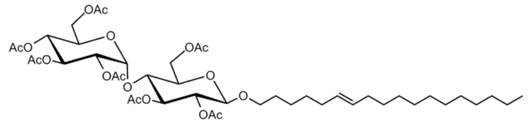

***O*-(*E*-6-octadecenyl) hepta-acetyl-maltose**. To acetobromo-α-maltose (3.5 g, 5 mmol) in anhydrous DCM (27 mL) were added *E*-octadec-6-enol (2.7 g, 10 mmol) and 4Å molecular sieves (1.7 g) under N_2 at r.t. After stirring for 1 hour, Ag_2CO_3 (1.7 g, 6 mmol) and $AgClO_4$ (1.3 g, 6 mmol) were added. The reaction mixture was allowed to stir in dark for another 4 hours in prior to filtration through celite and evaporation under reduced pressure. The residue was purified by column chromatography using heptane/EtOAc (7:3) as eluent to afford the title compound as white solid (630 mg, 14%). ^{1}H NMR (500 MHz, Chloroform-*d*) δ 5.41 (d, *J* = 4.0 Hz, 1H), 5.38 – 5.33 (m, 3H), 5.24 (t, *J* = 9.0 Hz, 1H), 5.04 (t, *J* = 10.0 Hz, 1H), 4.89 – 4.79 (m, 2H), 4.50 (d, *J* = 8.0 Hz, 1H), 4.46 (dd, *J* = 12.0, 3.0 Hz, 1H), 4.23 (ddd, *J* = 12.5, 9.0, 4.0 Hz, 2H), 4.05 – 3.94 (m, 3H), 3.83 (dt, *J* = 9.5, 6.5 Hz, 1H), 3.66 (ddd, *J* = 10.0, 4.5, 2.5 Hz, 1H), 3.45 (dt, *J* = 10.0, 7.0 Hz, 1H), 2.13 (s, 3H), 2.09 (s, 3H), 2.04 (s, 3H), 2.02 – 1.99 (m, 12H), 1.97 – 1.93 (m, 4H), 1.58 – 1.51 (m, 2H), 1.34 – 1.25 (s, 22H), 0.87 (t, *J* = 7.0 Hz, 3H). ^{13}C NMR (126 MHz, Chloroform-d) δ 170.51, 170.47, 170.3, 169.9, 169.6, 169.4, 130.7, 129.8, 100.3, 95.5, 75.5, 72.8, 72.2, 72.0, 70.2, 70.0, 69.3, 68.5, 68.0, 62.9, 61.5, 32.6, 32.5, 31.9, 29.7, 29.6, 29.5, 29.33, 29.28, 29.22, 29.18, 25.5, 25.3, 22.7, 20.9, 20.8, 20.7, 20.60, 20.59, 20.56, 20.55, 14.1. HRMS (ESI): Calc'd for $[Na+M]^+$: 909.4460, Found: 909.4460.

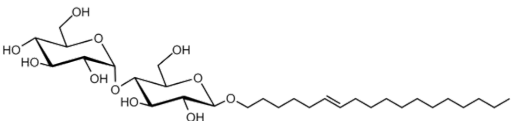

***O*- (*E*-6-octadecenyl)-β-D-maltopyranoside**. To *O*-(*E*-6-octadecenyl) hepta-acetyl-maltose (560 mg, 0.6 mmol) in anhydrous MeOH (12 mL) was added dropwise a solution of NaOMe in MeOH (0.5 M, 1.8 mL, 0.9 mmol) at r.t. under N_2. After stirring for 16 hours, the reaction mixture was neutralised with Amberlite 120 IR Hydrogen-form until pH = 5 – 6, prior to filtration and evaporation under reduced pressure. The residue was purified by column chromatography using heptane/EtOAc (7:3) → DCM/MeOH (85:15) as eluent to afford the title compound as white powder (330 mg, 93%). ^{1}H NMR (500 MHz, Methanol-*d*$_4$) δ 5.40 (m, 2H), 5.16 (d, *J* = 3.5 Hz, 1H), 4.26 (d, *J* = 8.0 Hz, 1H), 3.91 – 3.86 (m, 2H), 3.84 – 3.79 (m, 2H), 3.71 – 3.60 (m, 4H), 3.55 – 3.51 (m, 2H), 3.44 (dd, *J* = 9.5, 4.0 Hz, 1H), 3.38 – 3.34 (m, 1H), 3.28 – 3.21 (m, 2H), 2.05 – 1.96 (m, 4H), 1.62 (m, 2H), 1.40 – 1.29 (m, 22H), 0.90 (t, *J* = 7.0 Hz, 3H). ^{13}C NMR (126 MHz, Methanol-*d*$_4$) δ 131.6, 131.4, 104.32, 102.9, 81.3, 77.9, 76.6, 75.1, 74.8, 74.7, 74.2, 71.2, 70.9, 62.8, 62.2, 33.63, 33.60, 33.1, 30.77, 30.75, 30.7, 30.64, 30.59, 30.5, 30.2, 26.6, 23.7, 14.5. HRMS (ESI): Calc'd for $[Na+M]^+$: 615.3720, Found: 615.3727.

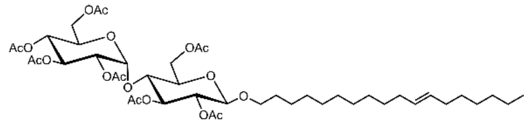

***O*-(*E*-11-octadecenyl) hepta-acetyl-maltose**. To acetobromo-α-maltose (4.2g, 6 mmol) in anhydrous DCM (32 mL) were added trans-vaccenyl alcohol (3.2 g, 12 mmol) and 4Å molecular sieves (2.0 g) under N_2 at r.t. After stirring for 1 hour, Ag_2CO_3 (2.0 g, 7.2 mmol) and $AgClO_4$ (1.5 g, 7.2 mmol) were added. The reaction mixture was allowed to stir in dark for another 4 hours in prior to filtration through celite and evaporation under reduced pressure. The residue was purified by column chromatography using heptane/EtOAc (7:3) as eluent to

afford the title compound as white solid (850 mg, 16%). ^{1}H NMR (500 MHz, Chloroform-*d*) δ 5.40 (d, *J* = 4.0 Hz, 1H), 5.39 – 5.32 (m, 3H), 5.24 (t, *J* = 9.2 Hz, 1H), 5.04 (t, *J* = 9.9 Hz, 1H), 4.88 – 4.77 (m, 2H), 4.50 (d, *J* = 7.9 Hz, 1H), 4.46 (dd, *J* = 12.1, 2.8 Hz, 1H), 4.23 (ddd, *J* = 12.4, 8.7, 4.2 Hz, 2H), 4.07 – 3.92 (m, 3H), 3.83 (dt, *J* = 9.6, 6.4 Hz, 1H), 3.66 (ddd, *J* = 9.7, 4.4, 2.9 Hz, 1H), 3.45 (dt, *J* = 9.6, 6.8 Hz, 1H), 2.13 (s, 3H), 2.09 (s, 3H), 2.06 – 1.98 (m, 15H), 1.98 – 1.91 (m, 4H), 1.54 (h, *J* = 6.7, 6.2 Hz, 2H), 1.33 – 1.22 (m, 22H), 0.87 (t, *J* = 6.9 Hz, 3H). ^{13}C NMR (126 MHz, Chloroform-*d*) δ 170.5, 170.5, 170.3, 169.9, 169.6, 169.4, 130.4, 130.3, 100.3, 95.5, 75.5, 72.8, 72.2, 72.0, 70.2, 70.0, 69.3, 68.4, 68.0, 62.9, 61.5, 32.6, 31.7, 29.62, 29.59, 29.57, 29.54, 29.48, 29.4, 29.3, 29.1, 28.8, 25.8, 22.6, 20.9, 20.8, 20.7, 20.59, 20.58, 20.54, 14.1. HRMS (ESI): Calc'd for $[Na+M]^+$: 909.4460, Found: 909.4473.

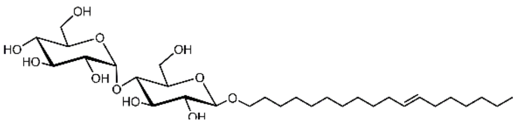

***O*-(*E*-11-octadecenyl)-β-D-maltopyranoside**. To *O*-(*E*-11-octadecenyl) hepta-acetyl-maltose (780 mg, 0.9 mmol) in anhydrous MeOH (16 mL) was added dropwise a solution of NaOMe in MeOH (0.5 M, 2.5 mL, 1.3 mmol) at r.t. under N_2. After stirring for 16 hours, the reaction mixture was neutralised with Amberlite 120 IR Hydrogen-form until pH = 5 – 6, prior to filtration and evaporation under reduced pressure. The residue was purified by column chromatography using heptane/EtOAc (7:3) → DCM/MeOH (9:1) as eluent to afford the title compound as white powder (480 mg, 92%). ^{1}H NMR (500 MHz, Methanol-d_4) δ 5.39 – 5.37 (m, 2H), 5.16 (d, *J* = 3.8 Hz, 1H), 4.26 (d, *J* = 7.8 Hz, 1H), 3.93 – 3.85 (m, 2H), 3.85 – 3.77 (m, 2H), 3.72 – 3.57 (m, 4H), 3.57 – 3.49 (m, 2H), 3.44 (dd, *J* = 9.7, 3.8 Hz, 1H), 3.36 (ddd, *J* = 9.6, 4.6, 2.1 Hz, 1H), 3.29 – 3.19 (m, 2H), 1.98 (q, *J* = 6.2 Hz, 4H), 1.67 – 1.57 (m, 2H), 1.39 – 1.28 (m, 20H), 0.90 (t, *J* = 7.0 Hz, 3H). ^{13}C NMR (126 MHz, Methanol-d_4) δ 131.5, 104.3, 102.9, 81.3, 77.9, 76.6, 75.1, 74.8, 74.7, 74.2, 71.5, 71.0, 62.8, 62.2, 33.6, 32.9, 30.81, 30.77, 30.7, 30.6, 30.6, 30.2, 29.9, 27.1, 23.7, 14.4. Calc'd for $[Na+M]^+$: 615.3720, Found: 615.3720.

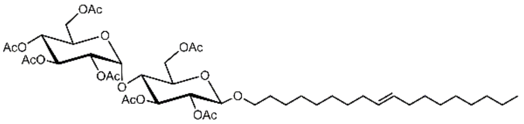

***O*-(*E*-9-octadecenyl) hepta-acetyl-maltose**. To acetobromo-α-maltose (4.2g, 6 mmol) in anhydrous DCM (32 mL) were added trans-vaccenyl alcohol (3.2 g, 12 mmol) and 4Å molecular sieves (2.0 g) under N_2 at r.t. After stirring for 1 hour, Ag_2CO_3 (2.0 g, 7.2 mmol) and $AgClO_4$ (1.5 g, 7.2 mmol) were added. The reaction mixture was allowed to stir in dark for another 4 hours in prior to filtration through celite and evaporation under reduced pressure. The residue was purified by column chromatography using heptane/EtOAc (7:3) as eluent to afford the title compound as white solid (1.1 g, 20%). ^{1}H NMR (500 MHz, Chloroform-*d*) δ 5.40 (d, *J* = 4.0 Hz, 1H), 5.39 – 5.31 (m, 3H), 5.24 (t, *J* = 9.2 Hz, 1H), 5.04 (t, *J* = 9.8 Hz, 1H), 4.88 – 4.77 (m, 2H), 4.50 (d, *J* = 7.9 Hz, 1H), 4.46 (dd, *J* = 12.1, 2.8 Hz, 1H), 4.23 (ddd, *J* = 12.4, 8.8, 4.2 Hz, 2H), 4.06 – 3.92 (m, 3H), 3.83 (dt, *J* = 9.7, 6.4 Hz, 1H), 3.66 (ddd, *J* = 9.7, 4.4, 2.8 Hz, 1H), 3.45 (dt, *J* = 9.6, 6.8 Hz, 1H), 2.13 (s, 3H), 2.09 (s, 3H), 2.03 (s, 3H), 2.02 – 1.98 (m, 11H), 1.97 – 1.92 (m, 4H), 1.53 (p, *J* = 7.1, 6.6 Hz, 2H), 1.25 (s, 22H), 0.87 (t, *J* = 6.9 Hz, 3H). ^{13}C NMR (126 MHz, Chloroform-*d*) δ 170.52, 170.47, 170.3, 169.9, 169.6, 169.4, 130.4, 130.2, 100.3, 95.5, 75.5, 72.8, 72.2, 72.0, 70.2, 70.0, 69.3, 68.4, 68.0, 62.9, 61.5, 32.57, 32.55, 31.9,

29.62, 29.61, 29.5, 29.44, 29.35, 29.28, 29.26, 29.2, 29.1, 25.8, 22.7, 20.9, 20.8, 20.7, 20.60, 20.58, 20.55, 14.1. Calc'd for $[Na+M]^+$: 909.4460, Found: 909.4461.

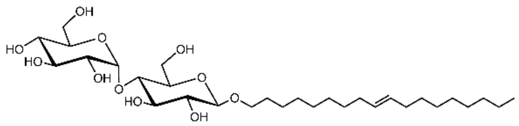

***O*-(*E*-9-octadecenyl)-β-D-maltopyranoside**. To *O*-(*E*-9-octadecenyl) hepta-acetyl-maltose (920 mg, 1.0 mmol) in anhydrous MeOH (18 mL) was added dropwise a solution of NaOMe in MeOH (0.5 M, 2.9 mL, 1.5 mmol) at r.t. under N_2. After stirring for 16 hours, the reaction mixture was neutralised with Amberlite 120 IR Hydrogen-form until pH = 5 – 6, filtered and evaporated under reduced pressure. The residue was purified by column chromatography using heptane/EtOAc (7:3) → DCM/MeOH (9:1) as eluent to afford the title compound as white powder (600 mg, 97%). ^{1}H NMR (500 MHz, Methanol-d_4) δ 5.38 (td, *J* = 3.8, 1.9 Hz, 2H), 5.16 (d, *J* = 3.8 Hz, 1H), 4.26 (d, *J* = 7.8 Hz, 1H), 3.93 – 3.86 (m, 2H), 3.86 – 3.77 (m, 2H), 3.72 – 3.57 (m, 4H), 3.57 – 3.49 (m, 2H), 3.44 (dd, *J* = 9.7, 3.8 Hz, 1H), 3.36 (ddd, *J* = 9.6, 4.6, 2.1 Hz, 1H), 3.30 – 3.19 (m, 2H), 2.01 – 1.94 (m, 4H), 1.67 – 1.57 (m, 2H), 1.42 – 1.24 (m, 22H), 0.90 (t, *J* = 6.9 Hz, 3H). ^{13}C NMR (126 MHz, Methanol-d_4) δ 131.5, 104.3, 102.9, 81.3, 77.9, 76.6, 75.1, 74.8, 74.7, 74.2, 71.5, 71.0, 62.8, 62.2, 33.62, 33.61, 33.0, 30.78, 30.76, 30.62, 30.59, 30.57, 30.5, 30.2, 27.1, 23.7, 14.5. Calc'd for $[Na+M]^+$: 615.3720, Found: 615.3718.

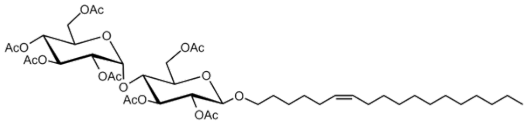

***O*-(*Z*-6-octadecenyl) hepta-acetyl-maltose**. To acetobromo-α-maltose (4.2 g, 6 mmol) in anhydrous DCM (32 mL) were added *Z*-octadec-6-enol (3.2 g, 12 mmol) and 4Å molecular sieves (2 g) under N_2 at r.t. After stirring for 1 hour, Ag_2CO_3 (2 g, 7.2 mmol) and $AgClO_4$ (1.5 g, 7.2 mmol) were added. The reaction mixture was allowed to stir in dark for another 4 hours in prior to filtration through celite and evaporation under reduced pressure. The residue was purified by column chromatography using heptane/EtOAc (7:3) as eluent to afford the title compound as white solid (940 mg, 18%). ^{1}H NMR (500 MHz, Chloroform-*d*) δ 5.41 (d, *J* = 4.0 Hz, 1H), 5.37 – 5.30 (m, 3H), 5.24 (t, *J* = 9.5 Hz, 1H), 5.04 (t, *J* = 10.0 Hz, 1H), 4.87 – 4.79 (m, 2H), 4.50 (d, *J* = 8.0 Hz, 1H), 4.46 (dd, *J* = 12.0, 3.0 Hz, 1H), 4.24 (ddd, *J* = 12.0, 9.5, 4.0 Hz, 2H), 4.05 – 3.94 (m, 3H), 3.84 (dt, *J* = 9.5, 6.5 Hz, 1H), 3.68 – 3.65 (m, 1H), 3.46 (dt, *J* = 9.5, 7.0 Hz, 1H), 2.13 (s, 3H), 2.10 (s, 3H), 2.04 (s, 3H), 2.02 – 2.00 (m, 12H), 1.58 – 1.52 (m, 2H), 1.37 – 1.25 (s, 22H), 0.87 (t, *J* = 7.0 Hz, 3H). ^{13}C NMR (126 MHz, $CDCl_3$) δ 170.52, 170.47, 170.3, 169.9, 169.6, 169.4, 130.2, 129.4, 100.3, 95.5, 75.5, 72.8, 72.2, 72.0, 70.1, 70.0, 69.3, 68.5, 68.0, 62.9, 61.5, 31.9, 29.7, 29.7, 29.62, 29.55, 29.4, 29.33, 29.31, 29.28, 27.2, 27.1, 25.5, 22.7, 20.9, 20.8, 20.7, 20.61, 20.59, 20.56, 20.55, 14.1. Calc'd for $[Na+M]^+$: 909.4460, Found: 909.4468.

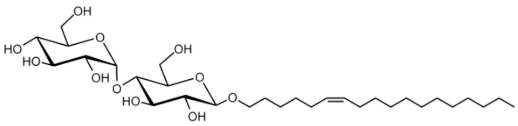

(*Z*-6-octadecenyl)-β-D-maltopyranoside. To *O*-(*Z*-6-octadecenyl)-hepta-acetyl-maltose (850 mg, 1.0 mmol) in anhydrous MeOH (17 mL) was added dropwise a solution of NaOMe in MeOH (0.5 M, 2.7 mL, 1.4 mmol) at r.t. under N_2. After stirring for 16 hours, the reaction mixture was neutralised with Amberlite 120 IR Hydrogen-form until pH = 5 – 6, filtered and evaporated under reduced pressure. The residue was purified by column chromatography using heptane/EtOAc (7:3) → DCM/MeOH (9:1) as eluent to afford the title compound as white powder (560 mg, 98%). ^{1}H NMR (500 MHz, Methanol-d_4) δ 5.40 – 5.32 (m, 2H), 5.16 (d, J = 4.0 Hz, 1H), 4.27 (d, J = 8.0 Hz, 1H), 3.91 – 3.86 (m, 2H), 3.85 – 3.79 (m, 2H), 3.70 – 3.59 (m, 4H), 3.56 – 3.51 (m, 2H), 3.44 (dd, J = 9.5, 3.5 Hz, 1H), 3.36 (ddd, J = 9.5, 4.5, 2.0 Hz, 1H), 3.29 – 3.21 (m, 2H), 2.07 – 2.01 (m, 4H), 1.66 – 1.60 (m, 2H), 1.41 – 1.29 (m, 22H), 0.90 (t, J = 7.0 Hz, 3H). ^{13}C NMR (126 MHz, Methanol-d_4) δ 130.9, 130.7, 104.3, 102.9, 81.3, 77.9, 76.6, 75.1, 74.8, 74.7, 74.2, 71.5, 70.9, 62.8, 62.2, 33.1, 30.84, 30.77, 30.75, 30.71, 30.6, 30.5, 30.3, 28.2, 28.1, 26.8, 23.7, 14.5. Calc'd for $[Na+M]^+$: 615.3720, Found: 615.3716.

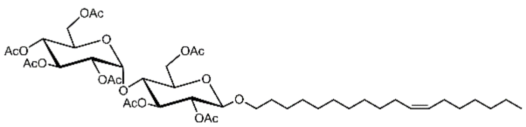

O-(*Z*-11-octadecenyl) hepta-acetyl-maltose. To acetobromo-α-maltose (4.7g, 6.8 mmol) in anhydrous DCM (36 mL) were added *cis*-vaccenyl alcohol (3.6 g, 13.5 mmol) and 4Å molecular sieves (2.3 g) under N_2 at r.t. After stirring for 1 hour, Ag_2CO_3 (2.2 g, 8.1 mmol) and $AgClO_4$ (1.7 g, 8.1 mmol) were added. The reaction mixture was allowed to stir in dark for another 4 hours in prior to filtration through celite and evaporation under reduced pressure. The residue was purified by column chromatography using heptane/EtOAc (7:3) as eluent to afford the title compound as white solid (1.1 g, 19%). ^{1}H NMR (500 MHz, Chloroform-d) δ 5.41 (d, J = 4.0 Hz, 1H), 5.38 – 5.32 (m, 3H), 5.24 (t, J = 9.2 Hz, 1H), 5.04 (t, J = 9.9 Hz, 1H), 4.89 – 4.77 (m, 2H), 4.50 (d, J = 7.9 Hz, 1H), 4.46 (dd, J = 12.1, 2.8 Hz, 1H), 4.24 (ddd, J = 12.4, 8.7, 4.2 Hz, 2H), 4.07 – 3.92 (m, 3H), 3.83 (dt, J = 9.6, 6.4 Hz, 1H), 3.67 (ddd, J = 9.6, 4.4, 2.8 Hz, 1H), 3.46 (dt, J = 9.6, 6.8 Hz, 1H), 2.13 (s, 3H), 2.10 (s, 3H), 2.07 – 1.91 (m, 19H), 1.53 (p, J = 7.2, 6.7 Hz, 2H), 1.36 – 1.21 (m, 22H), 0.88 (m, J = 7.0 Hz, 3H). ^{13}C NMR (126 MHz, Chloroform-d) δ 170.53, 170.48, 170.3, 170.0, 169.6, 169.4, 130.0, 129.8, 100.3, 95.5, 75.5, 72.8, 72.2, 72.0, 70.2, 70.0, 69.3, 68.5, 68.0, 62.9, 61.5, 31.8, 29.8, 29.7, 29.6, 29.6, 29.5, 29.4, 29.3, 29.3, 29.0, 27.2, 27.2, 25.8, 22.6, 20.9, 20.8, 20.7, 20.60, 20.59, 20.56, 20.55, 14.1. Calc'd for $[Na+M]^+$: 909.4460, Found: 909.4464.

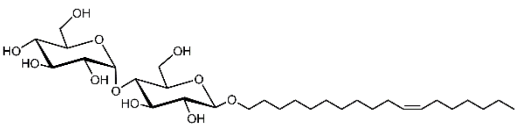

O-(*Z*-11-octadecenyl)-β-D-maltopyranoside. To *O*-(*Z*-11-octadecenyl)-hepta-acetyl-maltose (750 mg, 0.85 mmol) in anhydrous MeOH (15 mL) was added dropwise a solution of NaOMe in MeOH (0.5 M, 2.4 mL, 1.2 mmol) at r.t. under N_2. After stirring for 16 hours, the reaction mixture was neutralised with Amberlite 120 IR Hydrogen-form until pH = 5 – 6, filtered and evaporated under reduced pressure. The residue was purified by column chromatography using heptane/EtOAc (7:3) → DCM/MeOH (9:1) as eluent to afford the title compound as white powder (488 mg, 97%). ^{1}H NMR (500 MHz, Methanol-d_4) δ 5.41 – 5.30 (m, 2H), 5.16 (d, J = 3.8 Hz, 1H), 4.26 (d, J = 7.8 Hz, 1H), 3.93 – 3.85 (m, 2H), 3.85 – 3.77 (m, 2H), 3.72 – 3.57 (m,

4H), 3.57 – 3.49 (m, 2H), 3.44 (dd, *J* = 9.7, 3.8 Hz, 1H), 3.39 – 3.32 (m, 1H), 3.31 – 3.19 (m, 3H), 2.07 – 1.94 (m, 4H), 1.67 – 1.57 (m, 2H), 1.44 – 1.22 (m, 22H), 0.91 (m, *J* = 7.0 Hz, 3H). ^{13}C NMR (126 MHz, Methanol-*d*$_4$) δ 130.8, 104.3, 102.9, 81.3, 77.9, 76.6, 75.1, 74.8, 74.7, 74.2, 71.5, 71.0, 62.8, 62.2, 32.9, 30.84, 30.83, 30.80, 30.75, 30.7, 30.6, 30.3, 30.0, 28.14, 28.12, 27.1, 23.7, 14.5. Calc'd for $[Na+M]^+$: 615.3720, Found: 615.3713.

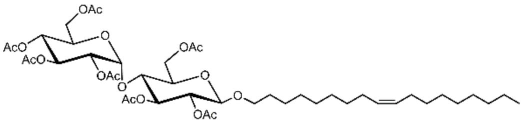

O-(Z-9-octadecenyl)-hepta-acetyl-maltose. To acetobromo-α-maltose (4.2g, 6 mmol) in anhydrous DCM (36 mL) were added oleyl alcohol (3.2 g, 12 mmol) and 4Å molecular sieves (2.0 g) under N_2 at r.t. After stirring for 1 hour, Ag_2CO_3 (2.0 g, 7.2 mmol) and $AgClO_4$ (1.5 g, 7.2 mmol) were added. The reaction mixture was allowed to stir in dark for another 4 hours in prior to filtration through celite and evaporation under reduced pressure. The residue was purified by column chromatography using heptane/EtOAc (7:3) as eluent to afford the title compound as white solid (R_f = 0.18, 1.1 g, 20%). 1H NMR (500 MHz, Chloroform-d) δ 5.41 (d, J = 4.0 Hz, 1H), 5.38 – 5.30 (m, 3H), 5.24 (t, J = 9.1 Hz, 1H), 5.04 (t, J = 9.9 Hz, 1H), 4.89 – 4.77 (m, 2H), 4.50 (d, J = 7.9 Hz, 1H), 4.46 (dd, J = 12.1, 2.8 Hz, 1H), 4.24 (ddd, J = 12.4, 8.7, 4.2 Hz, 2H), 4.07 – 3.92 (m, 3H), 3.83 (dt, J = 9.6, 6.4 Hz, 1H), 3.70 – 3.62 (m, 1H), 3.46 (dt, J = 9.6, 6.8 Hz, 1H), 2.14 (s, 3H), 2.10 (s, 3H), 2.07 – 1.93 (m, 19H), 1.54 (dt, J = 13.8, 6.8 Hz, 2H), 1.35 – 1.24 (m, 22H), 0.87 (t, J = 6.9 Hz, 3H). 13C NMR (126 MHz, Chloroform-d) δ 170.5, 170.3, 170.0, 169.6, 169.4, 130.0, 129.8, 100.3, 95.5, 75.5, 72.8, 72.2, 72.0, 70.2, 70.0, 69.4, 68.5, 68.0, 62.9, 61.5, 31.9, 29.7, 29.5, 29.4, 29.29, 29.28, 29.2, 27.19, 27.18, 25.8, 22.7, 20.9, 20.8, 20.7, 20.61, 20.59, 20.56, 14.1. Calc'd for $[Na+M]^+$: 909.4460, Found: 909.4464.

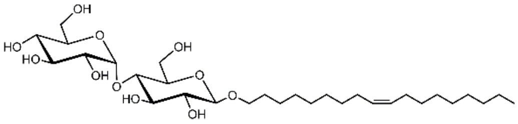

(*Z*-9-octadecenyl)-β-D-maltopyranoside. To *O*-(*Z*-9-octadecenyl)-hepta-acetyl-maltose (850 mg, 1.0 mmol) in anhydrous MeOH (17 mL) was added dropwise a solution of NaOMe in MeOH (0.5 M, 2.7 mL, 1.4 mmol) at r.t. under N_2. After stirring for 16 hours, the reaction mixture was neutralised with Amberlite 120 IR Hydrogen-form until pH = 5 – 6, filtered and evaporated under reduced pressure. The residue was purified by column chromatography using heptane/EtOAc (7:3) → DCM/MeOH (9:1) as eluent to afford the title compound as white powder (560 mg, 98%). ^{1}H NMR (500 MHz, Methanol-*d*$_4$) δ 5.34 (td, *J* = 4.8, 4.0, 1.2 Hz, 2H), 5.16 (d, *J* = 3.8 Hz, 1H), 4.26 (d, *J* = 7.8 Hz, 1H), 3.93 – 3.86 (m, 2H), 3.86 – 3.78 (m, 2H), 3.72 – 3.48 (m, 6H), 3.44 (dd, *J* = 9.7, 3.8 Hz, 1H), 3.36 (ddd, *J* = 9.6, 4.6, 2.1 Hz, 1H), 3.29 – 3.20 (m, 2H), 2.03 (q, *J* = 6.4 Hz, 4H), 1.67 – 1.57 (m, 2H), 1.41 – 1.25 (m, 22H), 0.90 (t, *J* = 7.0 Hz, 3H). ^{13}C NMR (126 MHz, Methanol-*d*$_4$) δ 130.8, 104.3, 102.9, 81.3, 77.9, 76.6, 75.1, 74.8, 74.7, 74.2, 71.5, 71.0, 62.8, 62.2, 33.1, 30.9, 30.83, 30.80, 30.64, 30.62, 30.60, 30.4, 30.34, 30.33, 28.2, 28.1, 27.1, 23.7, 14.5. Calc'd for $[Na+M]^+$: 615.3720, Found: 615.3727.

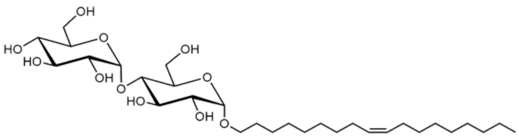

(*Z*-9-octadecenyl)-α-D-maltopyranoside. To acetobromo-α-maltose (4.2g, 6 mmol) in anhydrous DCM (36 mL) were added oleyl alcohol (3.2 g, 12 mmol) and 4Å molecular sieves (2.0 g) under N_2 at r.t. After stirring for 1 hour, Ag_2CO_3 (2.0 g, 7.2 mmol) and $AgClO_4$ (1.5 g, 7.2 mmol) were added. The reaction mixture was allowed to stir in dark for another 4 hours in prior to filtration through celite and evaporation under reduced pressure. The residue was purified by column chromatography using heptane/EtOAc (7:3 → 5:5) as eluent. Fractions with R_f = 0.09 (heptane/EtOAc = 7:3) were collected and condensed under reduced pressure. The white waxy solid (700 mg) was re-dissolved in anhydrous MeOH (14 mL) in prior to addition of NaOMe in MeOH (0.5 M, 2.2 mL, 1.1 mmol) at r.t. under N_2. After stirring for 24 hours, the reaction mixture was neutralised with Amberlite 120 IR Hydrogen-form until pH = 5 – 6, filtered and evaporated under reduced pressure. The residue was purified by column chromatography using heptane/EtOAc (7:3) → DCM/MeOH (9:1) as eluent to afford the title compound as white powder (405 mg, 11% over 2 steps). ^{1}H NMR (500 MHz, Methanol-d_4) δ 5.36 – 5.34 (m, 2H), 5.14 (d, J = 3.5 Hz, 1H), 4.76 (d, J = 4.0 Hz, 1H), 3.90 – 3.86 (m, 1H), 3.85 – 3.81 (m, 3H), 3.73 – 3.61 (m, 5H), 3.53 – 3.49 (m, 1H), 3.47– 3.42 (m, 3H), 3.27 (t, J = 9.5 Hz, 1H), 2.03 (q, J = 6.0 Hz, 4H), 1.67 – 1.59 (m, 2H), 1.41 – 1.29 (m, 22H), 0.90 (t, J = 7.0 Hz, 3H). ^{13}C NMR (126 MHz, Methanol-d_4) δ 130.8, 103.0, 100.0, 81.8, 75.1, 74.9, 74.8, 74.3, 73.2, 72.3, 71.5, 69.3, 62.7, 62.1, 33.1, 30.9, 30.84, 30.8, 30.64, 30.6, 30.45, 30.4, 30.3, 28.2, 28.1, 27.3, 23.7, 14.5. Calc'd for $[Na+M]^+$: 615.3720, Found: 615.3704.

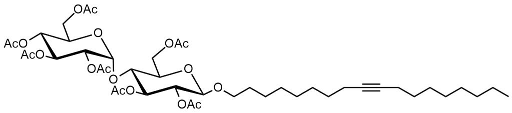

***O*-(9-octadecynyl)-hepta-acetyl-maltose.** To acetobromo-α-maltose (4.1g, 5.8 mmol) in anhydrous DCM (32 mL) were added octadec-9-yn-1-ol (3.1 g, 11.6 mmol) and 4Å molecular sieves (2 g) under N_2 at r.t. After stirring for 1 hour, Ag_2CO_3 (2.2 g, 8.1 mmol) and $AgClO_4$ (1.7 g, 8.1 mmol) were added. The reaction mixture was allowed to stir in dark for another 3 hours in prior to filtration through celite and evaporation under reduced pressure. The residue was purified by column chromatography using heptane/EtOAc (7:3) as eluent to afford the title compound as white solid (840 mg, 16%). ^{1}H NMR (500 MHz, Chloroform-d) δ 5.41 (d, J = 4.0 Hz, 1H), 5.35 (t, J = 10.0 Hz, 1H), 5.24 (t, J = 9.5 Hz, 1H), 5.04 (t, J = 10.0 Hz, 1H), 4.87 – 4.79 (m, 2H), 4.50 (d, J = 8.0 Hz, 1H), 4.46 (dd, J = 12.0, 3,0 Hz, 1H), 4.24 (ddd, J = 12.0, 9.0, 4.0 Hz, 2H), 4.05 – 3.94 (m, 3H), 3.83 (dt, J = 10.0, 6.0 Hz, 1H), 3.70 – 3.62 (m, 2H), 3.45 (dt, J = 10.0, 6.5 Hz, 1H), 2.14 – 2.10 (m, 10H), 2.04 – 2.00 (m, 15H), 1.58 – 1.52 (m, 2H), 1.49 – 1.43 (m, 4H), 1.38 – 1.25 (m, 18H), 0.87 (t, J = 7.0 Hz, 3H). ^{13}C NMR (126 MHz, $CDCl_3$) δ 170.52, 170.47, 170.3, 169.9, 169.6, 169.4, 100.3, 95.5, 80.3, 80.1, 75.5, 72.8, 72.2, 72.0, 70.2, 70.0, 69.3, 68.5, 68.0, 63.0, 62.9, 61.5, 32.8, 31.8, 29.4, 29.3, 29.20, 29.15, 29.13, 29.10, 28.9, 28.78, 28.76, 25.8, 25.7, 22.6, 20.9, 20.8, 20.7, 20.60, 20.59, 20.55, 18.7, 14.1. Calc'd for $[Na+M]^+$: 907.4307, Found: 907.4316.

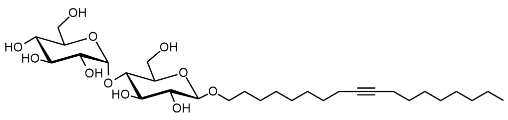

(*9*-octadecynyl)-β-D-maltopyranoside. To *O*-(9-octadecynyl)-hepta-acetyl-maltose (730 mg, 0.8 mmol) in anhydrous MeOH (15 mL) was added dropwise a solution of NaOMe in MeOH (0.5 M, 2.3 mL, 1.2 mmol) at r.t. under N_2. After stirring for 16 hours, the reaction mixture was neutralised with Amberlite 120 IR Hydrogen-form until pH = 5 – 6, filtered and evaporated under reduced pressure. The residue was purified by column chromatography using heptane/EtOAc (7:3) → DCM/MeOH (85:15) as eluent to afford the title compound as white powder (450 mg, 95%). ^{1}H NMR (500 MHz, Methanol-d_4) δ 5.16 (d, J = 4.0 Hz, 1H), 4.27 (d, J = 8.0 Hz, 1H), 3.91 – 3.87 (m, 2H), 3.84 – 3.79 (m, 2H), 3.70 – 3.59 (m, 4H), 3.56 – 3.51 (m, 2H), 3.44 (dd, J = 10.0, 4.0 Hz, 1H), 3.36 (ddd, J = 10.0, 4.5, 2.5 Hz, 1H), 3.28 – 3.20 (m, 2H), 2.13 – 2.10 (m, 4H), 1.65 – 1.60 (m, 2H), 1.49 – 1.29 (m, 22H), 0.91 (t, J = 7.0 Hz, 3H). ^{13}C NMR (126 MHz, MeOD) δ 104.3, 102.9, 81.3, 80.9, 77.9, 76.6, 75.1, 74.8, 74.7, 74.2, 71.5, 70.9, 62.8, 62.2, 33.0, 30.8, 30.7, 30.4, 30.28, 30.25, 30.24, 30.22, 29.9, 29.8, 27.1, 23.7, 19.4, 14.5. Calc'd for $[Na+M]^+$: 613.3564, Found: 613.3563.

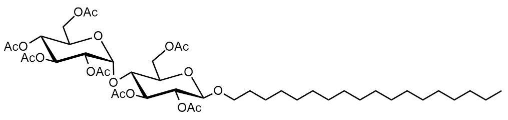

***O*-(octadecyl)-hepta-acetyl-maltose.** To acetobromo-α-maltose (4.2g, 6.0 mmol) in anhydrous DCM (32 mL) were added stearyl alcohol (3.3 g, 12 mmol) and 4Å molecular sieves (2 g) under N_2 at r.t. After stirring for 1 hour, Ag_2CO_3 (2.0 g, 7.2 mmol) and $AgClO_4$ (1.5 g, 7.2 mmol) were added. The reaction mixture was allowed to stir in dark for another 3 hours in prior to filtration through celite and evaporation under reduced pressure. The residue was purified by column chromatography using heptane/EtOAc (7:3) as eluent to afford the title compound as white solid (840 mg, 16%). ^{1}H NMR (500 MHz, Chloroform-d) δ 5.41 (d, J = 4.0 Hz, 1H), 5.35 (dd, J = 10.5, 9.5 Hz, 1H), 5.24 (t, J = 9.0 Hz, 1H), 5.04 (t, J = 10.0 Hz, 1H), 4.87 – 4.79 (m, 2H), 4.50 (d, J = 8.0 Hz, 1H), 4.46 (dd, J = 12.0, 3.0 Hz, 1H), 4.24 (ddd, J = 12.5, 9.0, 4.5 Hz, 2H), 4.05 – 3.94 (m, 3H), 3.83 (dt, J = 9.5, 6.5 Hz, 1H), 3.66 (ddd, J = 9.5, 4.0, 2.5 Hz, 1H), 3.46 (dt, J = 10.0, 6.5 Hz, 1H), 2.13 (s, 3H), 2.10 (s, 3H), 2.04 – 2.00 (m, 15H), 1.56 – 1.52 (m, J = 2H), 1.30 – 1.24 (m, 30H), 0.87 (t, J = 6.9 Hz, 3H). ^{13}C NMR (126 MHz, $CDCl_3$) δ 170.52, 170.47, 170.3, 169.9, 169.6, 169.4, 100.3, 95.5, 75.45, 72.8, 72.2, 72.0, 70.2, 70.0, 69.3, 68.5, 68.0, 62.9, 61.5, 31.9, 29.67, 29.66, 29.63, 29.60, 29.4, 29.3, 29.3, 25.8, 22.7, 20.9, 20.8, 20.7, 20.61, 20.59, 20.56, 20.55, 14.1.

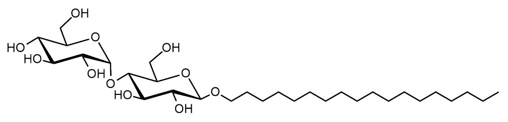

***O*-octadecyl-β-D-maltopyranoside**. To *O*-(octadecanyl)-hepta-acetyl-maltose (740 mg, 0.8 mmol) in anhydrous MeOH (45 mL) was added dropwise a solution of NaOMe in MeOH (0.5 M, 2.3 mL, 1.2 mmol) at r.t. under N_2. After stirring for 16 hours, the reaction mixture was neutralised with Amberlite 120 IR Hydrogen-form until pH = 5 – 6, filtered and evaporated under reduced pressure. The residue was purified by column chromatography using heptane/EtOAc (7:3) → DCM/MeOH (9:1) as eluent to afford the title compound as white

powder (450 mg, 91%). ^{1}H NMR (500 MHz, Methanol-d_4) δ 5.16 (d, J = 4.0 Hz, 1H), 4.26 (d, J = 7.5 Hz, 1H), 3.91 – 3.87 (m, 2H), 3.84 – 3.79 (m, 2H), 3.70 – 3.60 (m, 5H), 3.56 – 3.51 (m, 2H), 3.44 (dd, J = 9.5, 4.0 Hz, 1H), 3.36 (ddd, J = 9.5, 4.5, 2.0 Hz, 1H), 3.28 – 3.20 (m, 2H), 1.65 – 1.59 (m, 2H), 1.41 – 1.29 (s, 30H), 0.90 (t, J = 7.0 Hz, 3H). ^{13}C NMR (126 MHz, MeOD) δ 104.3, 102.9, 81.4, 77.9, 76.6, 75.1, 74.8, 74.7, 74.2, 71.5, 71.0, 62.8, 62.2, 33.1, 30.78, 30.76, 30.6, 30.5, 27.1, 23.7, 14.4.

2.3 NMR spectra

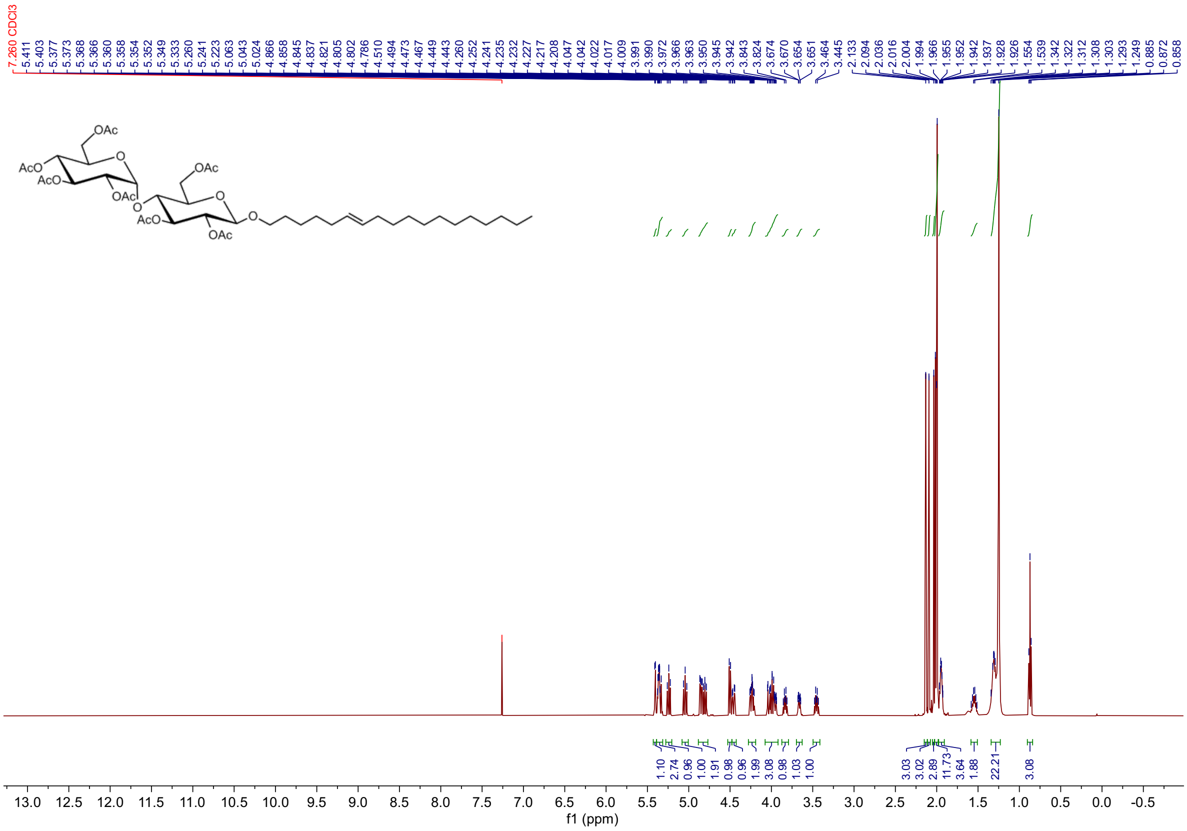

Fig. S1. ^{1}H NMR spectrum of *O*-(*E*-6-octadecenyl) hepta-acetyl-maltose.

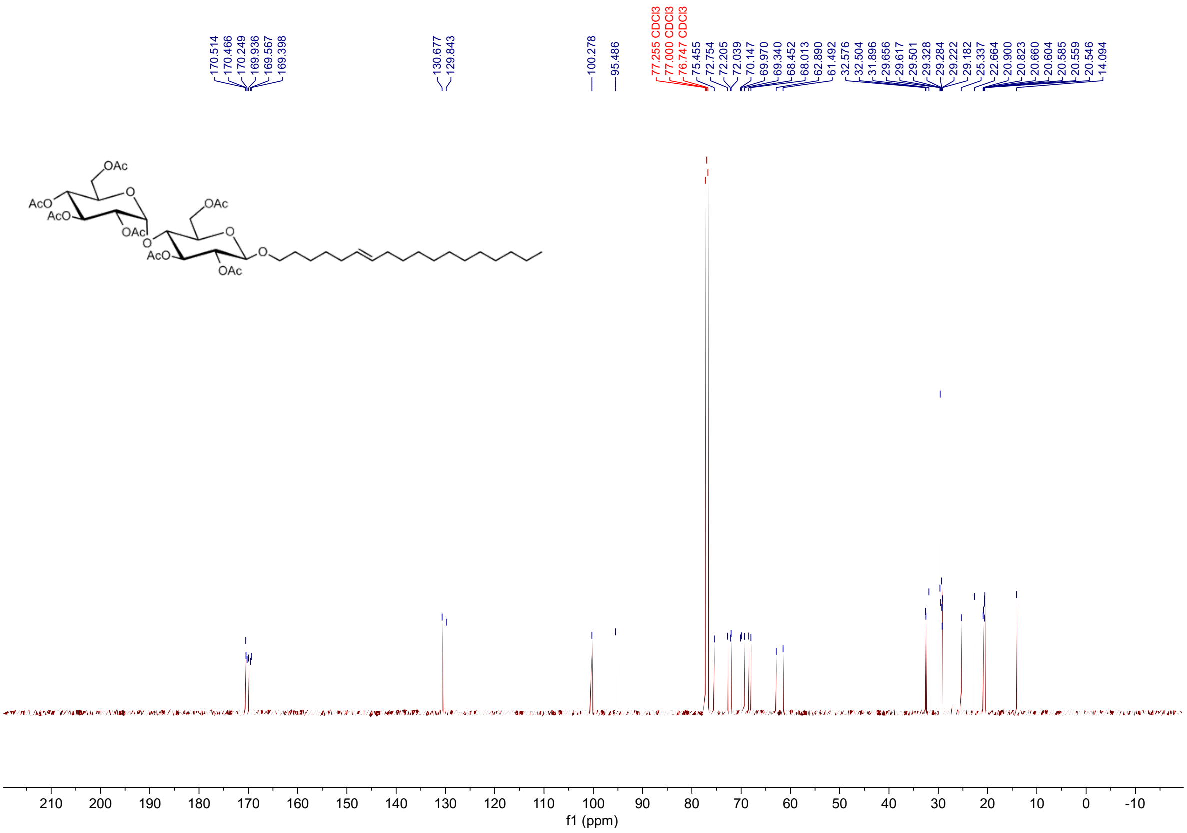

Fig. S2. ^{13}C NMR spectrum of *O*-(*E*-6-octadecenyl) hepta-acetyl-maltose.

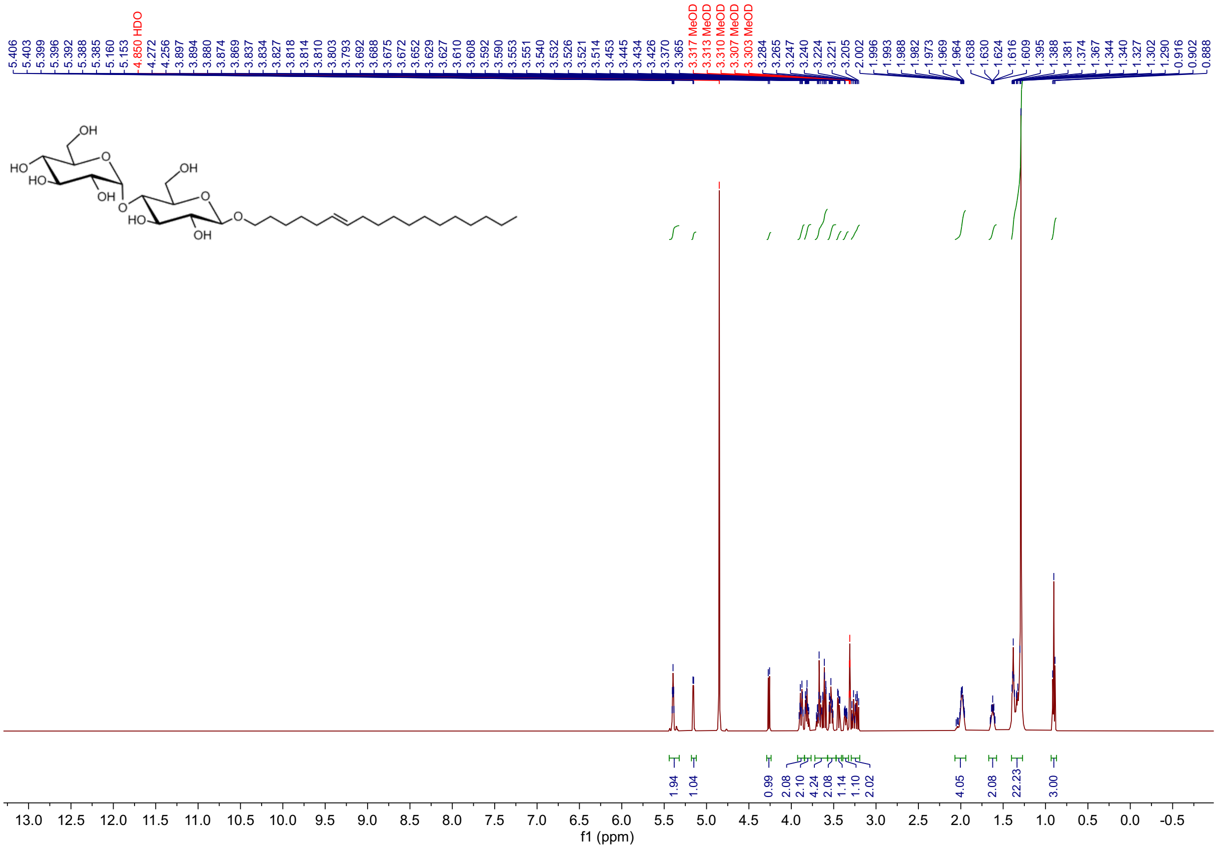

Fig. S3. ^{1}H NMR spectrum of *O*- (*E*-6-octadecenyl)-β-D-maltopyranoside

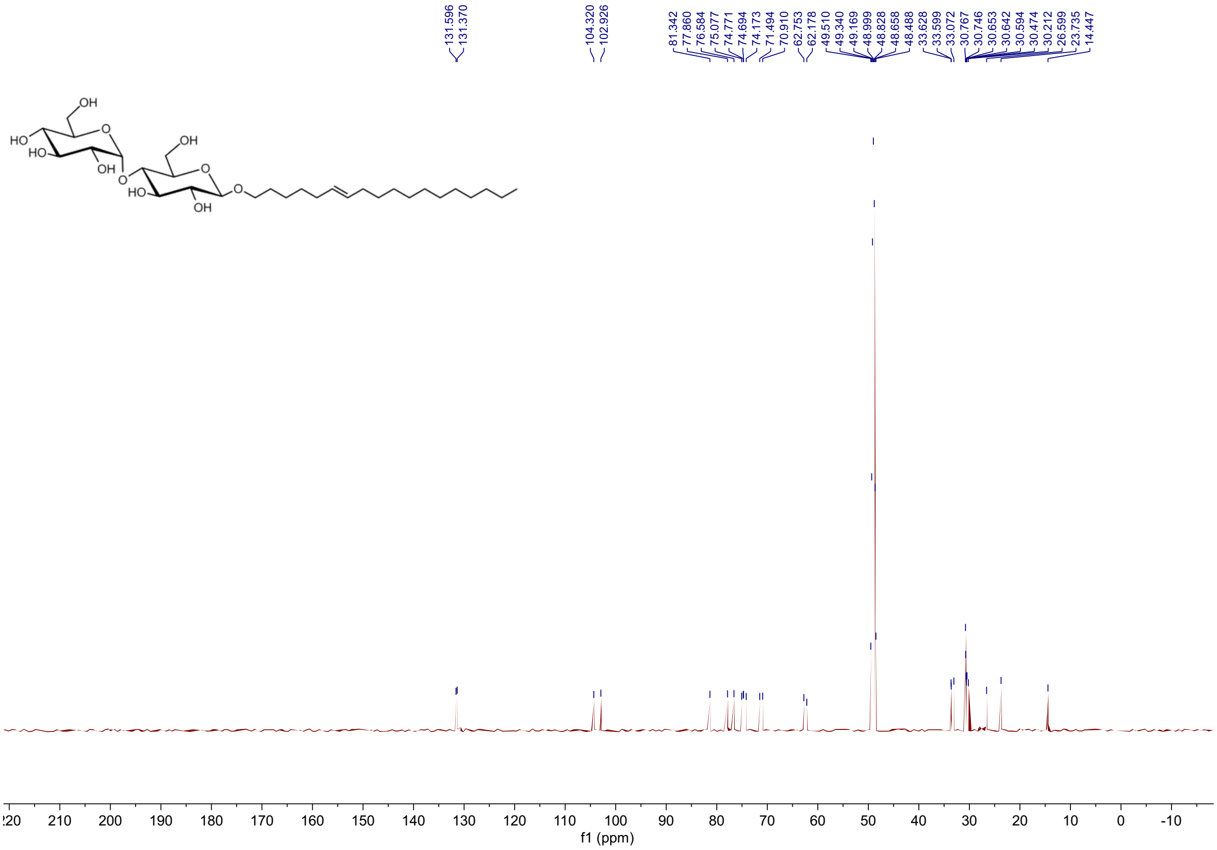

Fig. S4. ^{13}C NMR spectrum of *O*- (*E*-6-octadecenyl)-β-D-maltopyranoside.

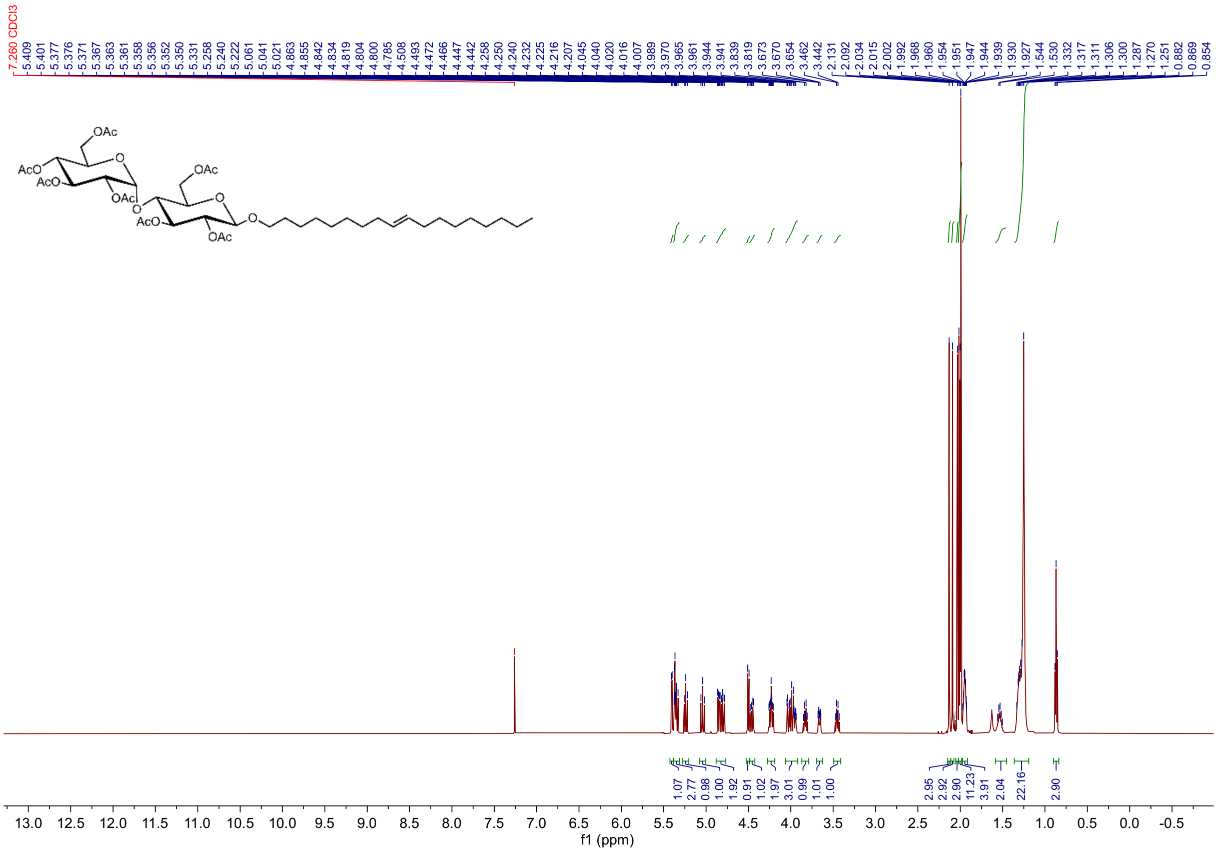

Fig. S5. ¹H NMR spectrum of *O*-(*E*-9-octadecenyl) hepta-acetyl-maltose.

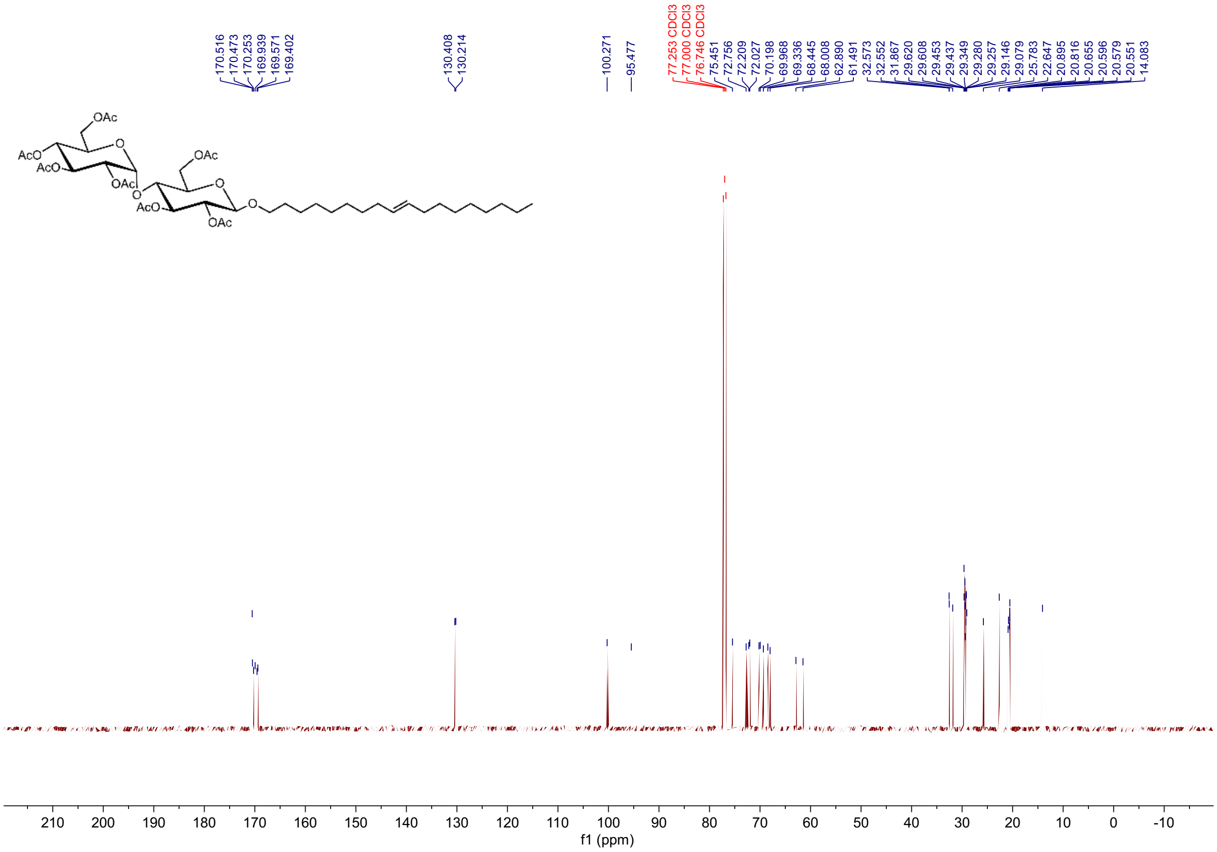

Fig. S6. ¹³C NMR spectrum of *O*-(*E*-9-octadecenyl) hepta-acetyl-maltose.

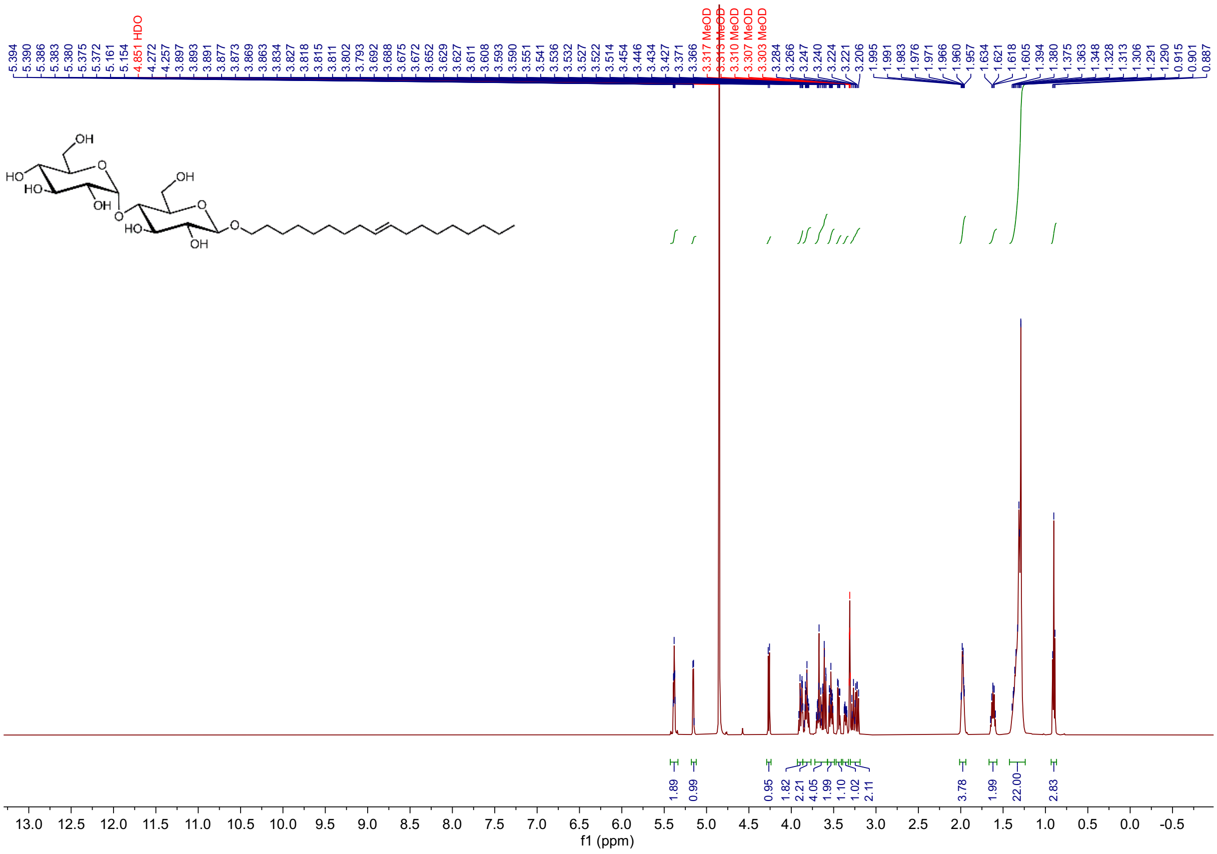

Fig. S7. ^{1}H NMR spectrum of *O*-(*E*-9-octadecenyl)-β-D-maltopyranoside

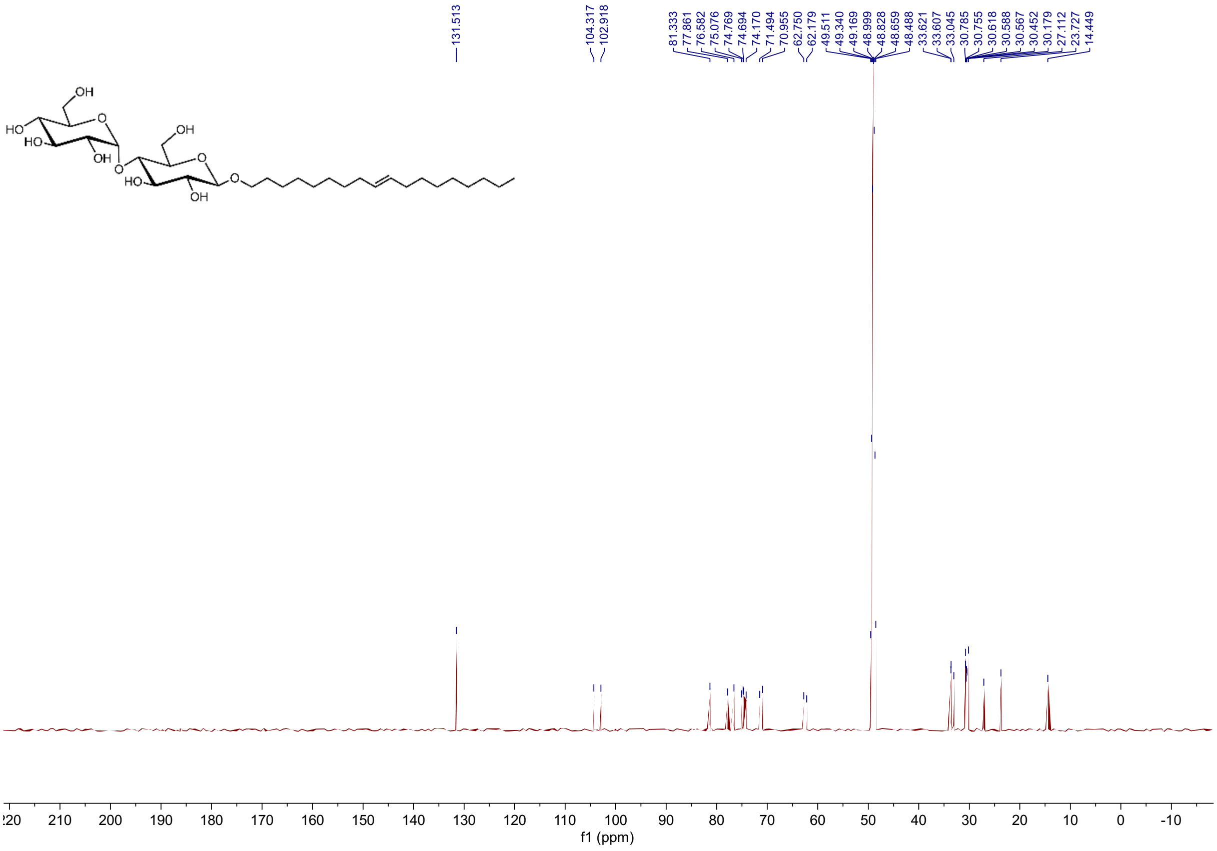

Fig. S8. ^{13}C NMR spectrum of *O*-(*E*-9-octadecenyl)-β-D-maltopyranoside.

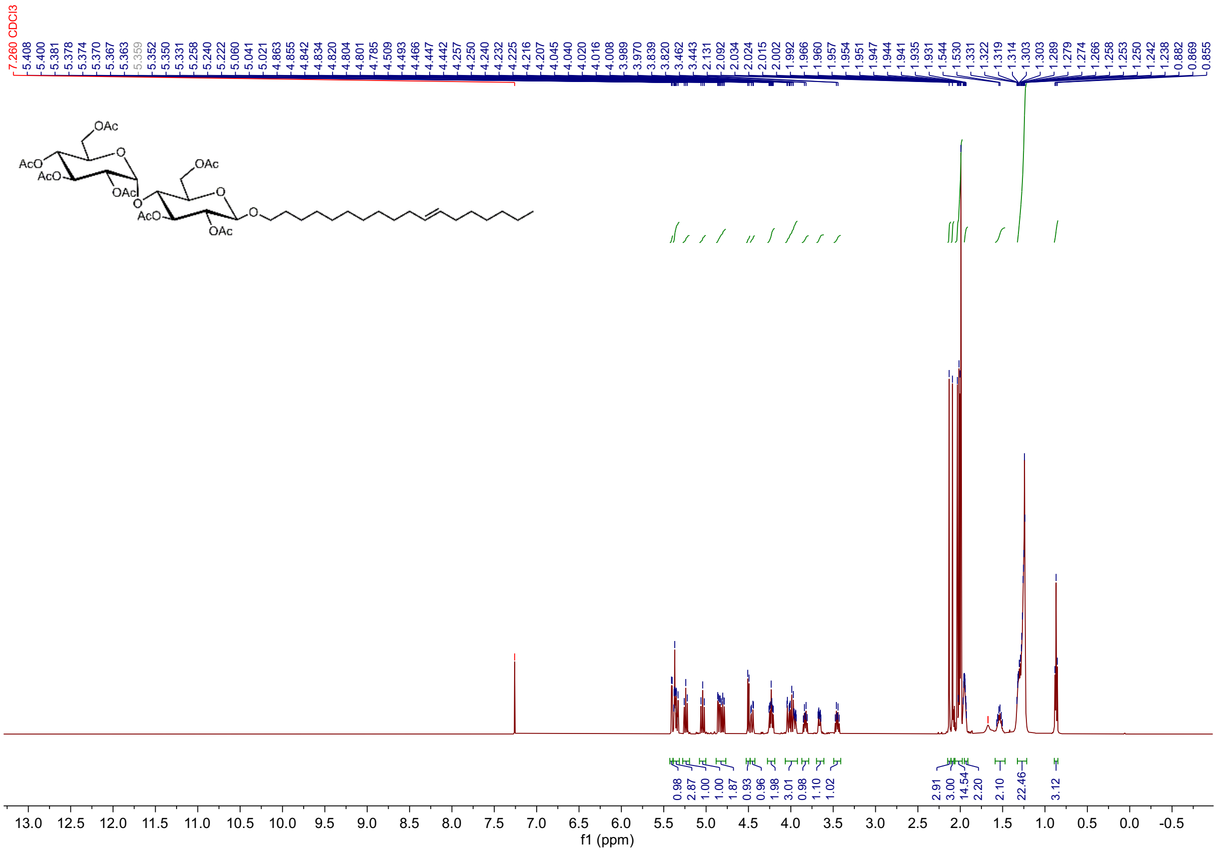

Fig. S9. ^{1}H NMR spectrum of *O*-(*E*-11-octadecenyl) hepta-acetyl-maltose.

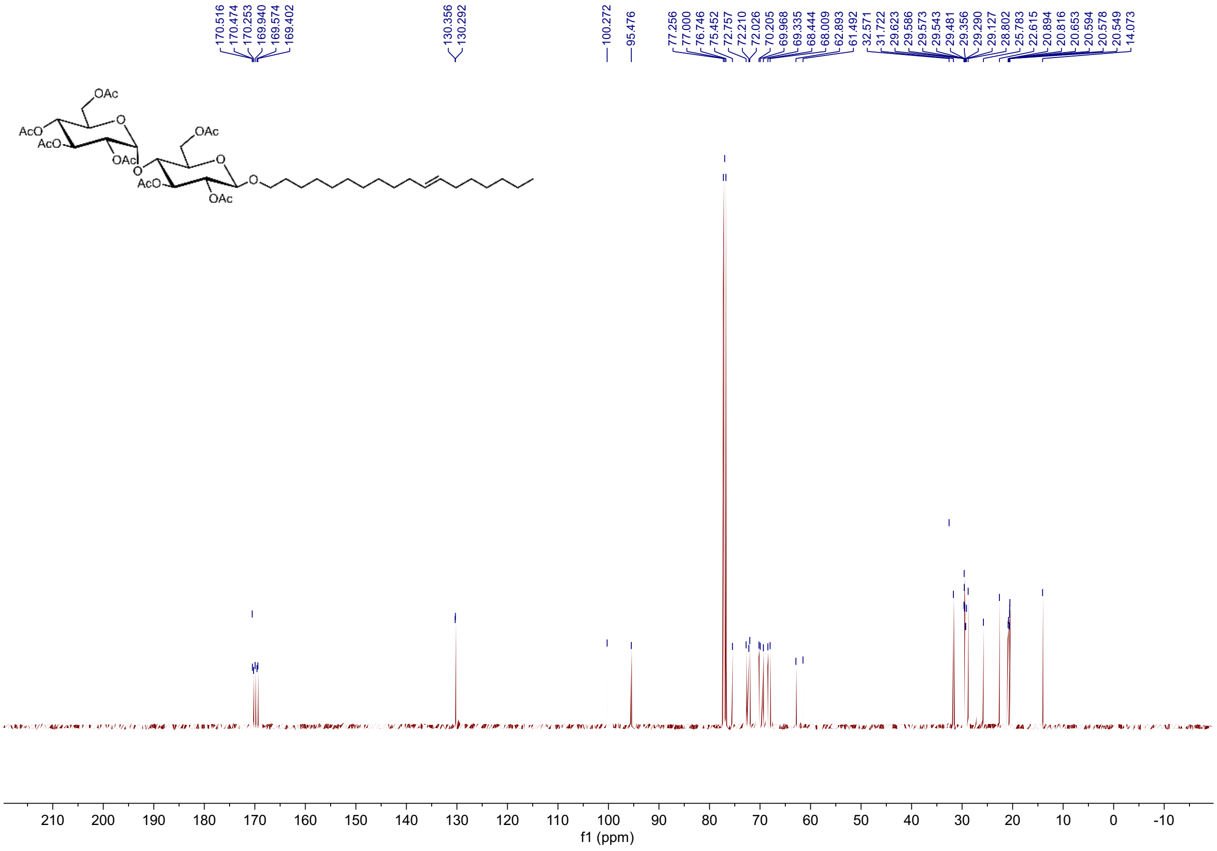

Fig. S10. ^{13}C NMR spectrum of *O*-(*E*-11-octadecenyl) hepta-acetyl-maltose.

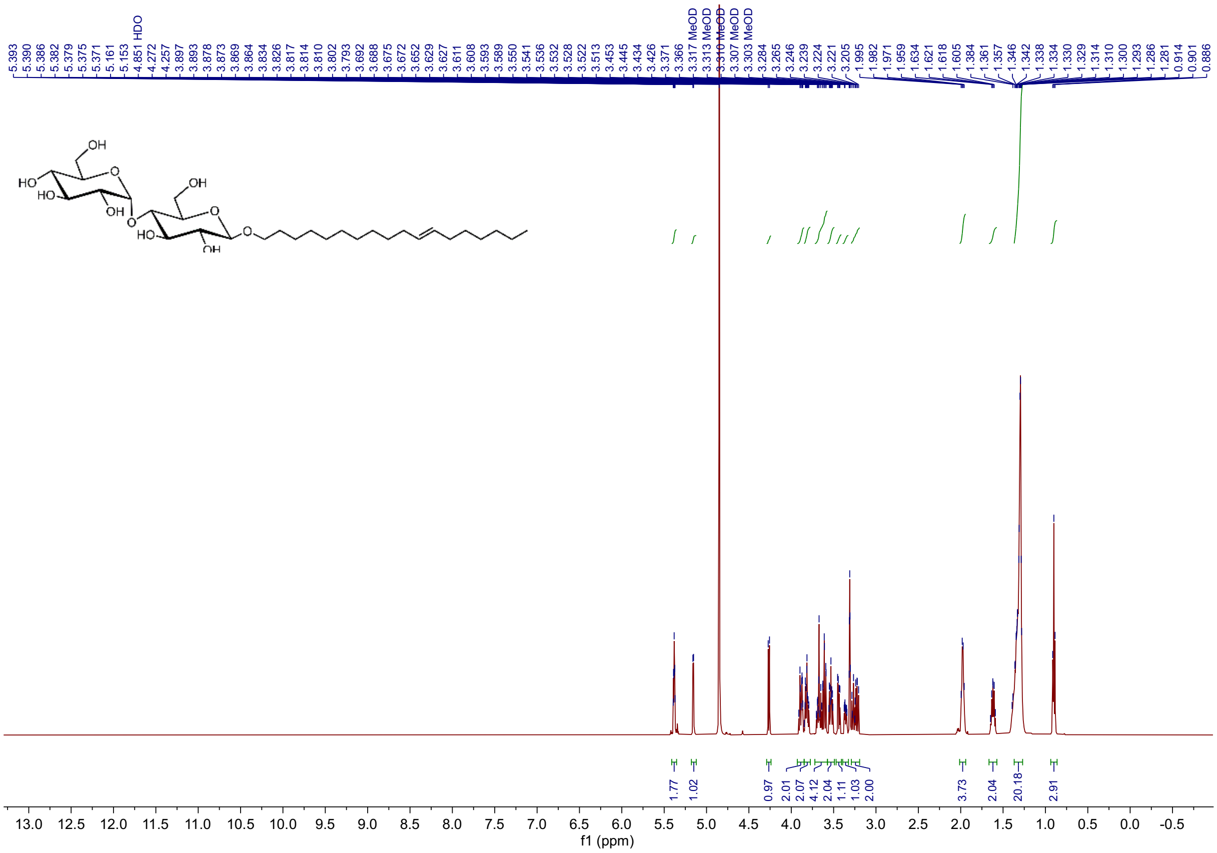

Fig. S11. ^{1}H NMR spectrum of *O*-(*E*-11-octadecenyl)-β-D-maltopyranoside

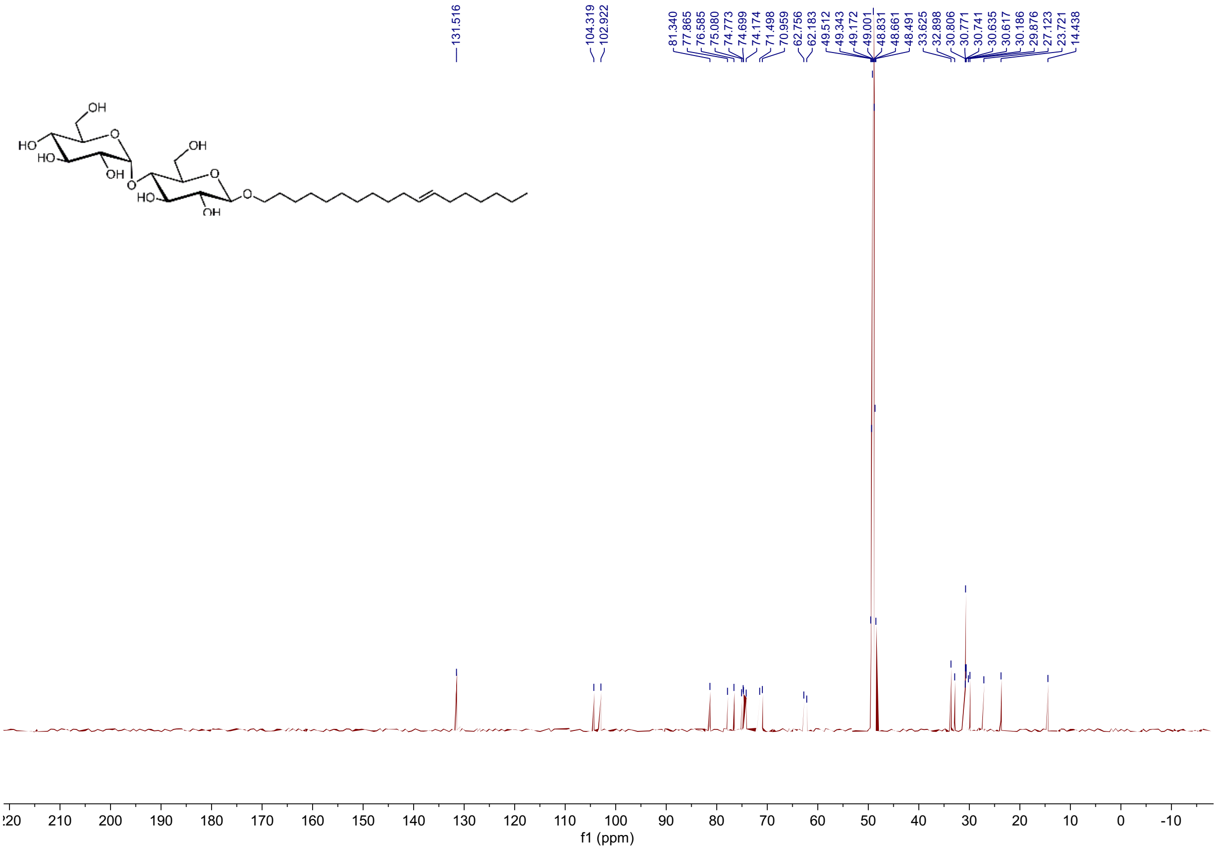

Fig. S12. ^{13}C NMR spectrum of *O*-(*E*-11-octadecenyl)-β-D-maltopyranoside.

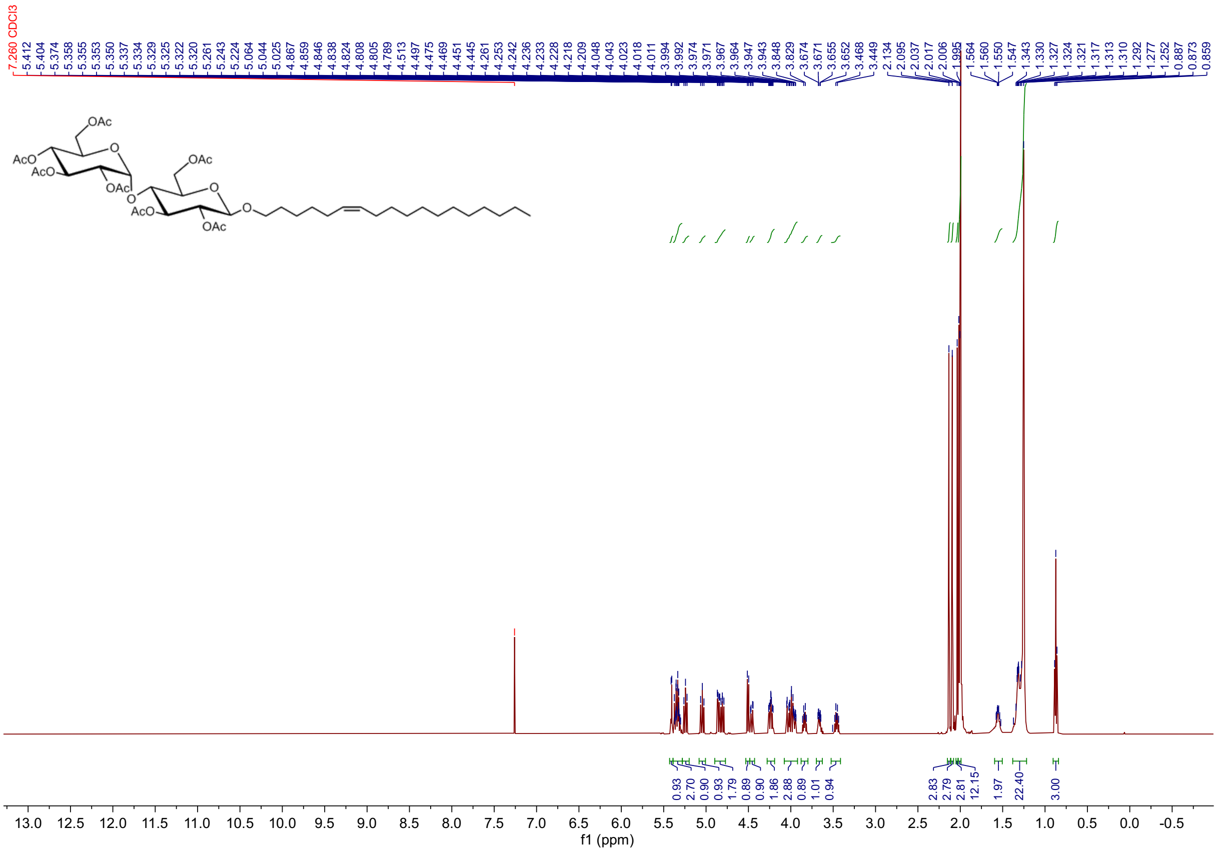

Fig. S13. ^{1}H NMR spectrum of *O*-(*Z*-6-octadecenyl) hepta-acetyl-maltose.

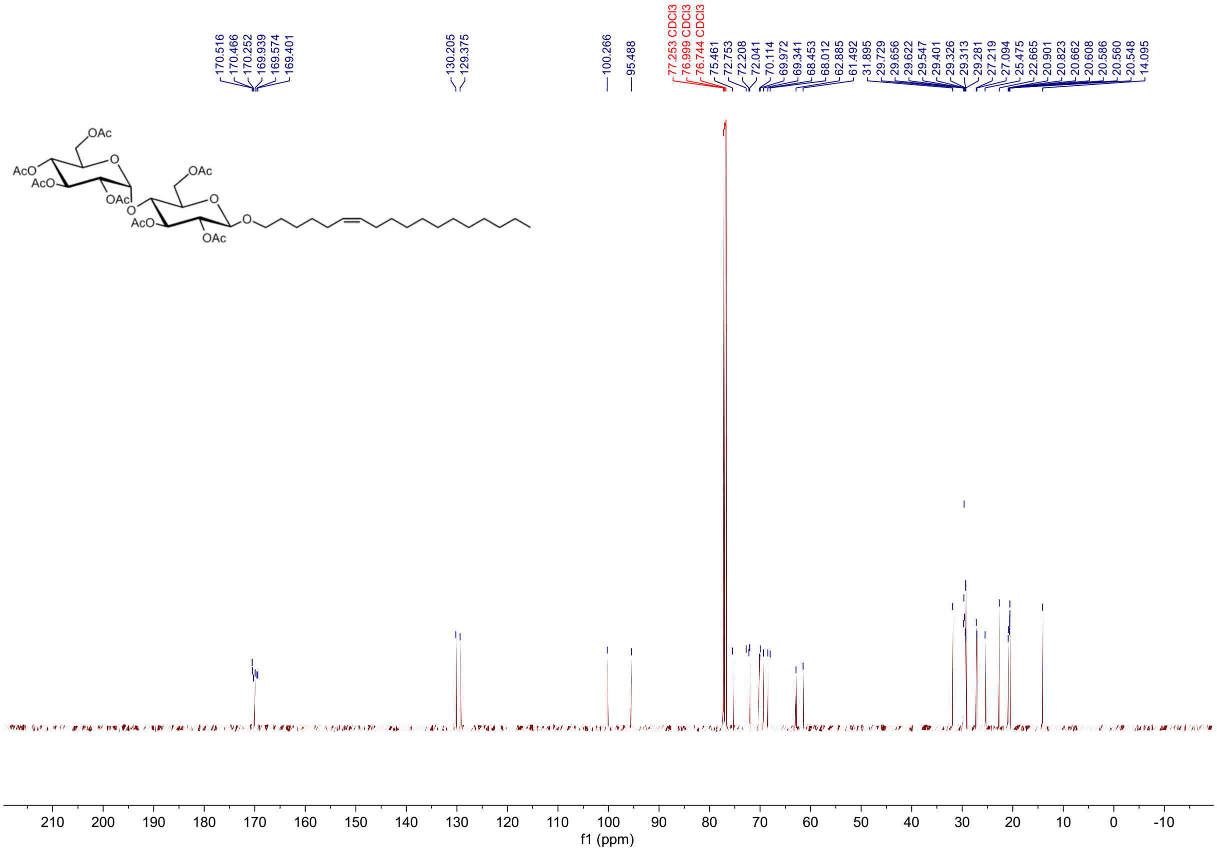

Fig. S14. ^{13}C NMR spectrum of *O*-(*Z*-6-octadecenyl) hepta-acetyl-maltose.

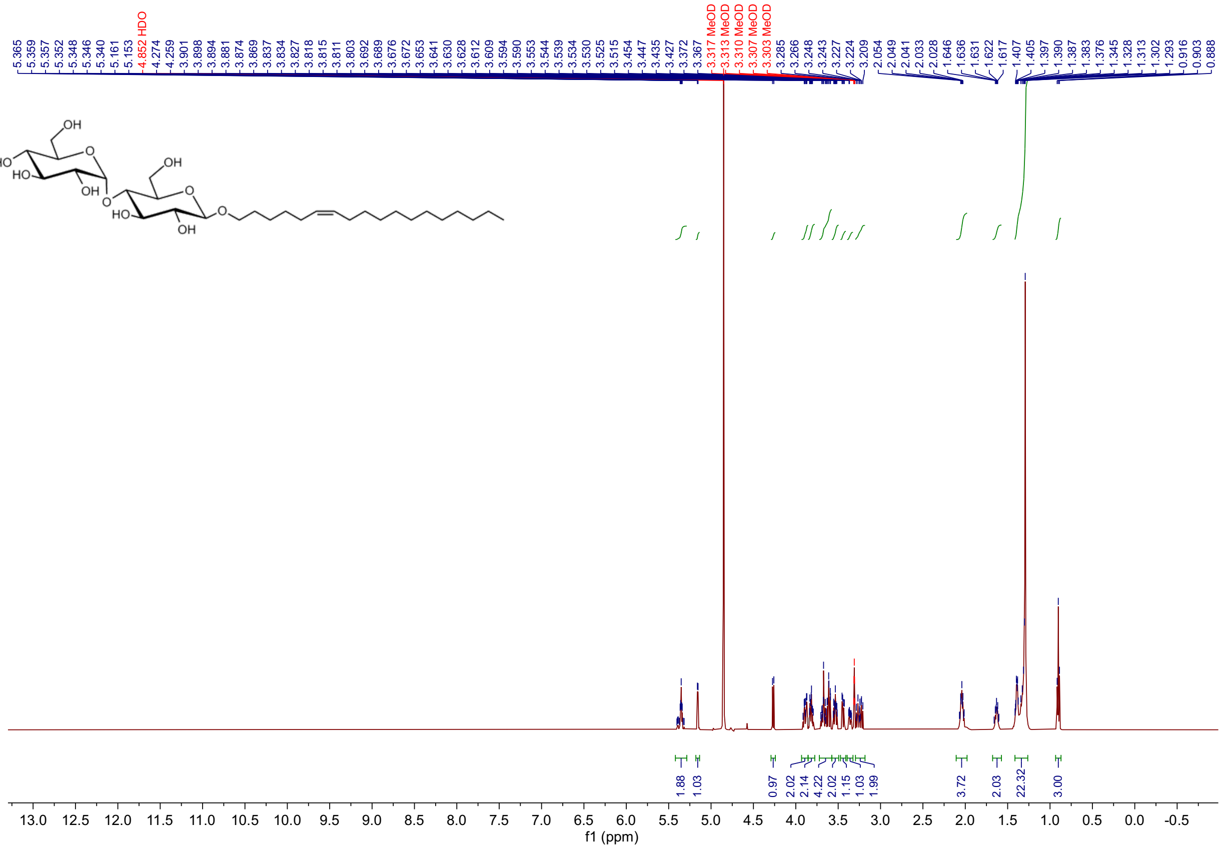

Fig. S15. ^{1}H NMR spectrum of *O*-(*Z*-6-octadecenyl)-β-D-maltopyranoside

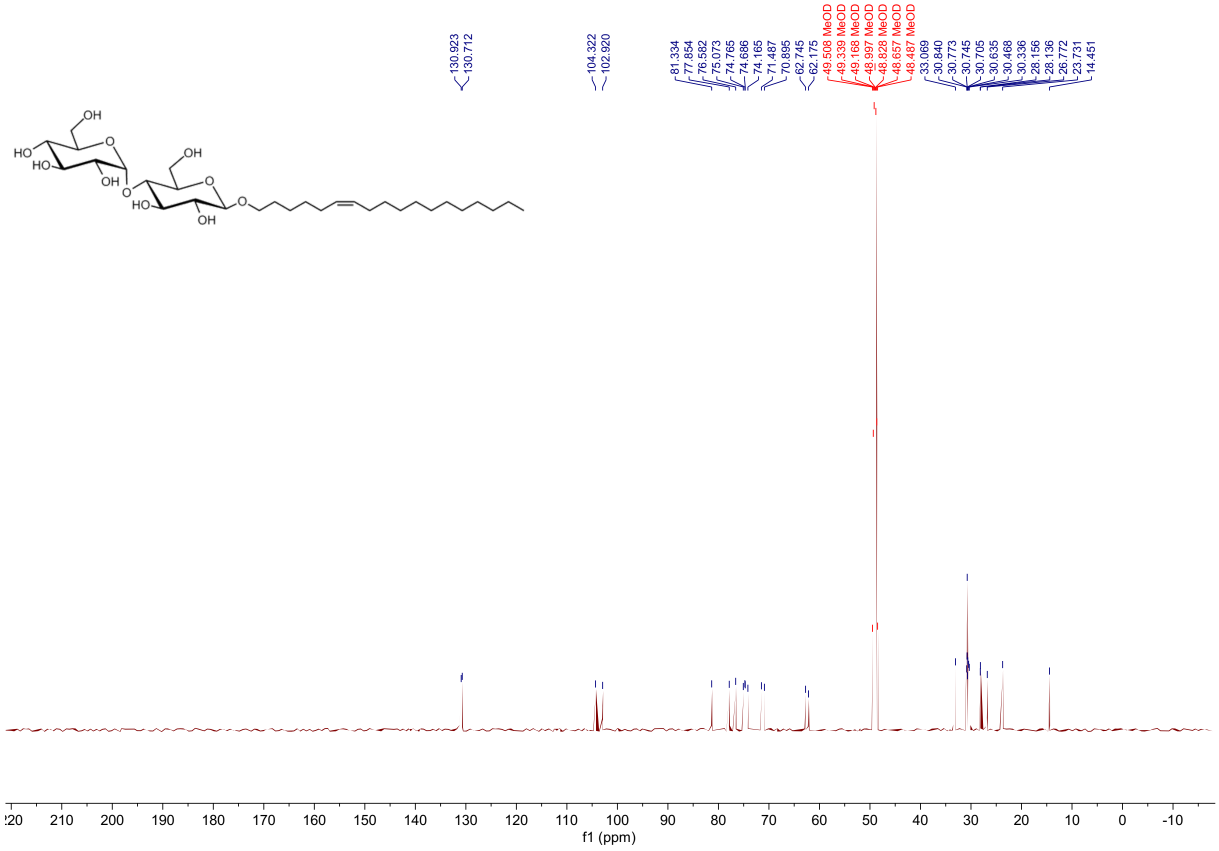

Fig. S16. ^{13}C NMR spectrum of *O*-(*Z*-6-octadecenyl)-β-D-maltopyranoside.

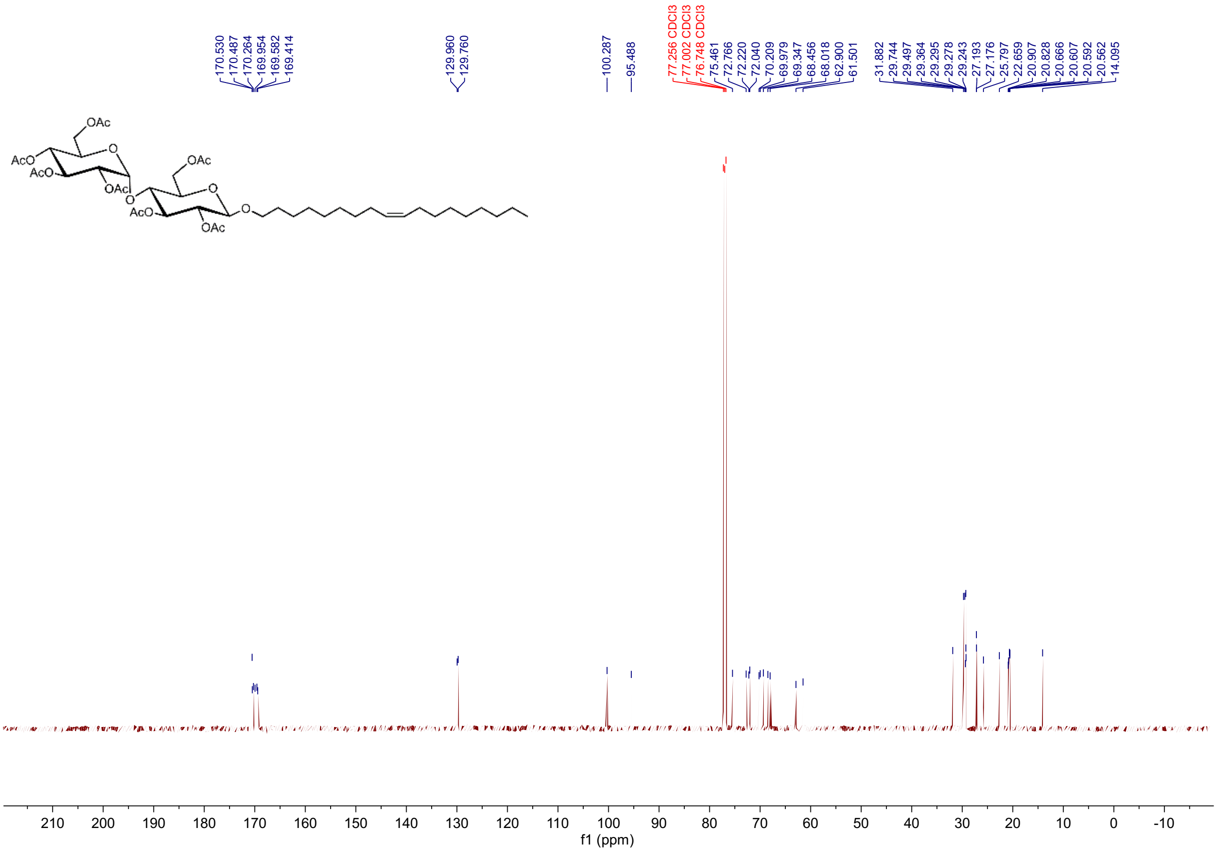

Fig. S17. ¹H NMR spectrum of *O*-(*Z*-9-octadecenyl) hepta-acetyl-maltose.

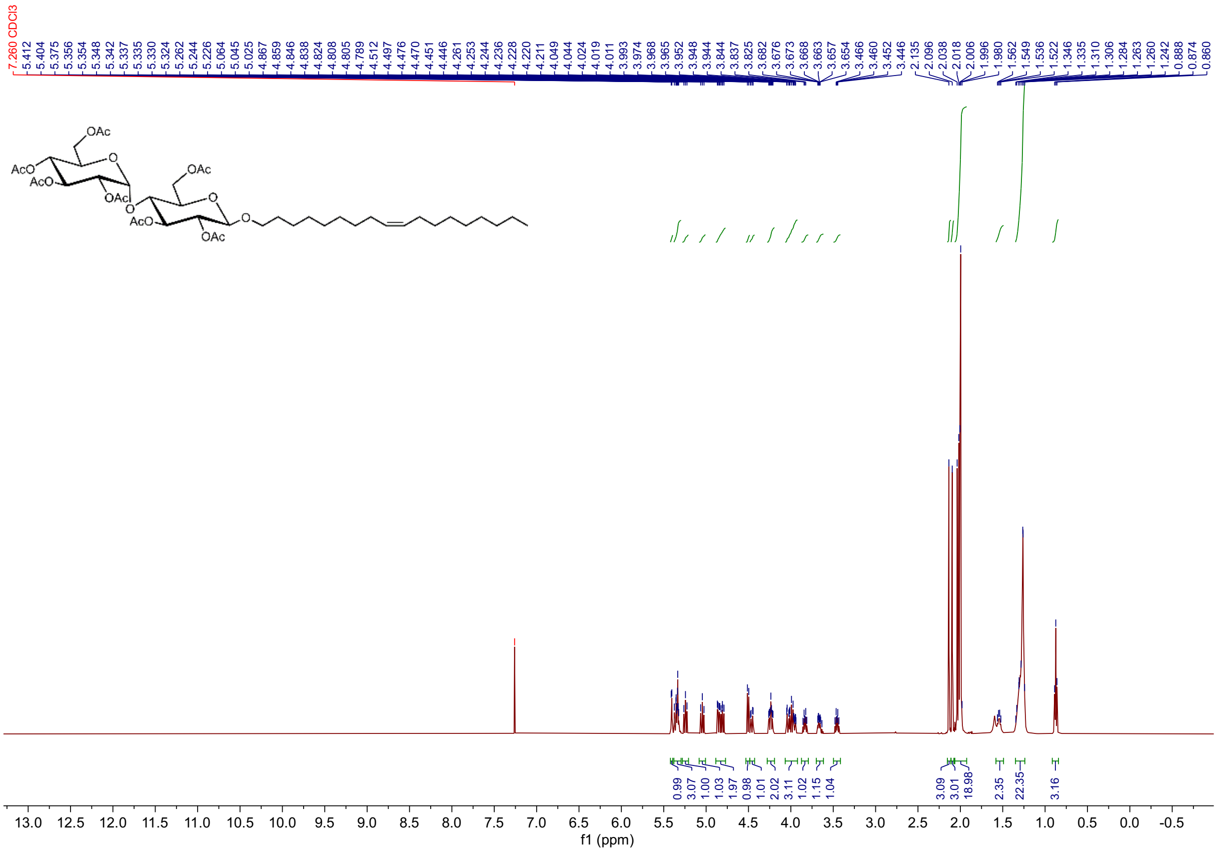

Fig. S18. ¹³C NMR spectrum of *O*-(*Z*-9-octadecenyl) hepta-acetyl-maltose.

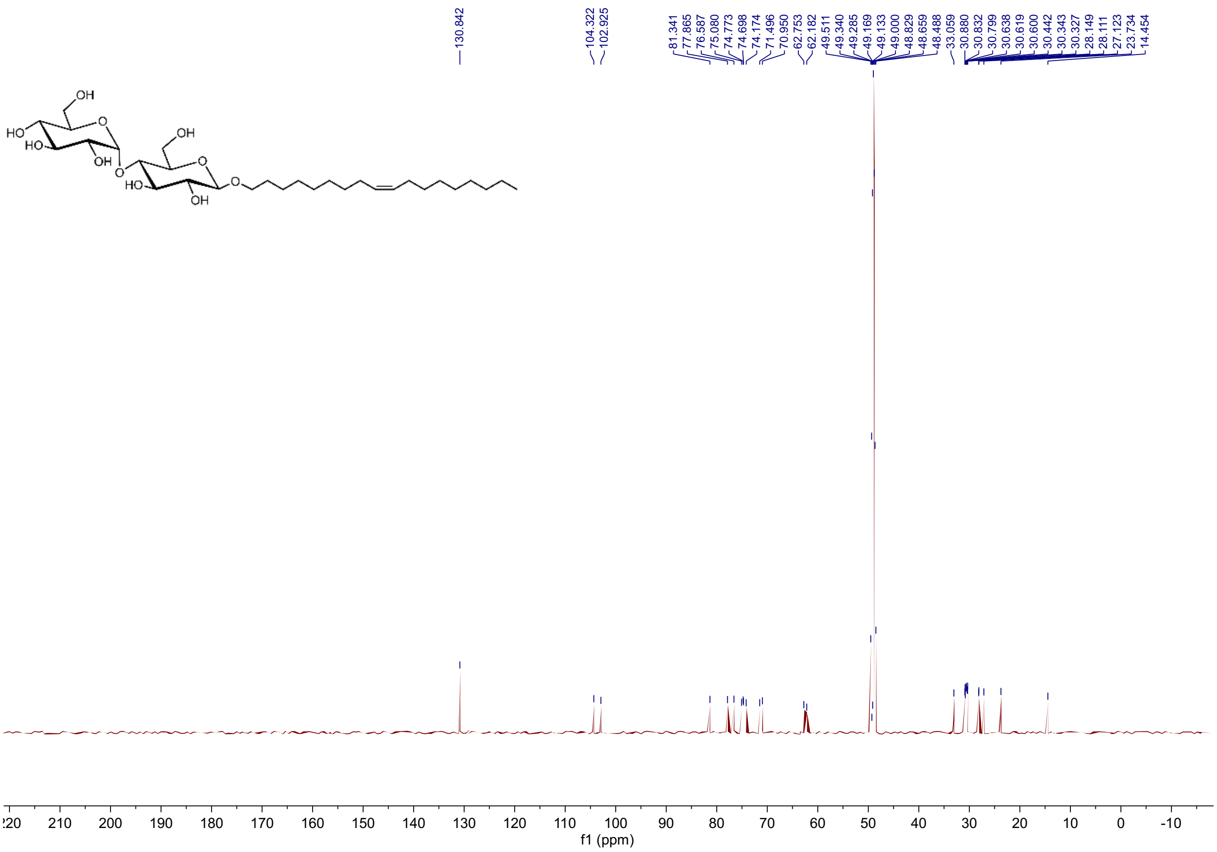

Fig. S19. ¹H NMR spectrum of *O*-(*Z*-9-octadecenyl)-β-D-maltopyranoside

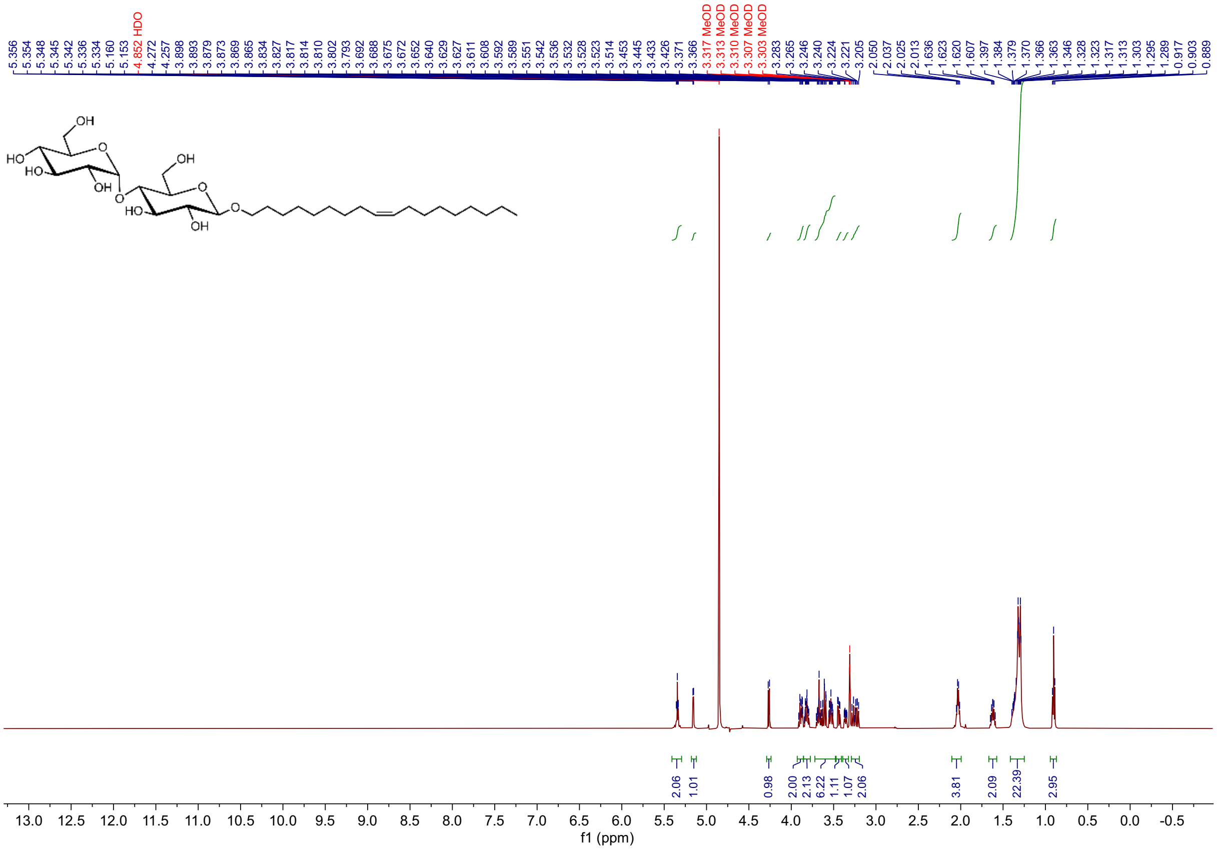

Fig. S20. ¹³C NMR spectrum of *O*-(*Z*-9-octadecenyl)-β-D-maltopyranoside.

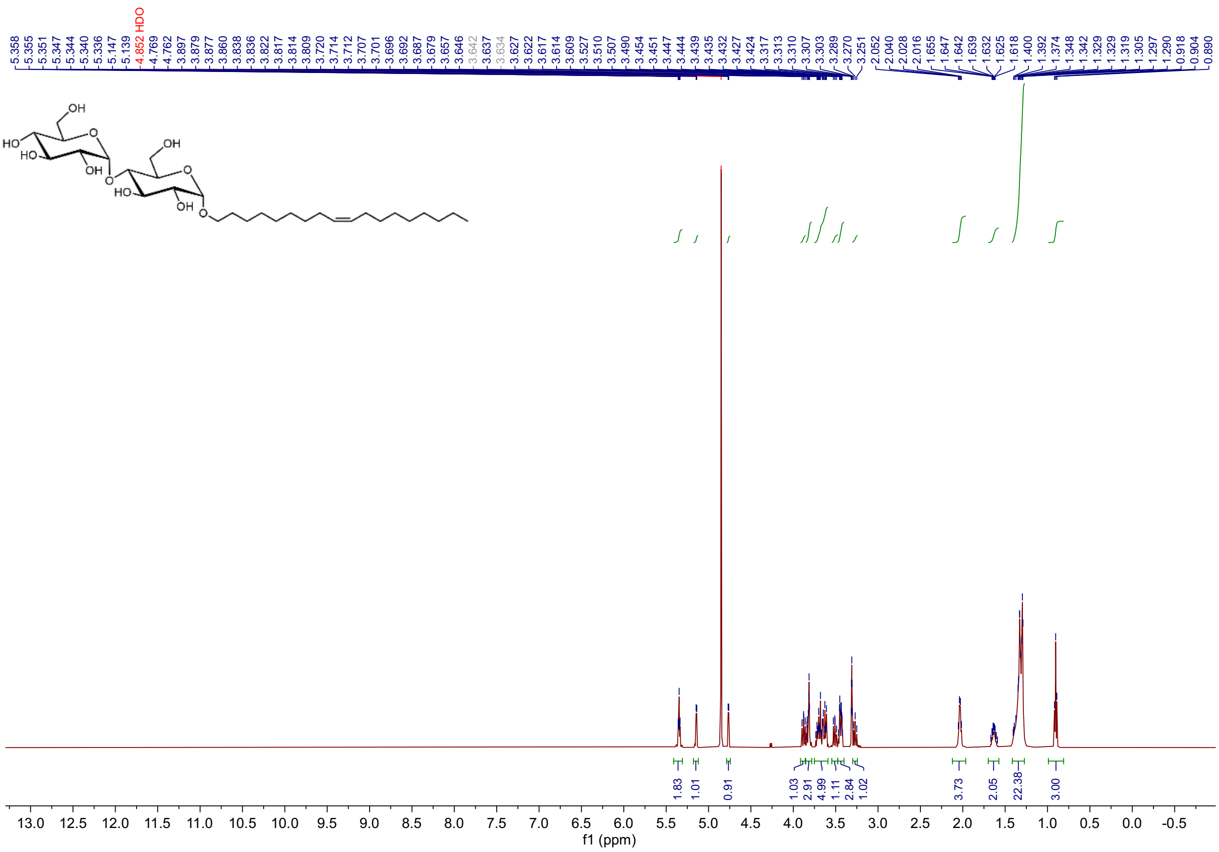

Fig. S21. ^{1}H NMR spectrum of *O*-(*Z*-9-octadecenyl)-α-D-maltopyranoside

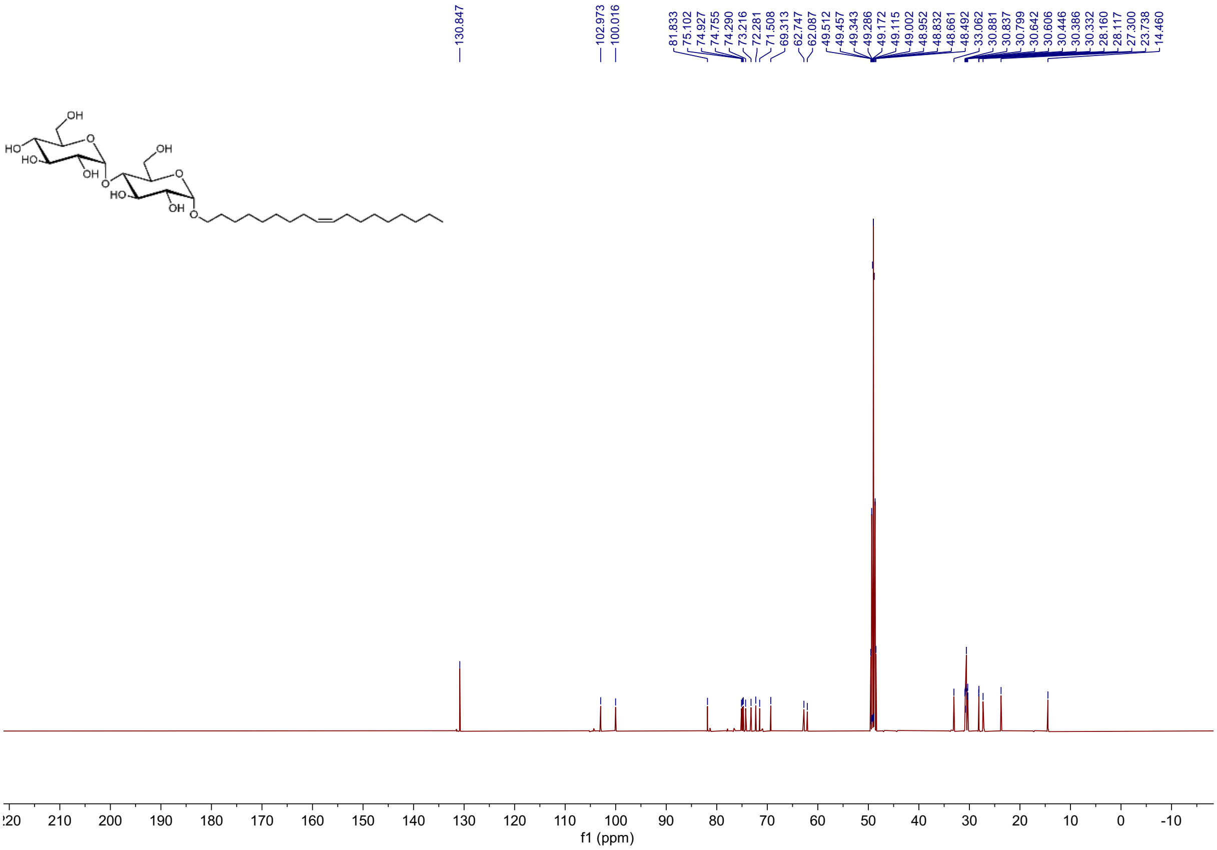

Fig. S22. ^{13}C NMR spectrum of *O*-(*Z*-9-octadecenyl)-α-D-maltopyranoside

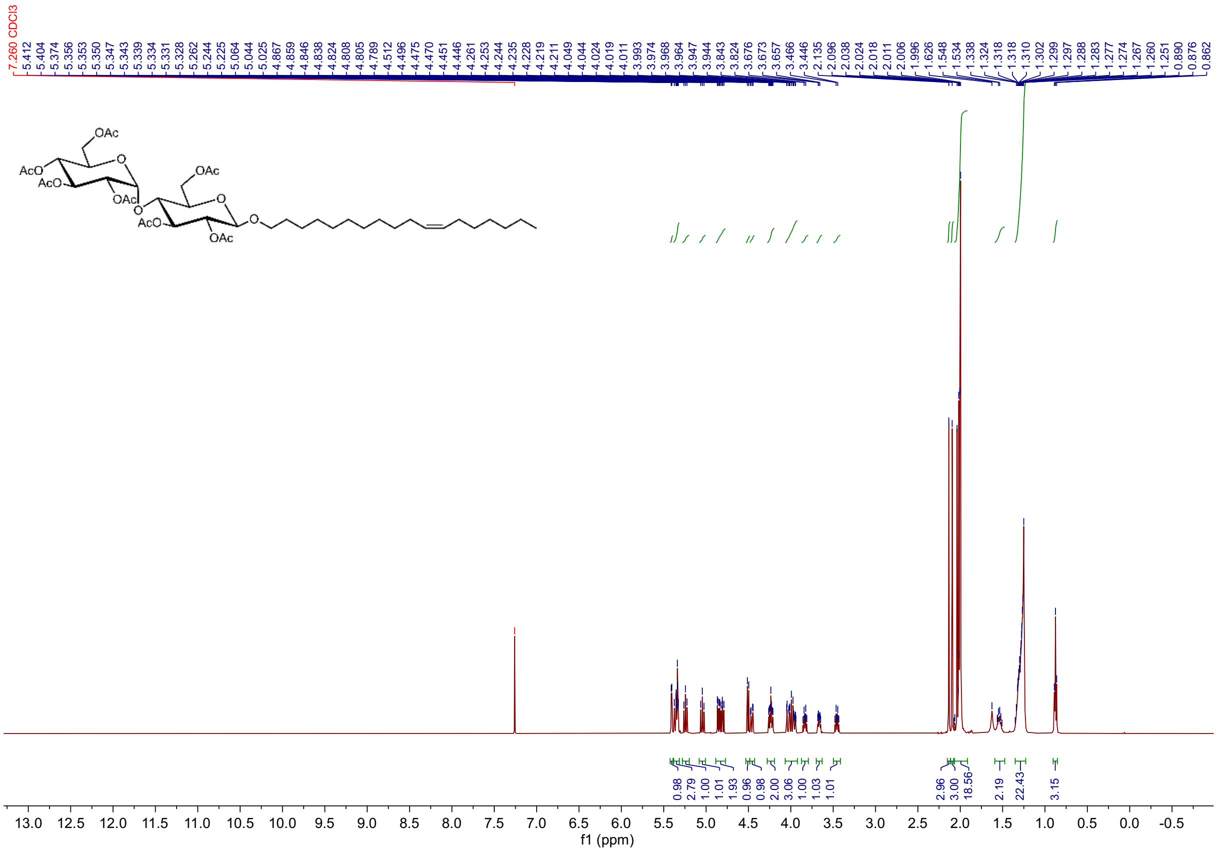

Fig. S23. ^{1}H NMR spectrum of *O*-(*Z*-11-octadecenyl) hepta-acetyl-maltose.

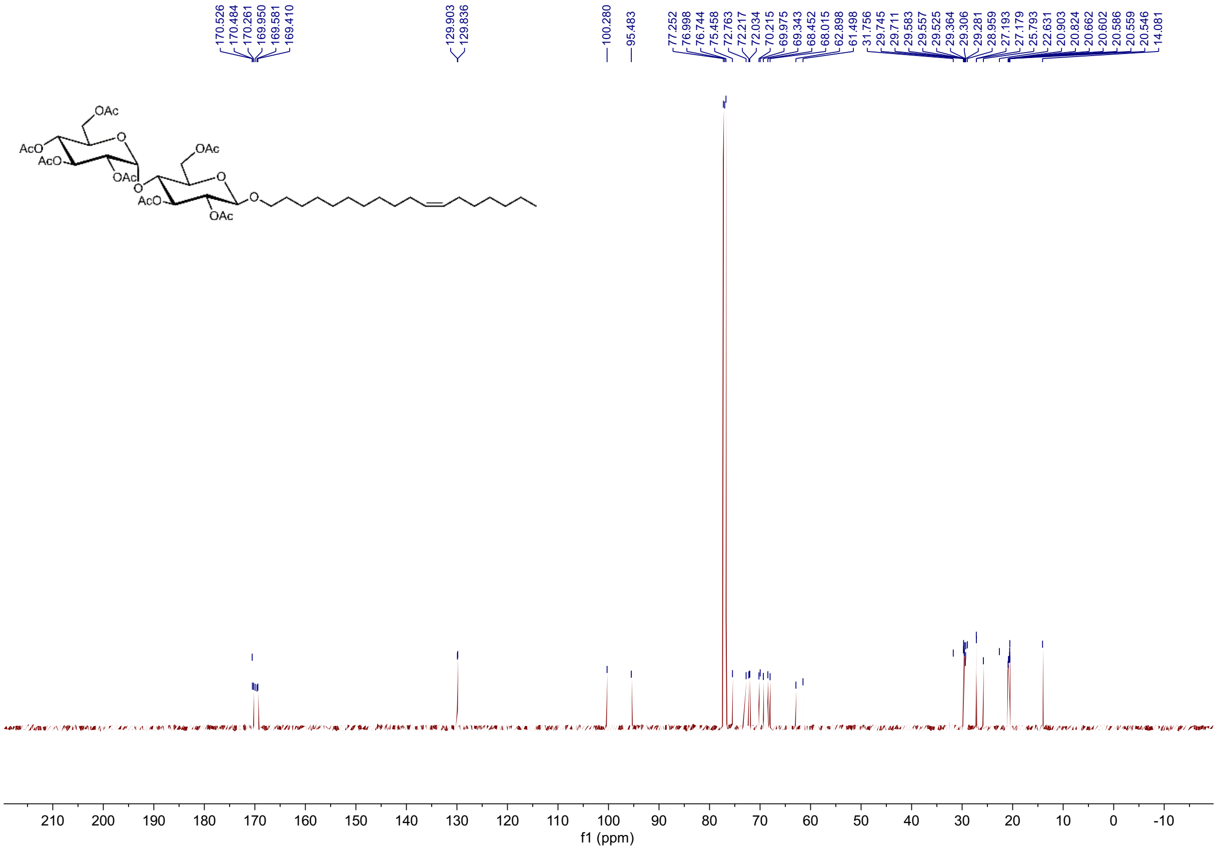

Fig. S24. ^{13}C NMR spectrum of *O*-(*Z*-11-octadecenyl) hepta-acetyl-maltose.

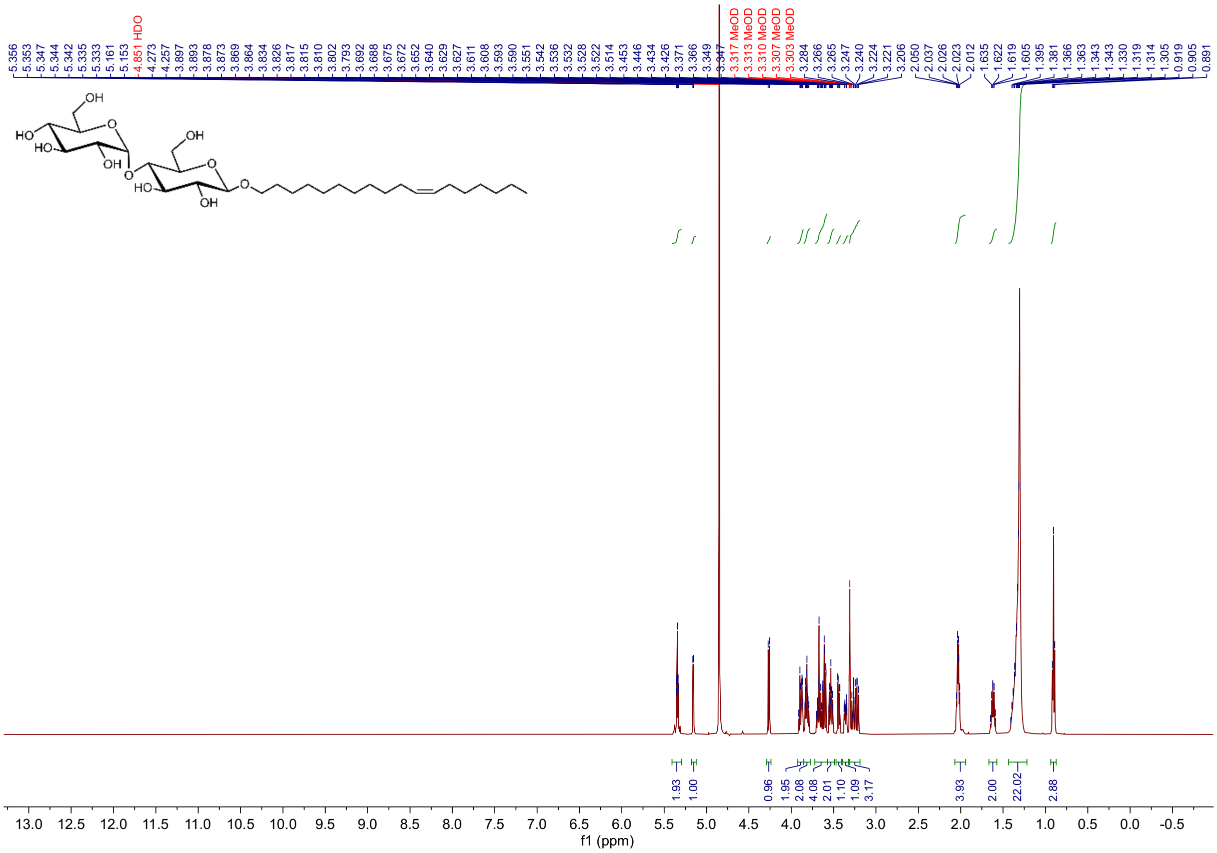

Fig. S25. ^{1}H NMR spectrum of *O*-(*Z*-11-octadecenyl)-β-D-maltopyranoside

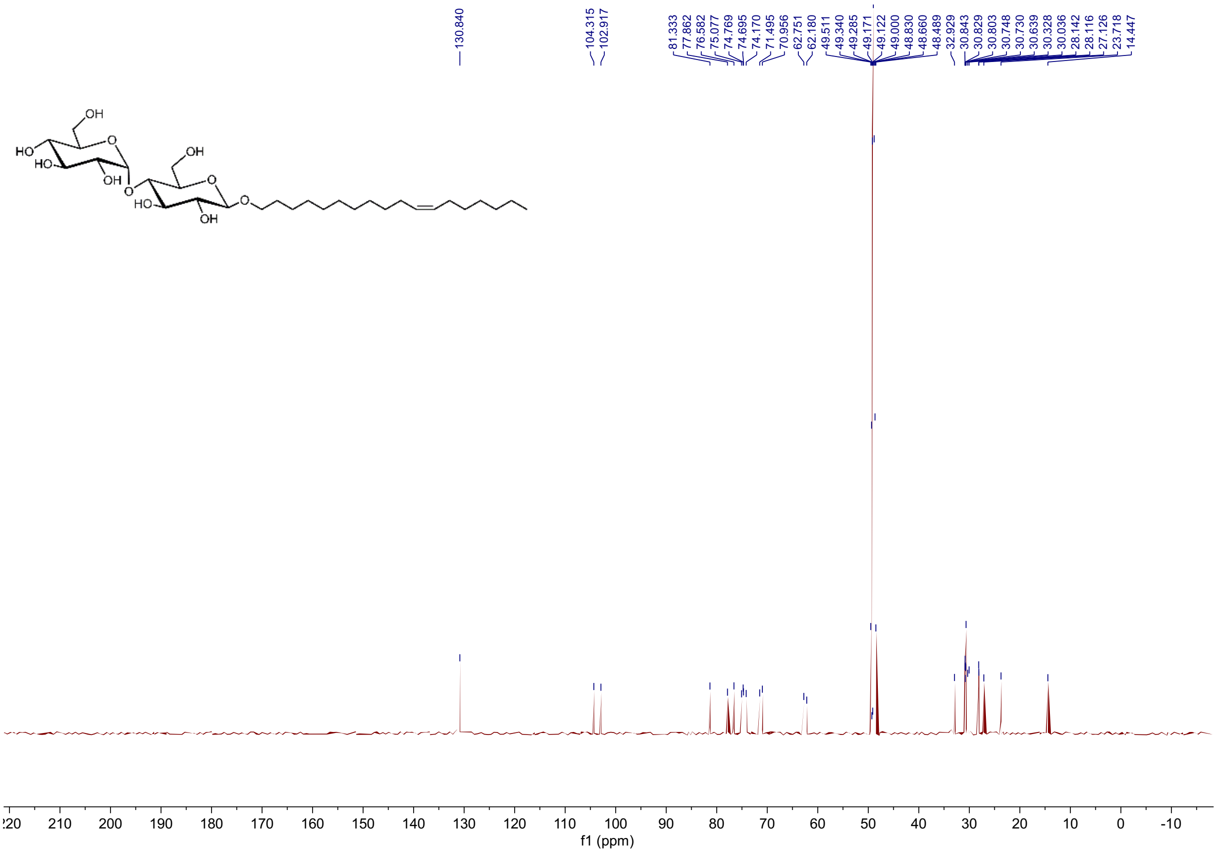

Fig. S26. ^{13}C NMR spectrum of *O*-(*Z*-11-octadecenyl)-β-D-maltopyranoside.

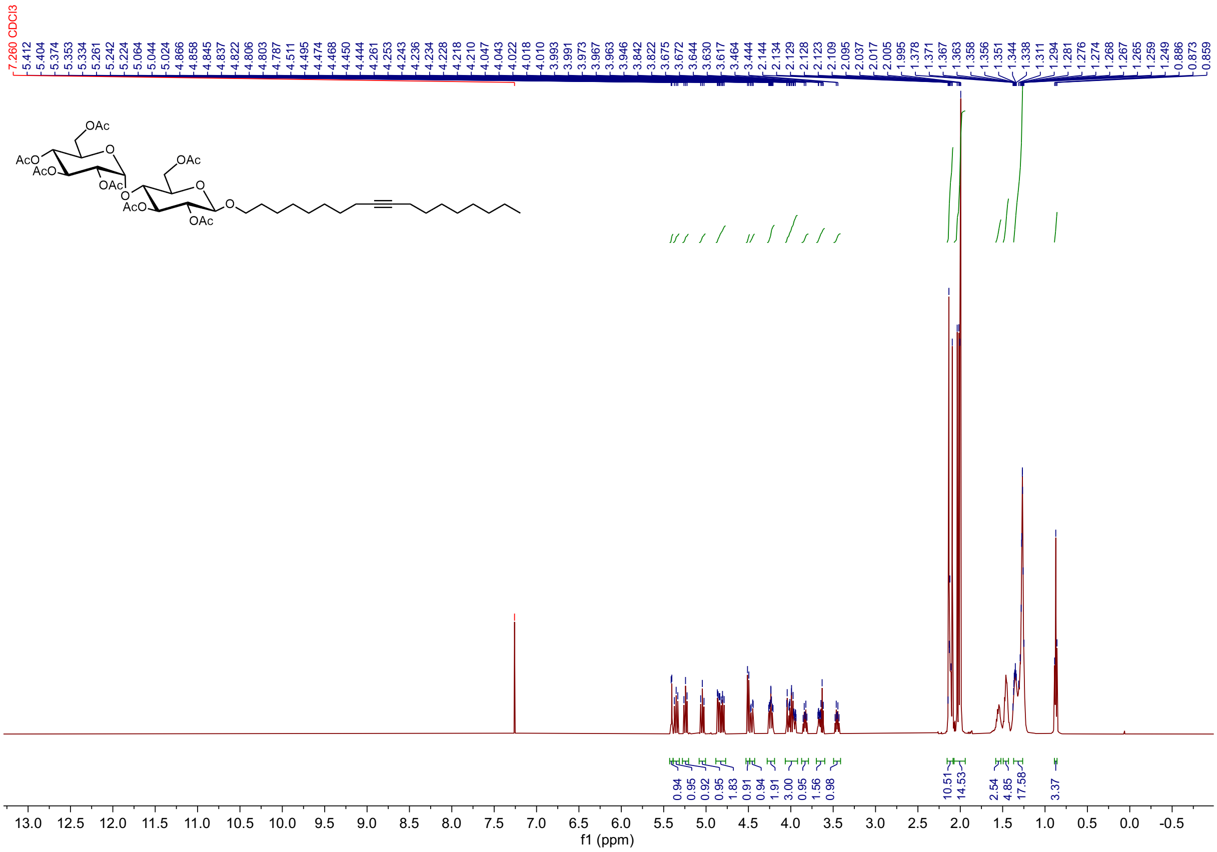

Fig. S27. ^{1}H NMR spectrum of *O*-(9-octadecynyl)-hepta-acetyl-maltose.

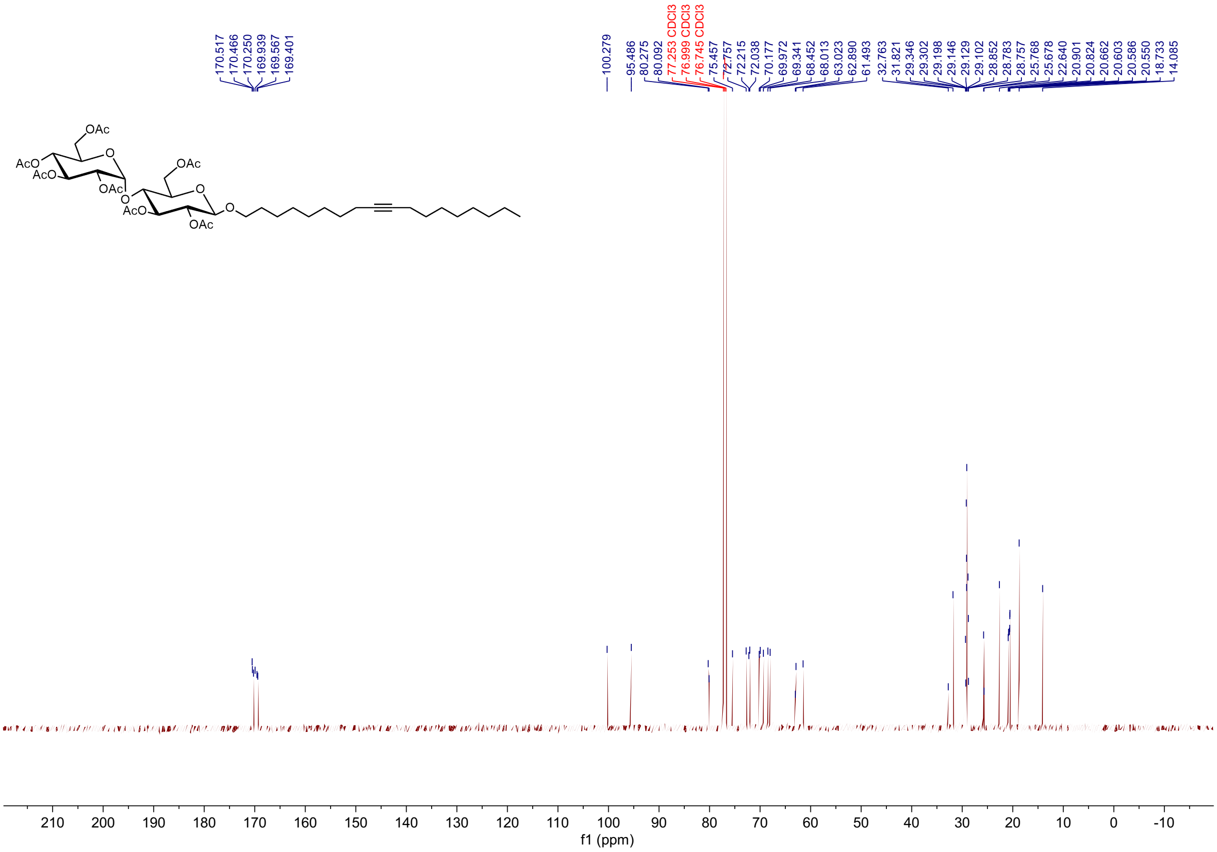

Fig. S28. ^{13}C NMR spectrum of *O*-(9-octadecynyl)-hepta-acetyl-maltose.

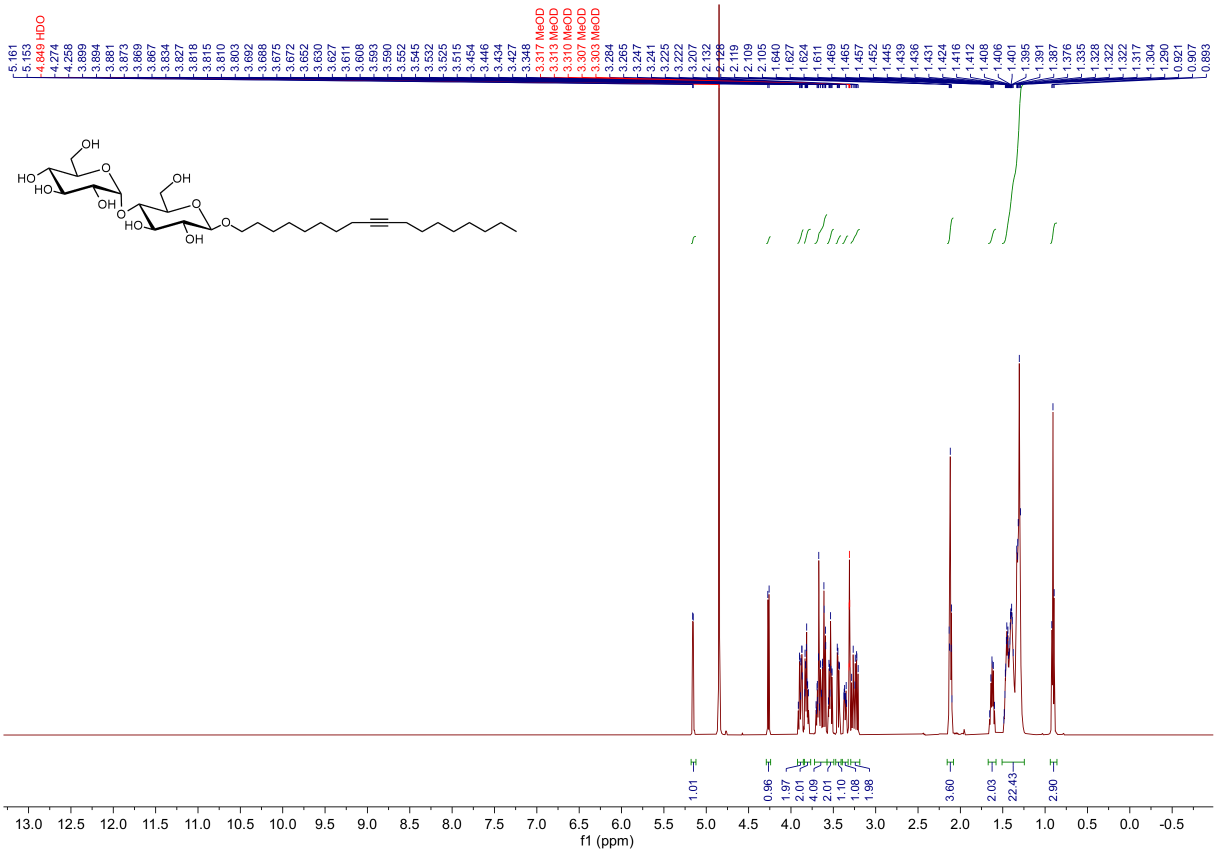

Fig. S29. ¹H NMR spectrum of (*9*-octadecynyl)-β-D-maltopyranoside.

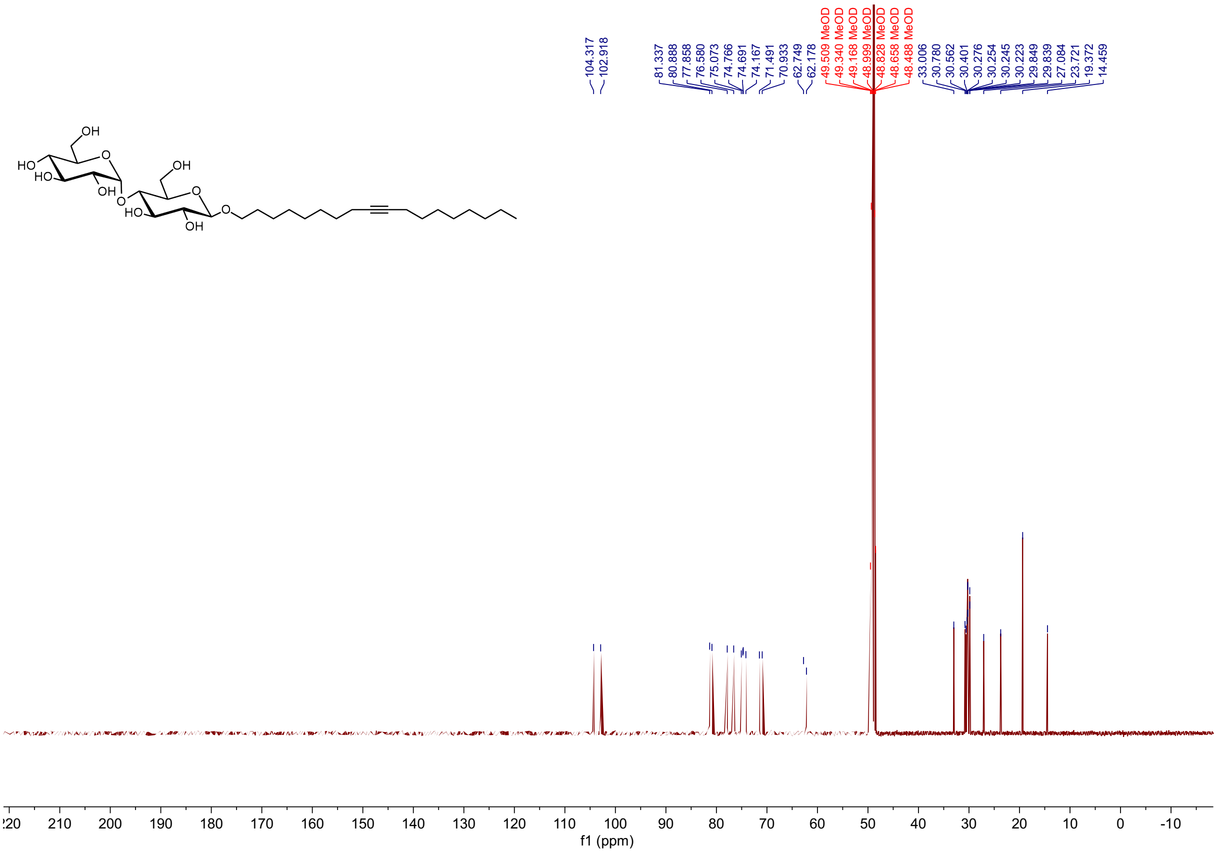

Fig. S30. ¹³C NMR spectrum of (*9*-octadecynyl)-β-D-maltopyranoside.

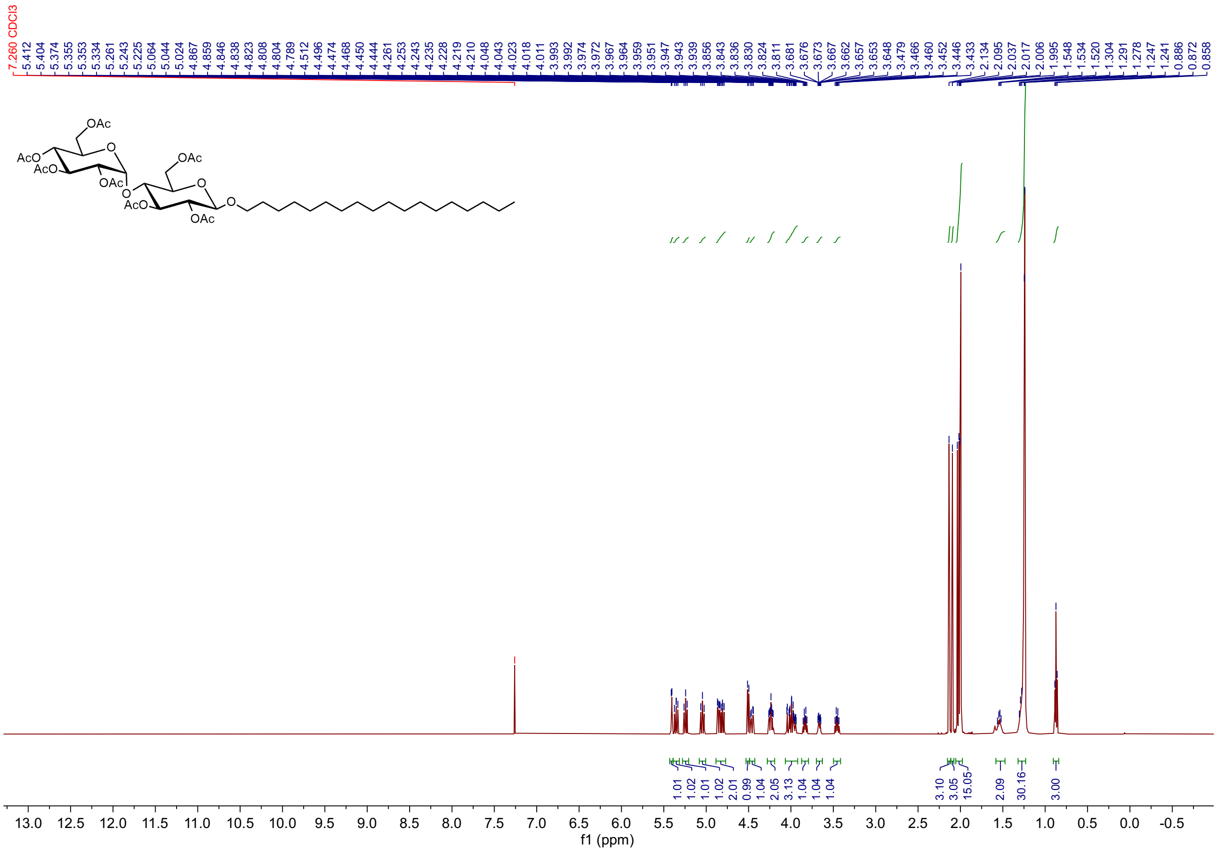

Fig. S31. ^{1}H NMR spectrum of *O*-(octadecyl)-hepta-acetyl-maltose.

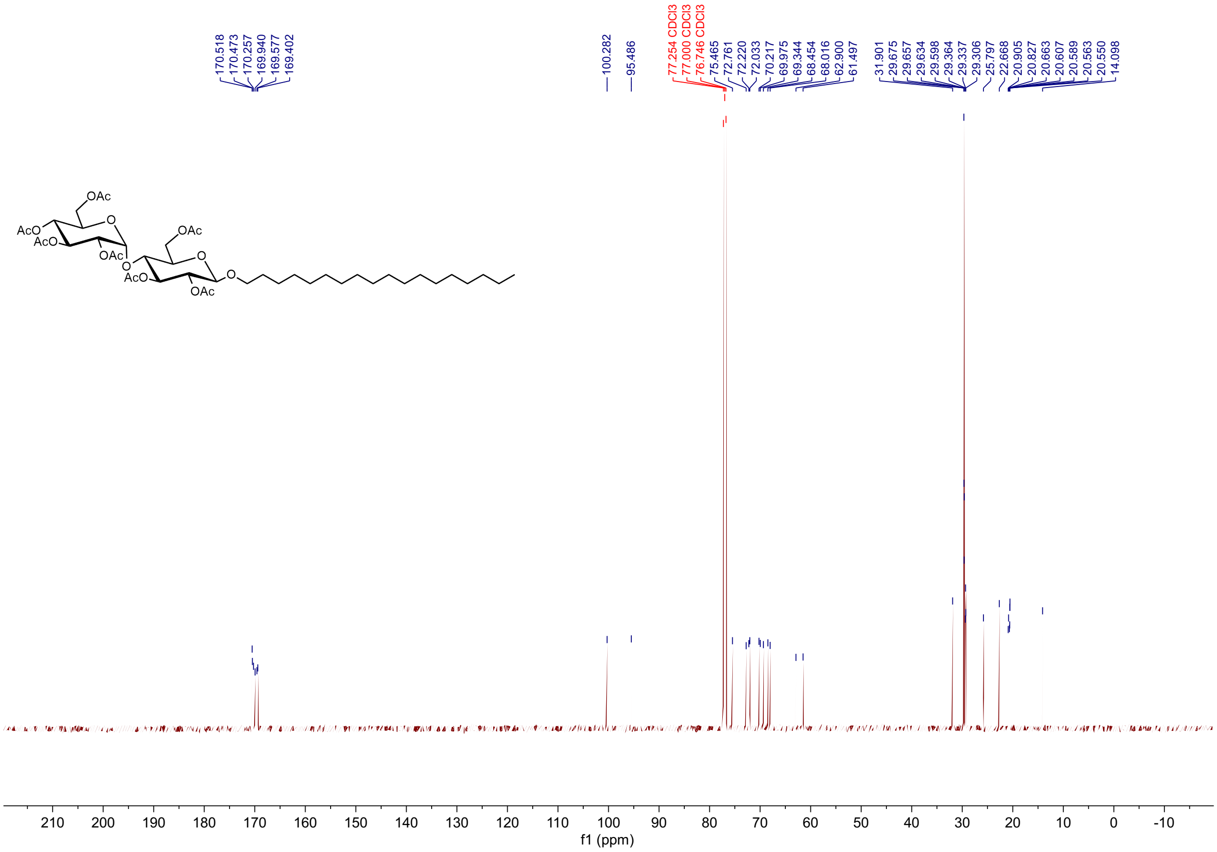

Fig. S32. ^{13}C NMR spectrum of *O*-(octadecyl)-hepta-acetyl-maltose.

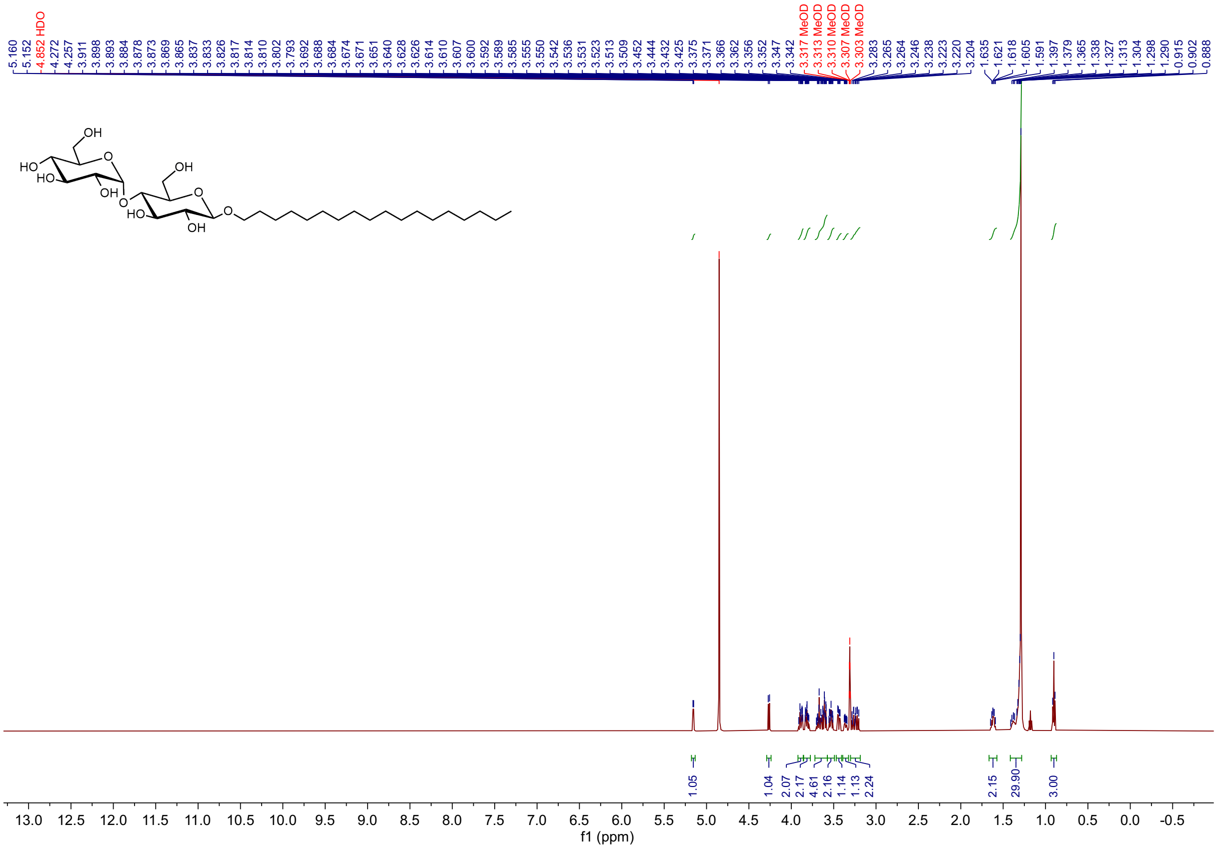

Fig. S33. ^{1}H NMR spectrum of (*9*-octadecynyl)-β-D-maltopyranoside.

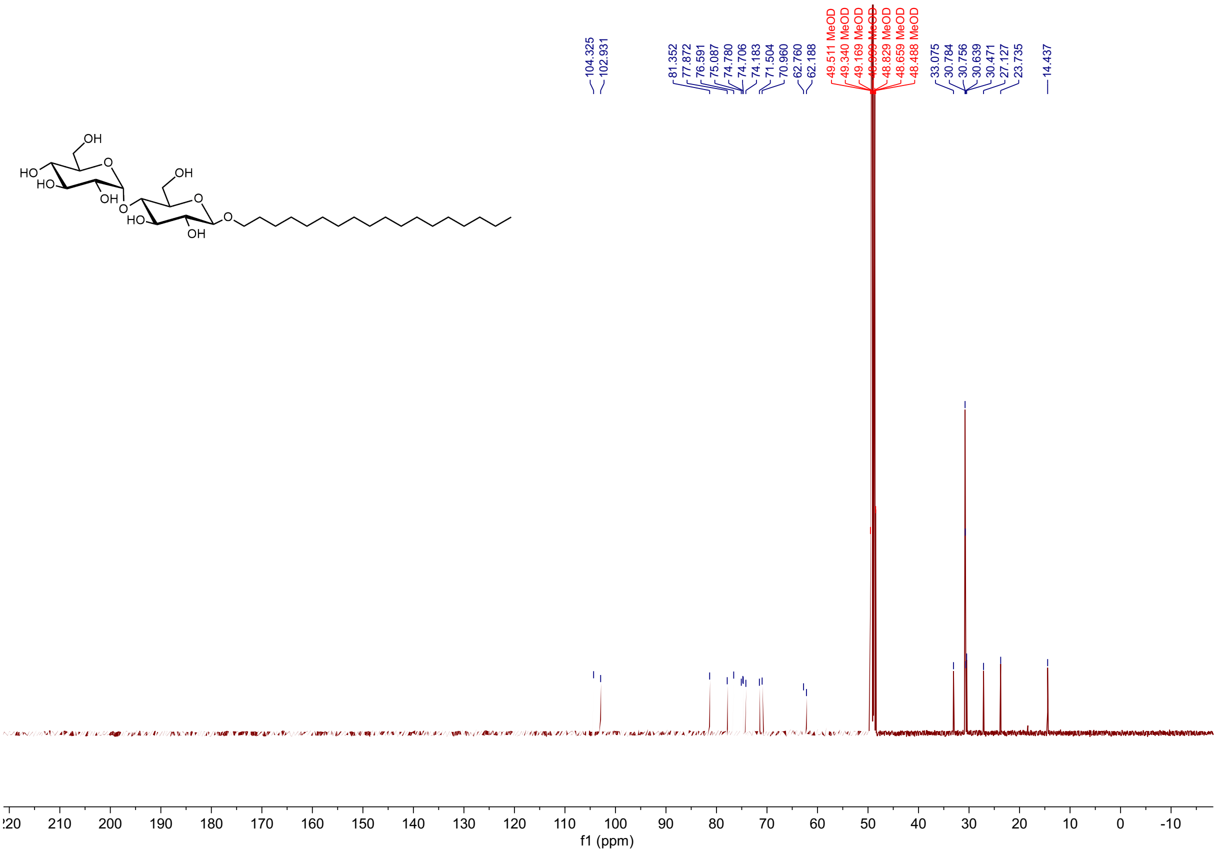

Fig. S34. ^{13}C NMR spectrum of (*9*-octadecynyl)-β-D-maltopyranoside.

3 Methods

Pyrene fluorescence assays were performer on a Probe Drum spectrophotometer (Probation Labs Sweden AB). A solution of pyrene-1-aldehyde in Milli-Q water (100 nM, 1 mL) was added to a cuvette and incubated at 25 °C in a Probe Drum spectrophotometer (Probation Labs Sweden AB). An aliquot of surfactant in Milli-Q water (1.005 mM, 5 μL) was loaded to the sample cell followed by mixing under intermittent stirring for 60s. After equilibration for another 300s, the micelle formation was monitored by fluorescence (λ_{ex} = 377 nm, λ_{em} = 350 – 650 nm) in which two averages were recorded for the emission spectra. The addition-cycle was repeated until a total volume of 100 μL has been added to the sample cell.

Differential scanning calorimetry was performed on a MicroCal VP-DSC instrument. An aliquot of surfactant in Mili-Q water (1.005 mM, 5 μL) was loaded in Tzero pans (TA Instruments) and sealed. Signal was acquired between 5 – 95 °C at a scan rate of 30 °C/h with water as reference.

Dynamic light scattering (DLS) and static light scattering (SLS) measurements were performed using a 3D LS Spectrometer (LS Instruments) equipped with and a goniometric optical setup and a 660 nm Cobolt (100 mW) light source. The instrument implements a modulated 3D cross-correlation scheme to suppress multiple scattering contributions. DLS data was acquired at a fixed angle of 110° (i.e., q = 2.07×10^{-3} Å^{-1}). SLS data was collected within an angular range between 25° and 140°, resulting in a q-range of 6.55×10^{-4} Å^{-1} ≤ q ≤ 2.28×10^{-3} Å^{-1}. The DLS intensity was processed by LS instrument correlators and the resulting intensity auto-correlation function ($g^2(q,t)$ – 1) vs. lag time (t) was exported for analysis. The SLS data was converted to the excess Rayleigh ration ($\Delta R(q)$) using toluene as reference solvent using the equation:

$$\Delta R(q) = \frac{I(q)_{sample}}{I(q)_{toluene}}\left(\frac{n_{D2O}}{n_{toluene}}\right)^2 R_{toluene}$$

where $I(q)_{sample}$ and $I(q)_{toluene}$ are the measured intensities of the sample and the reference respectively, n_{D2O} is the refractive index of D_2O and $n_{toluene}$ is that of our reference, and $R_{toluene}$ is the Rayleigh excess of the reference as determined with our setup (0.8456 × 10^{-5} cm^{-1}). Surfactants were characterised at a concentration of 1 mM in D_2O in SLS mode and DLS of β-$C_{18\text{-}9Z}$Mal in Mili-Q was measured at 0.1, 0.3, 0.5, 1, 2, 4, 6, 8, and 10 mM. The samples were loaded in 10-mm quartz tubes and placed into a temperature-controlled, index-matching vat at 25 °C.

Small-angle neutron scattering (SANS) experiments were performed on Sans2d (ISIS Pulsed Neutron Source, UK) using 5 m and 12 m sample-to-detector distances for the front and rear detector respectively.[10] Using wavelengths between 1.75 and 12.5 Å, the detectors yielded a combined q-range between 0.00156 and 0.5 Å^{-1}. Samples were loaded in 2 mm path length, 1 cm width, quartz Hellma cells and placed in a temperature-controlled sample changer at a temperature of 25 °C. Data reduction was performed using Mantid with the standard procedures of the beamline., accounting for sample transmission, detector efficiency, and the scattering from an empty cell. The scattering of D_2O was subtracted as a background contribution accounting for the incoherent scattering from each sample. The data was absolutely scaled using the scattering from a standard sample (a solid blend of protiated and perdeuterated polystyrene) in accordance with established procedures. The resulting data present the absolute scattered intensities, I(q) in cm^{-1}, versus momentum transfer, q in Å^{-1}. Mathematical models were smeared accounting for the instrument resolution.

For MD Simulations, each surfactant molecule was initially constructed using the Avogadro software.[11] Then, the molecules were parametrised into the GROMOS 54A7 forcefield using the Automated Topology Builder and Repository Server.[12] The structures and corresponding files for Molecular Dynamics simulations are accessible through the following MolIDs: 1260813 for α-$C_{18\text{-}9Z}$Mal, 1260815 for β-$C_{18\text{-}9Z}$Mal, 1260816 for β-$C_{18\text{-}9E}$Mal, and 1260818 for β-$C_{18\text{-}9Y}$Mal. The topology files were then modified to ensure a common nomenclature across all the four molecules.

Four hundred units of each surfactant were then added to a cubic box of 17 nm side filled with SPC water molecules. The neutral systems were subjected to a steepest descent energy minimisation to remove atomic overlaps and clashes, followed by 1 ns NVT equilibration. The production trajectories were carried out at 1 bar and 298 K using the isotropic Parrinello-Rahman barostat and a Nose-Hoover thermostat.[13, 14] The LINCS algorithm was employed to constrain bond lengths,[15] thus eliminating bond vibrations and preventing system drift. The reaction-field method utilizing periodic boundary conditions was used to handle long-range electrostatics with a 1.0 nm cutoff in direct space.[16] Van der Waals interactions were computed using a spherical cut-off of 1.0 nm. Production trajectories of 1.3 μs with a time step of 2 fs for the integration of the motion equations were performed. Energies and positions of atoms were stored every 1000 (2 ps) steps. All MD simulations were performed with the GROMACS 2022.1 software package.[17]

Linear and oscillatory rheological measurements were carried out on Anton Paar MCR 301 at 25 °C using a cone-plate geometry with cone diameter of 25 mm, cone angle of 1°, and a gap of 0.048 mm. The flow curves were recorded in a shear rate range between 0.01 and 100 s^{-1} with 7 measurements per decade. The linear viscoelastic region (LVER) was determined, and subsequent oscillatory measurements were conducted at a constant strain of 10% over the angular frequency range of 0.001–100 rad/s.

4 Data analysis

The DLS intensity autocorrelation functions were fitted using a second-order cumulant function:[18]

$$g_2(q,t) - 1 = B + \beta \left\{ exp(-\Gamma_1 t) \left[1 + \frac{1}{2}\mu_2 t^2 \right] \right\}^2$$

where B represent baseline contributions, β is the spatial coherence factor, Γ_1 is the relaxation rate of the first cumulant, and μ_2 is the second cumulant that accounts for deviations from the single exponential behavior (e.g., polydispersity). The effective particle diffusion (D) was determined from the decay rate using the equation:

$$D = \frac{q^2}{\Gamma_1}$$

It should be noted that the fitting approach only accounts for the first relaxation mode (e.g., particle diffusion, network breathing).[18, 19] Thus, slow relaxation modes (e.g., collective interactions) are not considered here.

Combined data from SANS and SLS measurements were analysed in SasView 5.0.5 using a model-based approach.[20] The Flexible Cylinder model was selected as the most appropriate considering the features of the scattering curves and previous investigations of similar systems.[21] The scattering length density (SLD) of the micelle was calculated from the chemical composition of the protiated tail of the surfactant (i.e., -0.39×10^{-6} Å^{-2}) and held constant. The

SLD of D_2O was fixed to 6.37×10^{-6} Å^{-2}. As the density-pair correlation considered by the model only accounts for the difference between the SLDs of the micelle and the solvent (i.e., $SLD_p - SLD_s$, where *p* and *s* stand for particle and solvent), discrepancies due to e.g., micelle hydration are only expected to cause subtle differences in the volume fraction of particles. That is, no variations in the structural parameters of the scatterers are foreseen. It should be noted that the size of the largest structural feature that can be resolved with the experimental setup used here is ca. 10000 Å ($q_{min} = 6.55\times10^{-4}$ Å^{-1}). Larger values from that are estimated from the modelling of the data. The polydispersity in the radius of the micelle cross-section was fixed to 0.15 (Schulz distribution, number of points = 80, number of Sigmas = 8).[22] Further details on the analysis protocols can be found in previous publications.[23, 24] Fitting optimisation was performed using the Levenberg-Marquardt algorithm implemented in SasView 5.0.3. The tolerance was fixed to 1.5×10^{-8} and the reported uncertainties are within the 95% confidence level ($p = 0.05$).

Analyses of MD simulations data were performed on the final 100 ns of the simulation trajectories to ensure that the systems were well-equilibrated and to capture the relevant dynamic behavior of the surfactant molecules. Snapshots from the MD trajectories were generated using VMD providing a visual inspection of the molecular conformations and interactions.[25] The analysis of the simulations was carried out using the GROMACS *hbond* tool as well as specific code written in Python mainly based on the MDAnalysis,[26] Numpy and Matplotlib libraries.[27, 28]

Oscillatory rheology data were analysed using the Maxwell model,[24] yielding the characteristic relaxation time (τ) and the plateau of the elastic modulus at high frequencies (G'_0). Data were adjusted using the Curve Fitting module implemented in IgorPro 8.04 and the least square algorithm.

5 Supplementary results

5.1 Solubility of glucoside- and maltoside-based surfactants

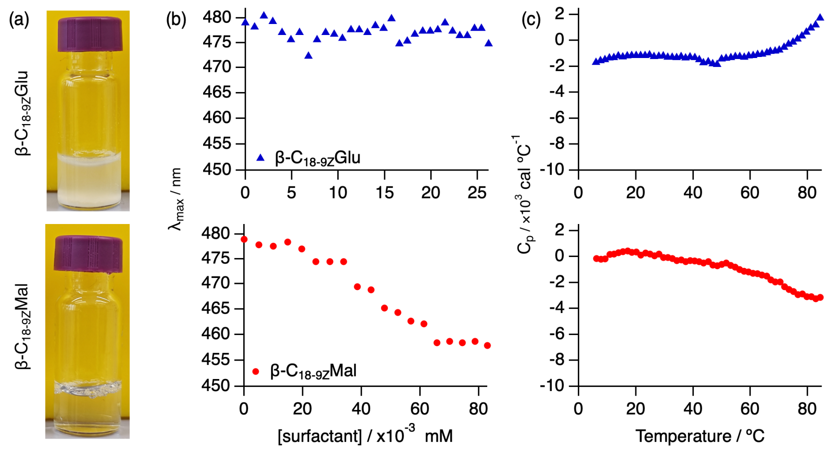

Fig. S35 Comparative behaviour of β-$C_{18\text{-}9Z}$Glu and β-$C_{18\text{-}9Z}$Mal in aqueous solution: (a) Images showing samples at room temperature with 25 mM surfactant concentration, (b) spectroscopic investigation of the CMC of the surfactants, and (c) DSC characterisation of the solubilisation temperature of the surfactants.

5.2 Determination of the solubilisation temperature

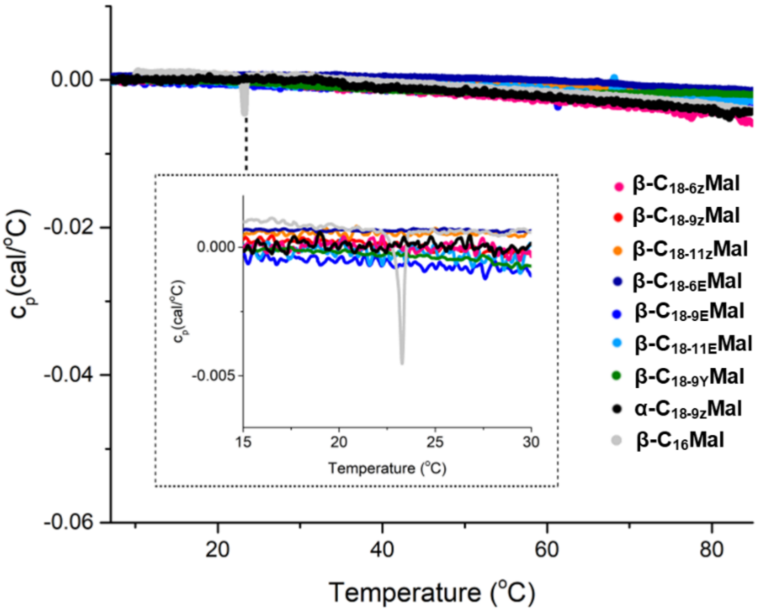

Fig. S36. c_p of sugar-based surfactants in H_2O determined by DSC. The signal from 1 mM β-C_{16}Mal in water was used as a control to confirm the response of the system.[29]

5.3 Critical micelle concentration determination

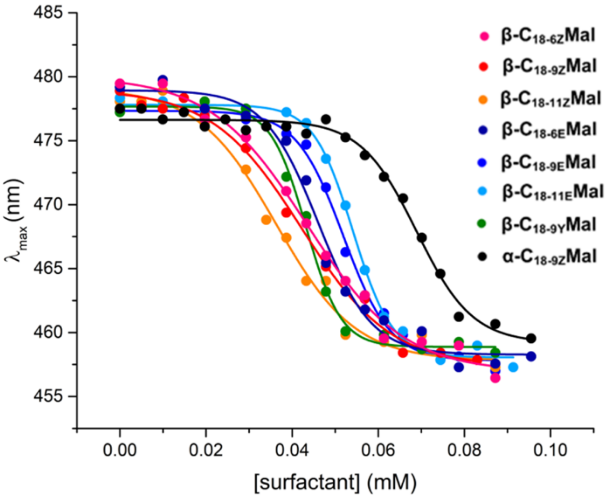

Fig. S37. Evolution of the fluorescence emission maximum of 1-pyrenecarbonxylaldehyde upon titration with the different surfactants as indicated in the legend of the graph. The onset of the early transition (lower surfactant concentration) was used to determine the critical micelle concentration.

5.4 DLS correlation curves

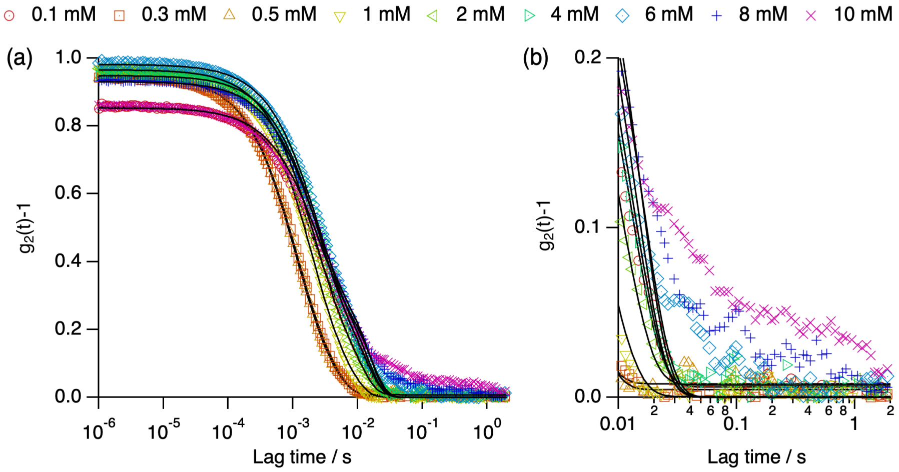

Fig. S38. (a) Intensity autocorrelation function of β-$C_{18\text{-}9Z}$Mal at different concentrations, as indicated in the graph legend, in Mili-Q water. Models are presented as black solid lines. (b) presents the signals obtained at long lag times (> 0.01 s).

5.5 Results from SANS/SLS data analysis

Table S1. Results from the model-based analysis of SANS data of the micelles from the different surfactants at a concentration of 1 mM: ϕ_p – volume fraction of scatterers, r – radius cross-section, l_p – persistence (statistical) length, L – contour length.

Surfactant	ϕ_p / ×10^{-4}	r / Å	l_p / Å	L / Å
**α-$C_{18\text{-}9Z}$Mal**	3.8 ± 0.2	18.9 ± 0.6	252 ± 48	4750 ± 760
**β-$C_{18\text{-}9Z}$Mal**	2.6 ± 0.3	21.5 ± 0.5	123 ± 12	16200 ± 300
**β-$C_{18\text{-}9E}$Mal**	3.9 ± 0.2	21.9 ± 0.7	291 ± 26	28600 ± 700
**β-$C_{18\text{-}9Y}$Mal**	3.2 ± 0.2	19.8 ± 0.4	134 ± 17	9360 ± 110
**β-$C_{18\text{-}6Z}$Mal**	2.9 ± 0.1	20.8 ± 0.4	220 ± 11	16200 ± 600
**β-$C_{18\text{-}11Z}$Mal**	3.4 ± 0.2	22.0 ± 0.3	270 ± 20	21000 ± 600
**β-$C_{18\text{-}6E}$Mal**	4.1 ± 0.1	22.1 ± 0.5	316 ± 21	22400 ± 700
**β-$C_{18\text{-}11Z}$Mal**	3.2 ± 0.1	22.3 ± 0.5	370 ± 12	23300 ± 700

5.6 MD simulation snapshots

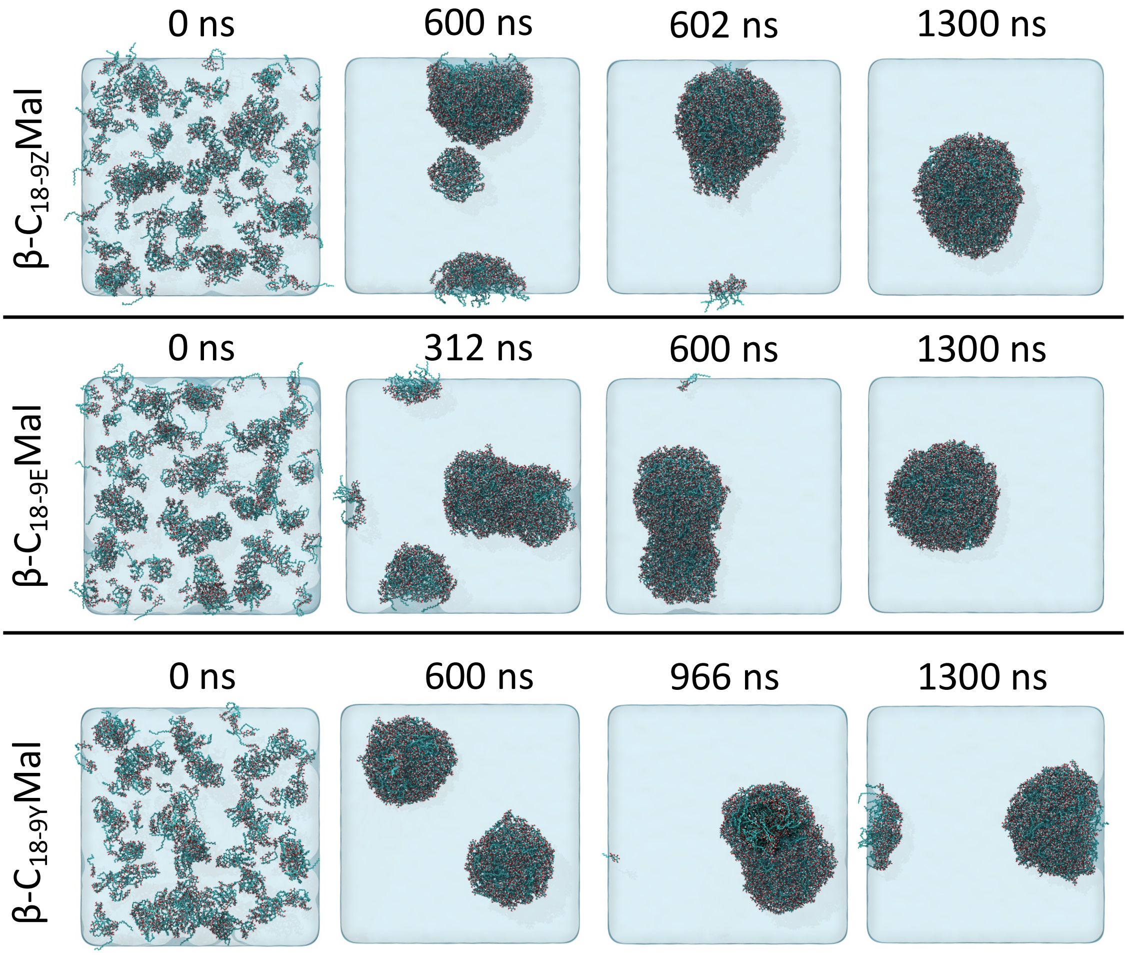

Fig. S39. (a) Representative snapshots of the MD simulation trajectory of different surfactants conducted at 1 bar and 298.15 K.

5.7 Linear rheology results

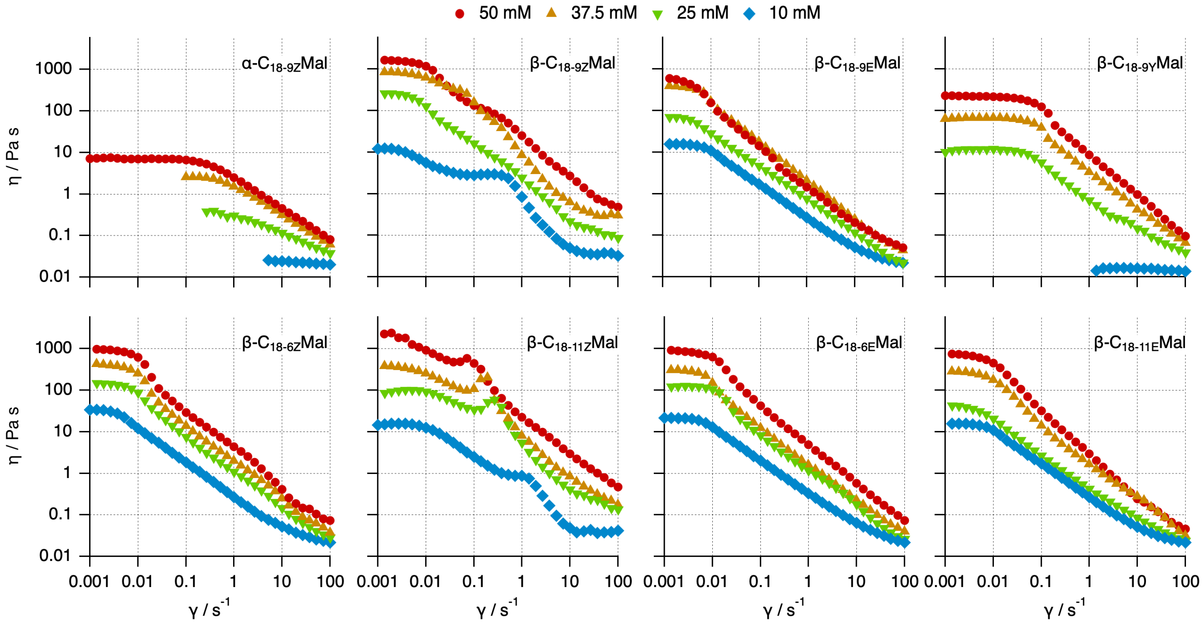

Fig. S40. Linear viscosity ramps as a function of the shear rate for the surfactants at different concentrations in aqueous solution as indicated in the graph legend.

5.8 Oscillatory rheology results

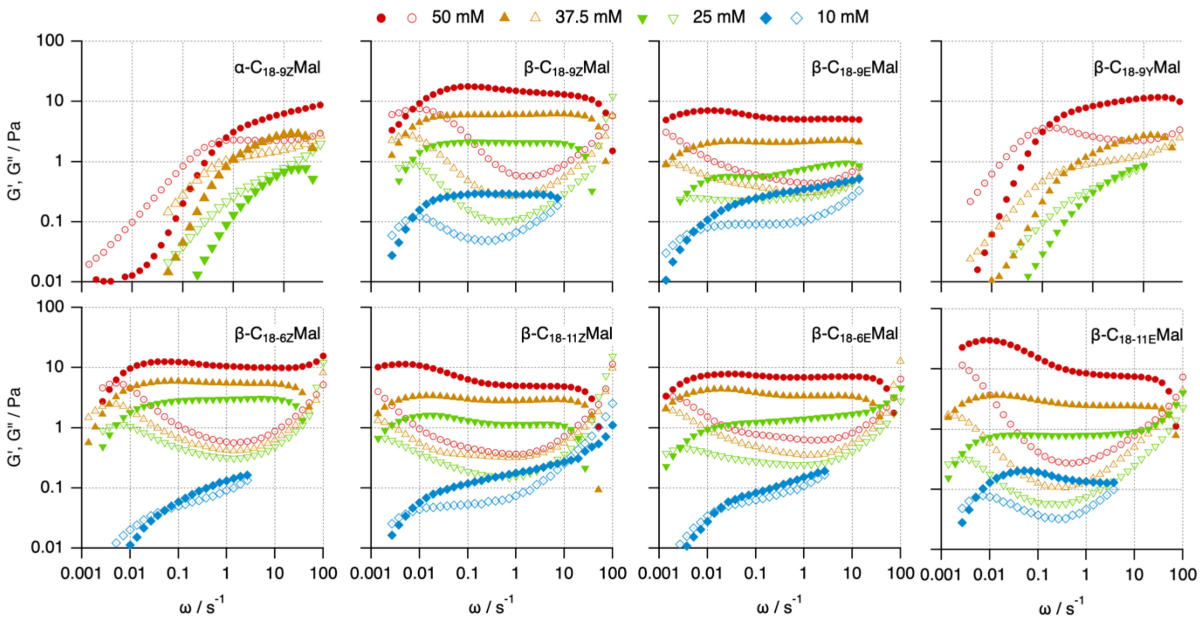

Fig. S41. Oscillatory shear frequency-sweep results (strain of 10%) for the surfactants at different concentrations in aqueous solution as indicated in the graph legend. Data are represented as the elastic (G', open symbols) and viscous (G'', filled symbols) moduli as a function of frequency. Surfactant concentrations are indicated on the graph legend.

5.9 Cole-Cole plots

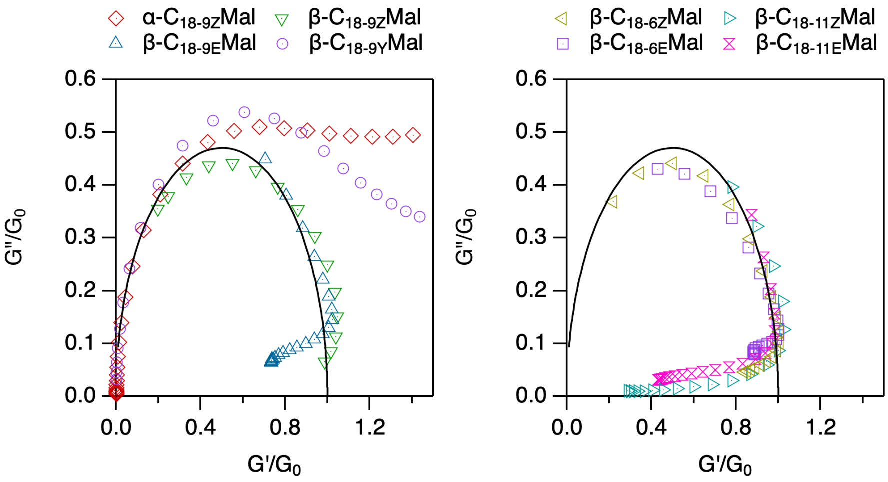

Fig. S42. Normalised Cole-Cole plots obtained from the shear frequency-sweep results (strain of 10%) for 50 mM of surfactants as indicated in the legend of the graphs.

6 Supporting references

1. P. E. Duffy, S. M. Quinn, H. M. Roche and P. Evans, *Tetrahedron*, 2006, **62**, 4838-4843.

2. S. Ortgies, R. Rieger, K. Rode, K. Koszinowski, J. Kind, C. M. Thiele, J. Rehbein and A. Breder, *ACS Catalysis*, 2017, **7**, 7578-7586.

3. M. Overs, M. Fix, S. Jacobi, L. F. Chi, M. Sieber, H.-J. Schäfer, H. Fuchs and H.-J. Galla, *Langmuir*, 1999, **16**, 1141-1148.
4. J. Yue and S. S. Narine, *Chem. Phys. Lipids*, 2008, **152**, 1-8.
5. C. Koga, M. Kohri, T. Taniguchi and K. Kishikawa, *J. Phys. Chem. B*, 2019, **123**, 4324-4332.
6. S. Narasimhan, K. G. Prasad and S. Madhavan, *Tetrahedron Lett.*, 1995, **36**, 1141-1144.
7. T. Zheng, R. S. Narayan, J. M. Schomaker and B. Borhan, *J. Am. Chem. Soc.*, 2005, **127**, 6946-6947.
8. A. Sytniczuk, M. Dabrowski, L. Banach, M. Urban, S. Czarnocka-Sniadala, M. Milewski, A. Kajetanowicz and K. Grela, *J. Am. Chem. Soc.*, 2018, **140**, 8895-8901.
9. S.-S. Kim and Y. P. Hong, *Bull. Korean Chem. Soc.*, 2011, **32**, 3120-3122.
10. R. K. Heenan, S. E. Rogers, D. Turner, A. E. Terry, J. Treadgold and S. M. King, *Neutron News*, 2011, **22**, 19-21.
11. M. D. Hanwell, D. E. Curtis, D. C. Lonie, T. Vandermeersch, E. Zurek and G. R. Hutchison, *J Cheminform*, 2012, **4**, 17.
12. N. Schmid, A. P. Eichenberger, A. Choutko, S. Riniker, M. Winger, A. E. Mark and W. F. van Gunsteren, *Eur. Biophys. J.*, 2011, **40**, 843-856.
13. M. Parrinello and A. Rahman, *J. Appl. Phys.*, 1981, **52**, 7182-7190.
14. D. J. Evans and B. L. Holian, *J. Chem. Phys.*, 1985, **83**, 4069-4074.
15. B. Hess, H. Bekker, H. J. C. Berendsen and J. G. E. M. Fraaije, *J. Comput. Chem.*, 1997, **18**, 1463-1472.
16. P. H. Hünenberger and W. F. van Gunsteren, *J. Chem. Phys.*, 1998, **108**, 6117-6134.
17. M. J. Abraham, T. Murtola, R. Schulz, S. Páll, J. C. Smith, B. Hess and E. Lindahl, *SoftwareX*, 2015, **1-2**, 19-25.
18. A. Gulotta, M. Polimeni, S. Lenton, C. G. Starr, A. Stradner, E. Zaccarelli and P. Schurtenberger, *Mol. Pharm.*, 2024, **21**, 2250-2271.
19. A. Sanchez-Fernandez, J. Larsson, A. E. Leung, P. Holmqvist, O. Czakkel, T. Nylander, S. Ulvenlund and M. Wahlgren, *Langmuir*, 2022, **38**, 10075-10080.
20. M. Doucet, J. H. Cho, G. Alina, Z. Attala, J. Bakker, W. Bouwman, P. Butler, K. Campbell, T. Cooper-Benun, C. Durniak, L. Forster, M. Gonzales, R. Heenan, A. Jackson, S. King, P. Kienzle, J. Krzywon, T. Nielsen, L. O'Driscoll, W. Potrzebowski, S. Prescott, R. Ferraz Leal, P. Rozycko, T. Snow and A. Washington, SasView version 5.0.3, https://zenodo.org/record/3930098, (accessed 2020-08-24, DOI: 10.5281/ZENODO.3930098).
21. J. S. Pedersen and P. Schurtenberger, *Macromolecules*, 1996, **29**, 7602-7612.
22. M. Kotlarchyk, R. B. Stephens and J. S. Huang, *J. Phys. Chem.*, 2002, **92**, 1533-1538.
23. J. Larsson, A. Sanchez-Fernandez, N. Mahmoudi, L. C. Barnsley, M. Wahlgren, T. Nylander and S. Ulvenlund, *Langmuir*, 2019, **35**, 13904-13914.
24. J. Larsson, A. Sanchez-Fernandez, A. E. Leung, R. Schweins, B. Wu, T. Nylander, S. Ulvenlund and M. Wahlgren, *J. Colloid Interface Sci.*, 2021, **581**, 895-904.
25. W. Humphrey, A. Dalke and K. Schulten, *J Mol Graph*, 1996, **14**, 33-38, 27-38.

26. N. Michaud-Agrawal, E. J. Denning, T. B. Woolf and O. Beckstein, *J. Comput. Chem.*, 2011, **32**, 2319-2327.

27. J. D. Hunter, *Computing in Science & Engineering*, 2007, **9**, 90-95.

28. C. R. Harris, K. J. Millman, S. J. van der Walt, R. Gommers, P. Virtanen, D. Cournapeau, E. Wieser, J. Taylor, S. Berg, N. J. Smith, R. Kern, M. Picus, S. Hoyer, M. H. van Kerkwijk, M. Brett, A. Haldane, J. F. Del Rio, M. Wiebe, P. Peterson, P. Gerard-Marchant, K. Sheppard, T. Reddy, W. Weckesser, H. Abbasi, C. Gohlke and T. E. Oliphant, *Nature*, 2020, **585**, 357-362.

29. J. Larsson, A. E. Leung, C. Lang, B. Wu, M. Wahlgren, T. Nylander, S. Ulvenlund and A. Sanchez-Fernandez, *J. Colloid Interface Sci.*, 2021, **585**, 178-183.
